# Using vertebrate environmental DNA from seawater in biomonitoring of marine habitats

**DOI:** 10.1111/cobi.13437

**Published:** 2019-12-24

**Authors:** Eva Egelyng Sigsgaard, Felipe Torquato, Tobias Guldberg Frøslev, Alec B. M. Moore, Johan Mølgård Sørensen, Pedro Range, Radhouane Ben‐Hamadou, Steffen Sanvig Bach, Peter Rask Møller, Philip Francis Thomsen

**Affiliations:** ^1^ Natural History Museum of Denmark University of Copenhagen Universitetsparken 15 DK‐2100 Copenhagen Ø Denmark; ^2^ Section for GeoGenetics, Globe Institute, University of Copenhagen, Øster Voldgade 5‐7, DK‐1350 Copenhagen K, Denmark (previously: Centre for GeoGenetics, Natural History Museum of Denmark); ^3^ School of Ocean Sciences, Bangor University Menai Bridge Anglesey LL59 5AB U.K.; ^4^ Environmental Science Center Qatar University P.O. Box 2713 Doha Qatar; ^5^ Department of Biological and Environmental Sciences Qatar University P.O. Box 2713 Doha Qatar; ^6^ Maersk Oil Research and Technology Centre Al Jazi Tower, Building 20, Zone 60, Street 850, West Bay Doha Qatar

**Keywords:** Arabian Gulf, biomonitoring, fish, metabarcoding, eDNA, biomonitoreo, Golfo Arábigo, meta‐código de barras, peces, ADNa, 生物监测, DNA 条形码技术, 阿拉伯湾, 鱼类

## Abstract

Conservation and management of marine biodiversity depends on biomonitoring of marine habitats, but current approaches are resource‐intensive and require different approaches for different organisms. Environmental DNA (eDNA) extracted from water samples is an efficient and versatile approach to detecting aquatic animals. In the ocean, eDNA composition reflects local fauna at fine spatial scales, but little is known about the effectiveness of eDNA‐based monitoring of marine communities at larger scales. We investigated the potential of eDNA to characterize and distinguish marine communities at large spatial scales by comparing vertebrate species composition among marine habitats in Qatar, the Arabian Gulf (also known as the Persian Gulf), based on eDNA metabarcoding of seawater samples. We conducted species accumulation analyses to estimate how much of the vertebrate diversity we detected. We obtained eDNA sequences from a diverse assemblage of marine vertebrates, spanning 191 taxa in 73 families. These included rare and endangered species and covered 36% of the bony fish genera previously recorded in the Gulf. Sites of similar habitat type were also similar in eDNA composition. The species accumulation analyses showed that the number of sample replicates was insufficient for some sampling sites but suggested that a few hundred eDNA samples could potentially capture >90% of the marine vertebrate diversity in the study area. Our results confirm that seawater samples contain habitat‐characteristic molecular signatures and that eDNA monitoring can efficiently cover vertebrate diversity at scales relevant to national and regional conservation and management.

## Abstract 2

*Article impact statement*: Environmental DNA provides habitat‐characteristic molecular signatures and can be used efficiently to map marine biodiversity at large spatial scales.

## Abstract (es)

ADN Ambiental de Vertebrados Tomado del Agua Marina para Realizar Biomonitoreos de los Hábitats Marinos

**Resumen**

La conservación y el manejo de la biodiversidad marina depende del biomonitoreo de los hábitats marinos, pero las estrategias actuales requieren de muchos recursos y de diferentes estrategias para diferentes organismos. El ADN ambiental (ADNa) extraído de muestras de agua es una estrategia eficiente y versátil para detectar animales acuáticos. En el océano, la composición del ADNa refleja la fauna local a escalas espaciales finas, pero se sabe poco sobre la efectividad del monitoreo basado en el ADNa de las comunidades marinas a grandes escalas. Investigamos el potencial del ADNa para caracterizar y distinguir las comunidades marinas a escalas espaciales grandes mediante una comparación de la composición de especies de vertebrados entre los hábitats marinos de Qatar, en el Golfo Arábigo (también conocido como el Golfo Persa), con base en el meta‐código de barras del ADNa extraído de muestras de agua de mar. Realizamos análisis de acumulación de especies para estimar cuánta de la diversidad de vertebrados logramos detectar. Obtuvimos secuencias de ADNa de diversos ensamblajes de vertebrados marinos, los cuales abarcaron 191 taxones de 73 familias. Estos taxones incluyeron a especies raras y en peligro de extinción y cubrieron el 36% de los géneros de peces óseos previamente registrados en el golfo. Los sitios con tipos similares de hábitat también fueron similares en cuanto a la composición del ADNa. Los análisis de acumulación de especies mostraron que el número de réplicas de muestras fue insuficiente para algunos sitios de muestreo, pero sugieren que unos cientos de muestras de ADNa podrían capturar potencialmente >90% de la diversidad de vertebrados marinos en el área de estudio. Nuestros resultados confirman que las muestras de agua marina contienen firmas moleculares características del hábitat y que el monitoreo de ADNa puede cubrir eficientemente la diversidad de vertebrados a escalas relevantes para la conservación y el manejo nacional y regional.

## 摘要

海洋生物多样性的保护和管理依赖于对海洋生境的生物监测, 而目前的监测方法都属于资源密集型, 且对不同生物需要采取不同的方法。从水样中提取环境 DNA (eDNA) 是一种调查水生动物高效通用的方法。在海洋中, eDNA 的组成反映了区域精细空间尺度上动物群的情况, 但对于 eDNA 在更大尺度上调查海洋生物群落的有效性尚有待探究。本研究基于 eDNA 条形码技术在海水样品中的应用, 比较了卡塔尔和阿拉伯湾 (又称波斯湾) 海洋生境的脊椎动物组成, 以分析 eDNA 在大空间尺度上描述和分辨海洋生物群落的能力。我们通过物种累积分析估计了我们检测到的脊椎动物多样性。结果显示, 我们获得的 eDNA 序列来自包含 73 个科 191 个类群的海洋脊椎动物群集。这些动物中包括稀有物种和濒危物种, 覆盖了该海湾现有记录中 36% 的硬骨鱼属。生境类型相近的位点检测到的 eDNA 组成也相似。物种累积分析还表明一些采样位点的样品重复个数不足, 但几百个 eDNA 样本可能足够捕获该研究地区 90% 以上的海洋脊椎动物多样性。我们的研究结果证实了海水样本中含有具有生境特点的分子信号, 且 eDNA 检测可以在与国家和区域保护管理所涉及的尺度上有效覆盖脊椎动物多样性。**【翻译: 胡怡思; 审校: 聂永刚】**

## Introduction

Biomonitoring of marine habitats is essential for marine ecological research and for the conservation and management of marine biodiversity. In many areas, marine biodiversity remains poorly known, even for more well‐studied taxonomic groups, such as vertebrates (e.g., Buchanan et al. 2016). Furthermore, biodiversity needs to be monitored continuously to be able to follow the effects of environmental changes, but traditional biomonitoring surveys are costly and time‐consuming, often limited to a single taxonomic group (Watson et al. 2005), and typically require taxonomic expertise, which is increasingly scarce and declining (Hopkins & Freckleton 2002). These limitations also complicate standardization across space and personnel. Biomonitoring and habitat characterization based on environmental DNA (eDNA) extracted from seawater samples could overcome some of these challenges (Thomsen & Willerslev 2015; Sigsgaard et al. 2019). Analyses of eDNA in seawater can cover a broad range of taxonomic groups simultaneously (e.g., Stat et al. 2017), opening up a potential for a much more efficient and standardized monitoring of marine taxa. Importantly, eDNA composition also reflects local community composition at scales of tens to a few thousand meters (e.g., Kelly et al. 2016; Port et al. 2016; Jeunen et al. 2019), potentially allowing for eDNA biomonitoring at fine spatial scales. However, it remains to be tested whether eDNA‐based community analyses can be informative for marine habitat biomonitoring at large (tens to hundreds of kilometers) spatial scales.

We investigated marine vertebrate diversity of Qatar, the Arabian Gulf (also known as the Persian Gulf; hereafter “The Gulf”), through eDNA metabarcoding of seawater samples to test the potential of eDNA for characterizing and distinguishing marine vertebrate communities at a regional scale. We compared vertebrate eDNA composition among different habitat types with clustering and ordination analyses, compared eDNA results with traditional monitoring data, and estimated how well diversity is covered with species‐accumulation modeling. We hypothesized that because eDNA composition in seawater appears to reflect local species composition, study sites of the same habitat type are more similar in eDNA composition than sites of different habitat types. The alternative hypothesis was that eDNA in seawater is extensively dispersed by currents and wave action and thus eDNA composition is homogenized such that communities in different habitats appear similar when in close geographical proximity.

## Methods

### Sample Collection and Visual Census

**Figure 1:**
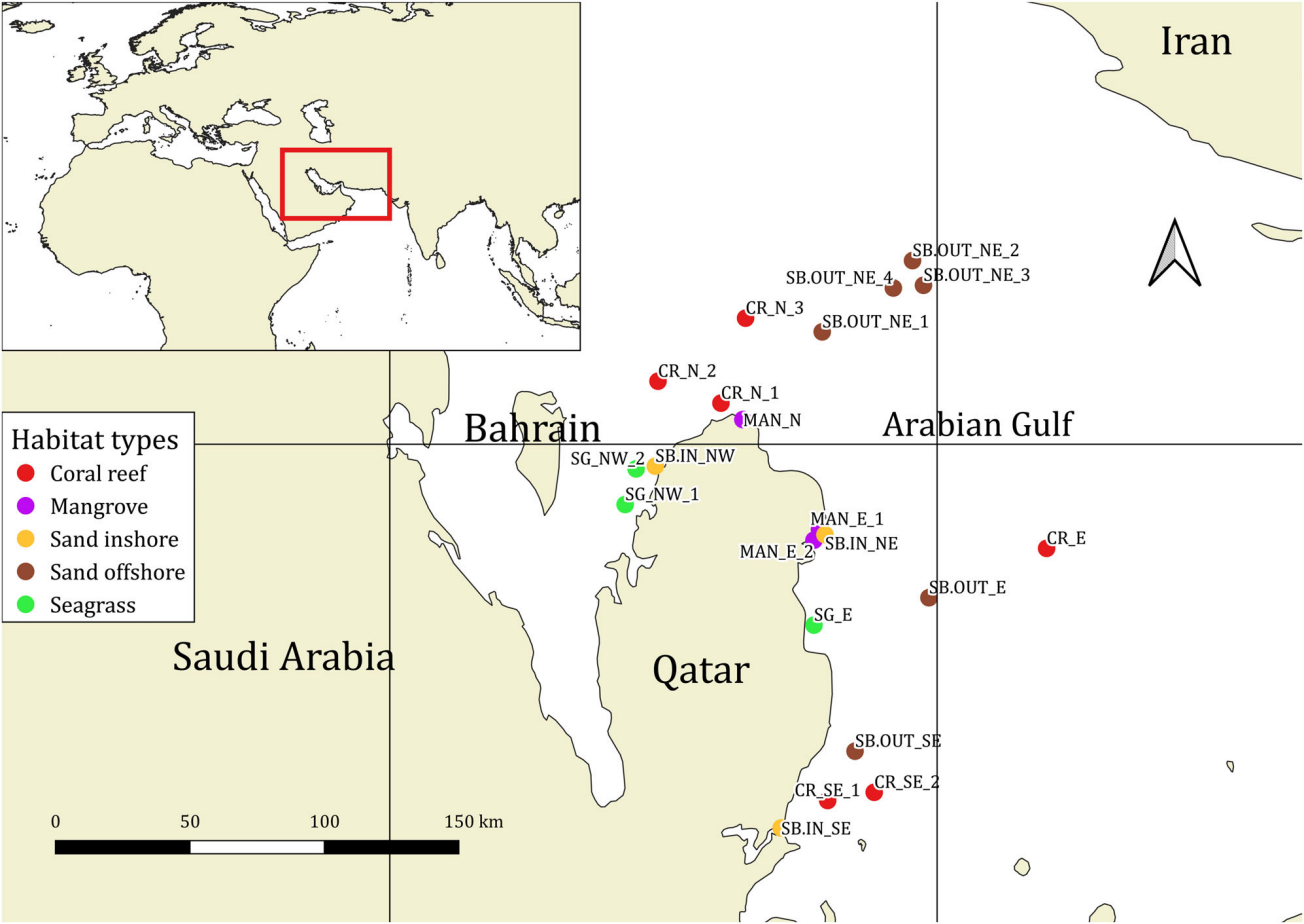
Location of sampling sites in the Arabian Gulf.

We collected seawater samples of 3 × 1‐L from 21 sites around Qatar in the Arabian Gulf (Fig. 1 & Supporting Information). The sites were chosen to cover 5 habitat types: seagrass beds, coral reefs, mangroves, inshore sand bottom, and offshore sand bottom (open water). Each habitat type was represented by at least 3 sites. Each 1‐L sample was collected at the surface by repeatedly filling a 150‐mL luer‐lock syringe (Monoject, Covidien) and immediately filtering the collected seawater through the same sterile 0.22 µm Sterivex‐GP filter (Merck Millipore, Germany). Facemasks and nitrile gloves were worn during sample collection and changed between each sampling site. Syringes were changed between sampling sites and were thoroughly rinsed in tap water and air dried before any reuse on subsequent sampling days. At the 3 offshore sand bottom sites in the Al Shaheen oil field (SB.OUT_NE_2, SB.OUT_NE_3, SB.OUT_NE_4), additional 3 × 1‐L samples were collected from a depth of approximately 30 m with a 1.7‐L Ruttner water sampler (model number 11.002) because fish abundance at the platforms appears to peak at around 21–30 m depth (Torquato et al. 2017). All sites were sampled twice, summer 2016 and spring 2017, to obtain a greater coverage of biodiversity, especially of species with seasonal migrations. In total, 24 triplicate eDNA samples (72 individual filter samples) were collected in each of the 2 years. Field negative control samples consisting of 500 mL of bottled mineral water were collected by filtering as described above (Supporting Information). Samples were stored on ice during transport and subsequently frozen at −18 °C. At 9 sampling sites, we conducted visual censuses. While snorkeling or scuba diving, we took pictures or video of vertebrates (Supporting Information). At some sites, visual census was not possible or of limited success due to poor underwater visibility.

### Extraction, PCR Amplification, and Sequencing of eDNA

All laboratory work was conducted in the laboratories at the Centre for GeoGenetics, Natural History Museum of Denmark. Environmental DNA was extracted from filters with the DNeasy Blood and Tissue kit (Qiagen, Germantown, Maryland). We modified the manufacturer's spin column protocol as follows. Initial incubation was done for 3 h with rotation, the volumes of ATL buffer, proteinase K, AL buffer, and ethanol were increased 4‐fold, and DNA was eluted in 2 × 60 µL AE buffer with incubation for 2 × 10 min at 37 °C. Extraction blanks were included throughout the process. Polymerase chain reaction (PCR) amplification was performed using 2 primer sets targeting the mitochondrial 12S gene: the MiFish primers that target fish (Miya et al. 2015) and a primer set designed by Riaz et al. (2011) that targets vertebrates (hereafter referred to as the Riaz primers). The MiFish primers provide good taxonomic resolution for fishes (Taberlet et al. 2018) and can capture elasmobranchs (MiFish‐E) as well as bony fishes (MiFish‐U). The Riaz primers provide lower resolution, but were expected to better capture eDNA from vertebrates other than fish. Several PCR negative controls were included in each setup, along with a mock sample made from tissue‐extracted DNA of 10 Danish freshwater fishes (Supporting Information) to test the bioinformatic error filtering pipeline. Libraries were prepared using the TruSeq DNA PCR‐free LT Sample Prep kit (Illumina, San Diego, California) and sequenced on the NextSeq 500 platform (Illumina) with 150 bp paired‐end sequencing. To improve the taxonomic coverage of the GenBank nt database as a reference for our study, tissue samples from >80 fish species were sequenced for the 12S metabarcode regions. Further details on laboratory methods are given in the Supporting Information.

### Bioinformatic Analyses

Using the software packages Cutadapt (Martin 2011) and VSEARCH (Rognes et al. 2016), we demultiplexed Illumina reads and removed reads that were below 10 bases in length, included N's, or contained >2 expected errors (scripts are available at https://github.com/tobiasgf/Bioinformatic-tools/tree/master/Eva_Sigsgaard_2018). Reads were truncated at the first instance of a quality score of ≤2, and error filtering was then done separately for sense and antisense reads within each fastq file using DADA2 (Callahan et al. 2016). Matching forward and reverse reads with a minimum overlap of 5 bp and no mismatches were then merged. Likely chimeras were removed based on a consensus over all samples. The sequences were then searched remotely against the NCBI nt database on 10 June 2018 with BLASTn (Altschul et al. 1990). We requested a maximum of 40 aligned sequences per query, minimum thresholds of 90% query coverage per high‐scoring segment pair, and 80% sequence similarity. The BLAST hits displaying an incomplete final coverage of the query sequence were removed, and the remaining hits were classified taxonomically in the R package taxize (Chamberlain & Szocs 2013). For query sequences with a best BLASTn hit of <100% identity, we used similarity thresholds calculated following Alberdi et al. (2018) to help determine the appropriate level of taxonomic assignment (Supporting Information).

For each barcode, the average percentage of identity between sequences from the same genus, family, and order, respectively, was calculated based on all the BLASTn hits obtained for the eDNA sequences. The 95 percentiles of these values were then set as thresholds for assigning sequences to species, genus, and family level, respectively. Guideline similarity thresholds for the MiFish barcode were thus set at >89%, >93%, and 99–100% identity for family‐, genus‐ and species‐level assignment, respectively (Supporting Information). For the Riaz barcode, the corresponding thresholds were >93%, >96%, and 99–100% (Supporting Information). Sequences were therefore assigned to species only if at least one of the barcodes gave a 99–100% match to the species. We examined taxonomic identifications of sequences manually to check for errors, such as erroneous taxonomic identification of GenBank sequences (Ashelford et al. 2005). If an eDNA sequence had several BLAST hits that were tied for best sequence similarity, but only one hit was to a taxon known to be present in the Gulf, the sequence was assigned to the regionally present taxon (Supporting Information). Only sequences identified to at least family were taken into account in further analyses.

Sequences assigned to the same taxon were collapsed by summing the read counts, and the final OTU table and taxonomic assignments were imported to R with ROBITools (LECA 2012). The total detected diversity was visualized using the R packages metacoder (Foster et al. 2017) and taxa (Foster et al. 2018).

For each of the 4 data sets (2 barcodes and 2 sampling years), sequences occurring in higher frequency in the negative controls than in a water sample were removed (Taberlet et al. 2018). The DNA sequences from domestic animals, which are common contaminants in eDNA studies, were also removed from the data (Supporting Information). Three sequences, identified as Pleuronectidae sp., Cyprinidae sp., and *Leucaspius delineates*, were removed from further analyses because they were deemed highly likely to be contaminations from previous work in our laboratory. Finally, sequences appearing only in a single PCR replicate were discarded. To assess the representativeness of the sequencing data for the eDNA samples, species accumulation curves were produced for the PCR replicates of each water sample (Supporting Information).

### Ecological Analyses

The data from the MiFish and Riaz barcodes were combined for the ecological analyses to obtain a more robust data set (sequences identified as the same taxon were merged by summing the read counts). The total detected diversity was compared with the fish fauna known from the literature and with the diversity detected through visual census. Results from eDNA were also compared with data from a trawling expedition conducted in the central Gulf in 1990 by M. Andersen and A. Redsted Rasmussen of the Zoological Museum, University of Copenhagen.

The sequence data from each water sample were then rarefied (resampled) to the median read count observed for a PCR (de Cárcer et al. 2011) to yield more reliable comparisons between sampling sites. The species composition of the sampling sites based on eDNA was compared by average‐linkage hierarchical clustering with the Raup–Crick dissimilarity index (presence‐absence‐based index [Raup & Crick 1979]) calculated with the raupcrick function in vegan version 2.4‐6 (Oksanen et al. 2018). The default null model was used with 9999 simulations. An additional clustering analysis that included read abundance information was done using vegdist in vegan and the Bray–Curtis index. To investigate whether the sampled habitat types were distinctive, we performed a permutational analysis of variance (PERMANOVA) test based on the Raup–Crick index and a canonical analysis of principal coordinates (CAP) (Anderson & Willis 2003) in BiodiversityR (Kindt & Coe 2005) with the Bray–Curtis index. Because the Raup–Crick dissimilarity data did not meet the assumption of multivariate homogeneity of group dispersions (permutation test, *p* < 0.05), the data were transformed using inverse normal transformation.

**Figure 2:**
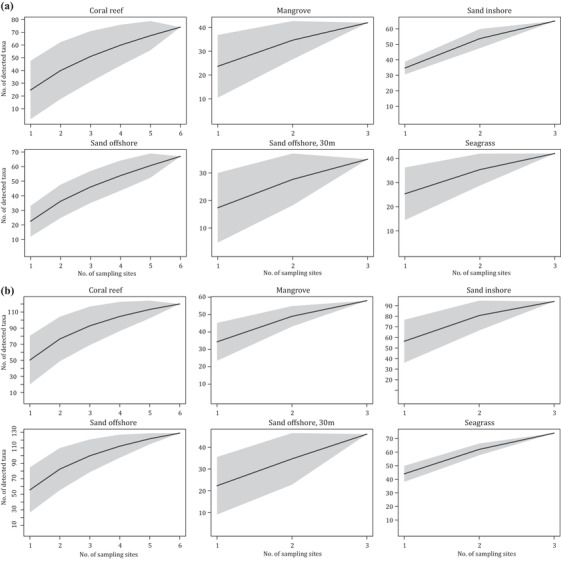
Species accumulation in 6 habitat types in (a) 2016 and (b) 2017 (gray shading, 95% CI based on the unconditional SD).

Species accumulation curves were plotted for each sampling site and habitat type (Fig. 2 & Supporting Information) and for the entire study area. Four nonlinear regression models (Arrhenius, Gleason, Gitay, and Lomolino), which have all been suggested as suitable models for species–area relationships (Dengler 2009), were fitted to the accumulation curve for the entire study area. The model with the lowest AIC was used to estimate the total vertebrate diversity of the sampled area and to extrapolate the accumulation curve to 200 sampling sites. Because results varied among different runs of rarefaction, results from 4 runs of rarefaction were considered. All analyses were conducted in R version 3.5 (R Core Team 2019). See the Supporting Information for further details on the ecological analyses.

## Results

### Detected Vertebrate Diversity from eDNA

**Figure 3:**
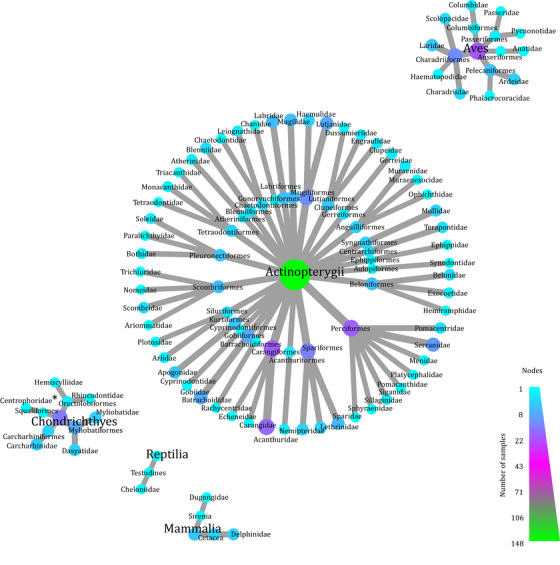
Taxonomic tree of all vertebrate families detected via eDNA across habitat types and sampling seasons. Node color and size reflect the number of eDNA samples each taxon was detected in (^*^, detection should be interpreted with care because the detected fish, *Deania* sp., is a deep‐sea shark and neither *Deania* nor fish in the family Centrophoridae have to our knowledge ever been recorded in the Arabian Gulf).

Illumina sequencing yielded 175 and 138 million reads in total for 2016 and 2017, respectively. After filtering and manual curation, eDNA samples yielded 11,162,176 (mean of 465,091 [SE 91,468] per sample) and 16,775,135 (698,964 [121,450] per sample) reads for 2016 and 2017, respectively. Most samples appeared to approach saturation of diversity with 4 PCR replicates (Supporting Information), whereas accumulation curves for sampling sites rarely saturated at 3 water samples (Supporting Information). A final list of 191 taxa, belonging to 73 families of vertebrates, was obtained (Fig. 3 & Supporting Information). Fifteen cartilaginous fish taxa and 148 bony fish taxa were detected. Twenty‐two bird taxa, 1 reptile, and 5 marine mammals were detected.

The PCR negative controls contained only cow DNA (*Bos* sp.), which may stem from PCR consumables. The 3 taxa detected in extraction controls (*Aphanius dispar, Rhabdosargus haffara*, and *Gerres* sp. 1) were always present at lower abundance than in the eDNA samples and were therefore retained in the data. After filtration, mock sample PCRs retained 9–15 unique sequences. With 4 exceptions, where white bream (*Blicca bjoerkna*) was absent, all 10 added species were retrieved. We obtained 292 reads with hits to fish species not included in the mock (rainbow trout [*Onchorhynchus mykiss*], Wels catfish [*Silurus glanis*], *Alosa* sp., boarfish [*Capros aper*], beluga [*Huso huso*], river lamprey [*Lampetra fluviatilis*], and shorthorn sculpin [*Myoxocephalus scorpius*]). Only *O. mykiss* appeared with >10 reads in a library. This species was present in 13 libraries, indicating a possible contamination in one of the tissue extracts. The remaining sequences constituted <0.1% of the mock sample reads and likely represented point contaminations or errors. Overall, the output from the mock sample thus closely represented the known sequence composition of the sample, indicating that erroneous sequences had been efficiently removed. The read counts from the mock sample also showed a positive linear relationship with the DNA concentrations of the added tissue extracts (*p* < 0.01, *R* ^2^ > 0.7) (Supporting Information).

Thirteen taxa, including several marine fishes known to occur in the Gulf, were removed from the 2016 data set because they were more or equally abundant in field controls than in the seawater samples. The removal of sequences appearing in a single PCR replicate excluded a number of likely contaminants from the data and several taxa that could be true detections as they have previously been recorded in the Gulf (Supporting Information).

### Comparisons with Visual Census and Trawl Data

**Figure 4:**
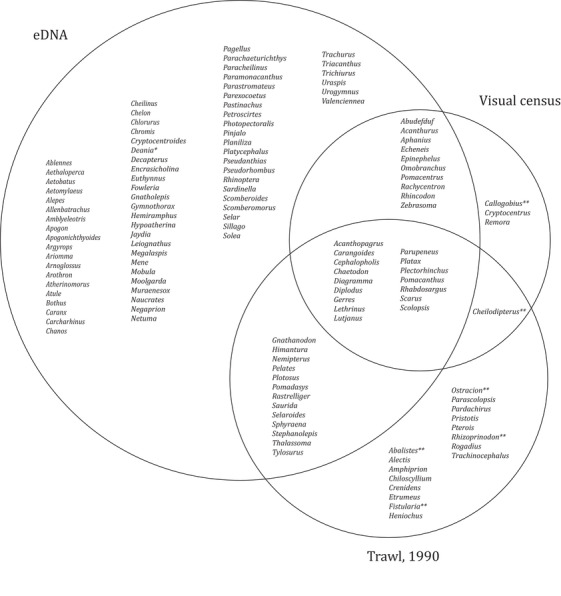
Overlap in fish detections at genus level between eDNA results, visual census results obtained in parallel with eDNA sampling, and results from a trawling expedition in the central Gulf in 1990 (^*^, detection should be interpreted with care because *Deania* is a genus of deep‐sea sharks and neither fish in the genus nor family Centrophoridae have to our knowledge been recorded in the Gulf; ^**^, detected with eDNA in a single PCR replicate).

Visual census yielded photo or video documentation of 36 fish species from 33 genera (Fig. 4 & Supporting Information). The highest diversity was observed at offshore reefs and the lowest in mangroves (Supporting Information). Of the 36 observed species, 25 (69%) were identified with eDNA. This included species that were rarely observed (painted sweetlips [*Diagramma pictum*]) or were observed only in low numbers (e.g., orange‐spotted grouper [*Epinephelus coioides*]). At the genus level, 29 (88%) of the 33 genera observed were detected with eDNA (Fig. 4). An additional 2 of the visually observed genera were each detected in a single PCR replicate in the eDNA data, but were removed in the data filtering. Of the 49 genera recorded from trawling in the central Gulf in 1990, 32 (65%) were detected with eDNA; an additional 5 of these genera were detected in single PCR replicates (Fig. 4).

### Taxonomic Richness

Overall, coral reefs yielded the highest total species richness across both years, closely followed by the offshore sand bottom sites (Fig. 2 & Supporting Information). The samples from spring 2017 yielded a higher mean and total richness (across habitat types) than the summer 2016 samples (Fig. 2 & Supporting Information). The coral reef site at Halul Island (site CR_E) and the offshore sand bottom site SB.OUT_SE each yielded 83 detected taxa (mean across filter replicates of CR_E: 33 [SE 1] and 51 [5], SB.OUT_SE: 12 [1], and 50 [3] for 2016 and 2017, respectively), the highest richness obtained for any site (Supporting Information). The surface samples from Al Shaheen yielded more taxa than the deep‐water samples (2‐tailed *t* test with unequal variances, *p* = 0.002, mean of 19 and 10 taxa per filter, respectively). Forty taxa were found only in the surface samples, 11 were found only in the deep samples, and 52 were common to the 2 sampling depths (Supporting Information).

### Clustering and Ordination

**Figure 5:**
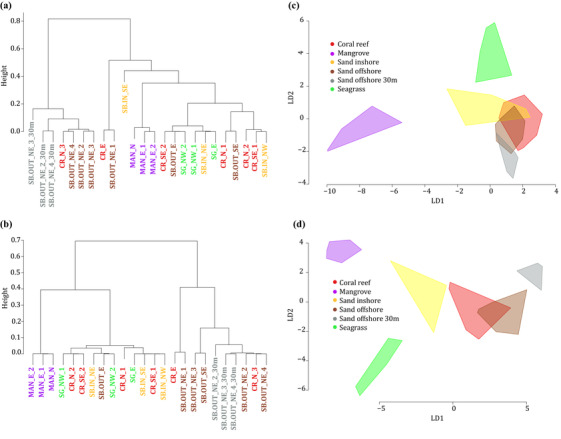
(a, b) Hierarchically clustering trees of eDNA sampling sites based on dissimilarity in the presence or absence of vertebrate taxa between sampling sites and (c, d) results of canonical analysis of principal coordinates ([a, c], 2016; [b, d], 2017). In (c) and (d), samples from the same habitat types are connected in polygons. On average across 4 rarefaction runs, the first 2 PCoA axes provide a mean cumulative classification success of 36% (SE 1) and 55% (1) of the observed variation, respectively, for 2016, and 51% (0) and 71% (0), respectively, for 2017 (rounded to nearest integer).

The median read depth per PCR replicate and thereby the rarefaction depth was 6647 and 4706 for the Riaz barcode (2016 and 2017, respectively) and 4903 and 28,028 reads for the MiFish barcode. Hierarchical clustering yielded similar results for both years (Fig. 5 & Supporting Information). The mangrove sites formed a well‐defined cluster, and the samples from Al Shaheen formed a cluster together with the nearest coral reef (CR_N_3) and often also with the offshore sand bottom site SB.OUT_SE. The highly diverse coral reef at Halul Island was clearly separated from the remaining reefs, clustering alone or together with the sand bottom site SB.OUT_NE_1. The seagrass and inshore sand bottom sites most often formed a mixed cluster with the remaining coral reef and offshore sand bottom sites (Fig. 5 & Supporting Information). Clustering based on read abundance yielded broadly similar results to presence‐absence‐based clustering, except that the reef at Halul Island was not clearly distinct from the remaining reefs and the seagrass sites consistently formed their own cluster in 2017 (Supporting Information). The average Raup–Crick dissimilarities between habitat types (presence‐absence data) also indicated some overlaps between habitat types, but dissimilarity was generally low within and higher between habitats (Supporting Information). The CAP analysis showed a clear separation between habitat types in 2017; only coral reefs and the surface samples from the northeastern sand bottom sites overlapped. For 2016, these 2 habitat types also overlapped with inshore sand bottom sites, and the surface samples from the northeastern sand bottom sites showed some overlap with the deep samples (Fig. 5). The first 2 PCoA axes provided a cumulative classification success of 36% (SE 1) and 55% (1), respectively, for 2016 and 51% (0) and 71% (0) respectively, for 2017 (across 4 rarefaction runs). The PERMANOVA test indicated that 61% (0) and 65% (0) (*p* = 0.001) of the variation in similarity between sampling sites could be explained by habitat type for both 2016 and 2017.

### Coverage of Diversity

**Figure 6:**
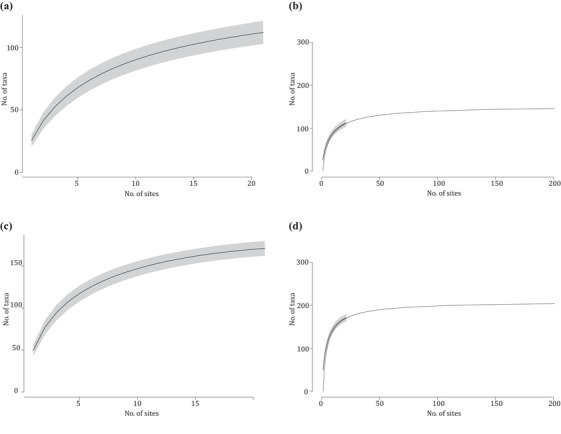
Species accumulation detected via eDNA in the Arabian Gulf across the 21 sampling sites (a) in 2016, (b) in 2016 with extrapolation of the fitted Lomolino model to 200 sampling sites, (c) in 2017, and (d) in 2017 with extrapolation of the fitted Lomolino model to 200 sampling sites (gray shading, 95% CIs based on unconditional SD). Samples from 30 m depth are excluded.

Species accumulation curves calculated across all sampling sites appeared to approach saturation, suggesting that most of the detectable vertebrate diversity had been covered (Fig. 6). Based on the Lomolino model, which provided the best fit to the data, total richness of the study area was estimated at 154 and 210 vertebrate taxa for 2016 and 2017, respectively. The total detected diversity across all 21 study sites (i.e., 112 and 171 taxa for 2016 and 2017, respectively; expected SD of 4 and 5, respectively, for this number of sites, based on species accumulation) corresponded to 73% and 81% of the estimated total richness of the sampled area. Extrapolation of the model (Figs. 6b & 6d) suggested that with 200 sampling sites, 96% (mean across both years) of the detectable diversity would be covered. Of the 443 bony fish species with confirmed presence in the Gulf (Carpenter et al. 2015), 66 species (15%) were detected with eDNA in this study. At the genus level, 95 (36%) of the 266 confirmed genera of bony fishes were detected.

If sequences were required to be present in at least 3 out of 4 PCR replicates to be retained in a sample, estimated total richness became 138 and 219 taxa, respectively, of which 56% and 54% were detected and of which 89% (mean across both years) was estimated to be detected with 200 sampling sites. However, this filtering approach removed a large number of taxa known to be present in the Gulf.

## Discussion

### Detected Diversity

Based on eDNA samples, we detected a broad range of marine vertebrates, encompassing cartilaginous fishes, bony fishes, turtles, birds, and mammals. The detected taxa included species that are common and widespread in the Gulf and some rarely recorded species, such as sharptooth lemon shark (*Negaprion acutidens*) (Moore et al. 2012). Driftfishes (Nomeidae) have not to our knowledge been recorded in the Gulf, but may be present as they have been recorded in Oman (Al‐Jufaili et al. 2010). The detection of eDNA matching the deep‐sea shark genus *Deania* was very surprising, and should not be regarded as evidence of occurrence in Qatar until this is confirmed through other methods. All the remaining taxa in the final data were known from the Gulf, and results from visual census and previous trawling efforts yielded a relatively good overlap with eDNA results. The higher taxonomic richness in eDNA samples from 2017 compared with 2016 could be partly due to a higher sequencing depth for the MiFish barcode, but richness was also higher for the Riaz barcode, suggesting that vertebrate richness in the study area was highest in 2017.

### Differentiation of Habitat Types

Most habitat types could be separated in ordination analyses, and PERMANOVA results supported habitat type as an important explanatory factor for eDNA composition. Coral reefs overlapped with the surface samples from offshore sand bottom sites, in accordance with the detection of reef‐associated species at the sites in the Al Shaheen oil field. Mangrove was the most distinct habitat type, despite geographical proximity to some coral reef and sand bottom sites. This is probably connected to the extremely high temperatures and salinity in the mangroves (Bangsgaard et al. 2012), but may also partly be explained by more limited transport of eDNA in and out of mangroves. Because eDNA transport may differ according to habitat type and time of year, future research should assess this process for different combinations of habitats and seasons. Clustering based on read abundances appeared to perform slightly better than presence‐absence‐based clustering in terms of grouping sites of the same habitat type. Although the interpretation of eDNA read abundances is still somewhat unclear, incorporating this quantitative information may aid for instance in decreasing the influence of false‐positive detections due to, for example, transport of eDNA.

Overall, the detected species compositions at different habitat types corresponded well with the species’ ecology. The fish species found only at coral reef sites (Supporting Information) are reef associated (Buchanan et al. 2016). The sand bottom sites at Al Shaheen yielded benthopelagic or demersal species and several reef‐associated species. This is not unexpected given the proximity (approximately 500 m) of these sand bottom sites to oil platforms, which provide attachment for corals and attract a large diversity of fish species (Torquato et al. 2017). However, some reef species (e.g., Chaetodontids) observed in abundance at the platforms were missing in the water samples, suggesting a relatively limited influence from the artificial reefs. Degradation of eDNA in seawater is generally rapid (Thomsen et al. 2012; Sigsgaard et al. 2016) and dependent on temperature (Tsuji et al. 2017). Given the high water temperatures in our study area combined with dilution, we expected limited large‐scale transport of eDNA. The fact that many of the species detected at the surface were not detected at 30 m depth also suggests that eDNA from the surface was not sinking to the deeper sampling depth. This could be because the eDNA was degraded or transported elsewhere before it could reach 30 m or because of the thermocline (about 18 m in summer [Reynolds 1993]), which could limit vertical eDNA transport.

### Monitoring of Low‐Density, Mobile Organisms

Dugong (*Dugong dugon*) eDNA was detected at seagrass and sand bottom sites, consistent with the species’ known habitat (Preen 2004). Like other low‐density, mobile marine organisms, dugongs are challenging to monitor, requiring difficult and costly surveying, such as dedicated aerial surveys. Thus, eDNA offers an important potential for more resource‐efficient monitoring of the dugong and similar species, such as manatees (Hunter et al. 2018). Sea snakes are also inconspicuous for visual recording (Bishop & Alsaffar 2008), making eDNA detection a promising alternative, although lower rates of activity and lack of continual skin shedding compared with, for example, fishes may lower the efficiency of the method for snakes (Hunter et al. 2015). Neither the Riaz or the MiFish primers seem well suited to amplify sea snake DNA based on sequences from annulated sea snake (*Hydrophis cyanocinctus*) and short sea snake (*Lapemis curtus*), which may explain why sea snakes were not detected in this study.

### Filtering and Curation of eDNA Sequences

False‐positive results are a serious pitfall for eDNA studies, which can arise and should be addressed at all stages of a metabarcoding study (Thomsen & Willerslev 2015; Zinger et al. 2019b). Our final eDNA data contained sequences from several domesticated animals and 3 non‐native fishes. Because these taxa were either common eDNA contaminants or had been the subject of previous work in our laboratory, they were deemed highly likely to be laboratory contaminants and were removed from further analyses. To limit such contaminations, we recommend that when possible, eDNA samples be extracted in a separate room from tissue samples. Several sequences were more abundant in field controls than in the eDNA samples and were therefore removed from further analyses (Taberlet et al. 2018). These sequences stemmed mainly from a control that was processed in a fishing harbor, and it is thus likely that the contaminations stemmed from nearby fish catches, perhaps via aerosol transport of DNA. This result underlines that field controls should be collected in the exact same sampling environment as the eDNA samples.

We used 2 metabarcodes, both targeting the mitochondrial 12S gene. The relatively short Riaz barcode sometimes produced 100% matches to several related species, precluding species‐level identification without applying previous knowledge of species distributions. The greater intraspecific variation in the MiFish barcode often lead to a match of only 99% to the only regionally recorded species within a detected genus. Manual curation of eDNA sequence matches to a reference database is currently unavoidable if one wants to reliably identify eDNA sequences, and even then many sequences can only be identified to family or genus level. This is mostly due to incompleteness of reference databases and will thus become less essential as databases are expanded. Importantly, many ecological analyses can be done without or with only higher level taxonomic identification of eDNA sequences if proper reference sequences are unavailable (e.g., Zinger et al. 2019a).

### Coverage of Biodiversity

Extrapolation of species accumulation curves suggested that >90% of the detectable diversity could be recovered with 200 sampling sites. This result is similar to the couple of hundred 2 × 2 L seawater samples Boussarie et al. (2018) used to estimate shark diversity in New Caledonia. Of course, for a given area, covering vertebrate diversity will be expected to require a greater sampling effort than covering shark diversity alone. Also, some vertebrates, such as sea snakes, may not have been detectable with our approach, and (in addition to, e.g., improving PCR primers) a greater sampling effort may be required to include these taxa.

Covering more habitats, depths, and seasonal variation may be equally or more efficient than simply increasing the sample size. In addition to expanding the spatial and temporal coverage of monitoring, habitat differentiation based on eDNA should be validated for a broader range of taxa, including the dominant habitat‐forming species. Finally, the approach should be tested in more biodiverse areas, where a greater sampling effort may be needed, and in areas with different environmental conditions, such as low temperatures leading to slower eDNA degradation, and thereby perhaps a lower spatial resolution.

Our results demonstrate that marine vertebrate communities associated with different habitat types can be distinguished and efficiently characterized across a large spatial scale with eDNA from seawater samples. Environmental DNA could thus potentially be used for national and regional biomonitoring of marine habitats (e.g., for environmental impact assessments). This would be especially useful in areas of high conservation priority and areas that are difficult or costly to survey with conventional methods and would constitute an important step toward more comprehensive, holistic ecosystem assessments.

## Supporting information

Plots of species richness (Appendix S1), map of eDNA‐based taxon richness at the study sites (Appendix S2), MOTUs as a function of read depth (Appendix S3), hierarchical clustering (Appendix S4), comparison of taxa detected with eDNA at the surface and at 30 m (Appendix S5), read count as a function of initial DNA concentration for the mock sample (Appendix S6), sequence similarity between taxa in applied metabarcodes (Appendix S7), species accumulation curves for individual eDNA samples (Appendix S8) and individual sampling sites (Appendix S9), sampling site information (Appendix S10), results of visual census (Appendix S11), overview of vertebrate taxa detected (Appendix S12), taxon lists for each habitat type (Appendix S13), Raup–Crick dissimilarities between and within habitats (Appendix S14), taxonomic composition of mock sample (Appendix S15), overview of taxa filtered from eDNA data (Appendix S16), and details on procurement of additional reference sequences, PCR amplification, library building, sequencing, and trawling in 1990 (Appendix S17) are available online. The authors are solely responsible for the content and functionality of these materials. Queries (other than absence of the material) should be directed to the corresponding author. All eDNA sequencing data, associated data on tags. The sequencing libraries were demultiplexed by index by the sequencing facility, so only the tags are needed/relevant to analyse the data and the amplicon sequence variant (ASV) tables obtained with DADA2 are available on the Dryad Data Repository (https://doi.org/10.5061/dryad.hmgqnk9c0). The sequences derived from tissue samples are available in the GenBank database under accession numbers MH248164‐MH248256.
