# Supplementary material for: Using vertebrate environmental DNA from seawater in biomonitoring of marine habitats
Source: Conserv Biol. 2019 Dec 24;34(3):697–710. doi: 10.1111/cobi.13437 (PMC7318234; doi:10.1111/cobi.13437)
Supplement: Supplementary file 1 — Plots of species richness (Appendix S1), map of eDNA‐based taxon richness at the study sites (Appendix S2), MOTUs as a function of read depth (Appendix S3), hierarchical clustering (Appendix S4), comparison of taxa detected with eDNA at the surface and at 30 m (Appendix S5), read count as a function of initial DNA concentration for the mock sample (Appendix S6), sequence similarity between taxa in applied metabarcodes (Appendix S7), species accumulation curves for individual eDNA samples (Appendix S8) and individual sampling sites (Appendix S9), sampling site information (Appendix S10), results of visual census (Appendix S11), overview of vertebrate taxa detected (Appendix S12), taxon lists for each habitat type (Appendix S13), Raup–Crick dissimilarities between and within habitats (Appendix S14), taxonomic composition of mock sample (Appendix S15), overview of taxa filtered from eDNA data (Appendix S16), and details on procurement of additional reference sequences, PCR amplification, library building, sequencing, and trawling in 1990 (Appendix S17) are available online. The authors are solely responsible for the content and functionality of these materials. Queries (other than absence of the material) should be directed to the corresponding author. All eDNA sequencing data, associated data on tags. The sequencing libraries were demultiplexed by index by the sequencing facility, so only the tags are needed/relevant to analyse the data and the amplicon sequence variant (ASV) tables obtained with DADA2 are available on the Dryad Data Repository (https://doi.org/10.5061/dryad.hmgqnk9c0). The sequences derived from tissue samples are available in the GenBank database under accession numbers MH248164‐MH248256. [file COBI-34-697-s001.pdf]

Supporting Information for:

"Using vertebrate environmental DNA from seawater in biomonitoring of marine habitats"

Eva Egelyng Sigsgaard[1*†], Felipe Torquato[1], Tobias Guldberg Frøslev[2], Alec B. M. Moore[3], Johan Mølgård Sørensen[1], Pedro Range[4], Radhouane Ben-Hamadou[5], Steffen Sanvig Bach[6**], Peter Rask Møller[1], Philip Francis Thomsen[2†]

[1] Natural History Museum of Denmark, University of Copenhagen, Universitetsparken 15, DK-2100 Copenhagen Ø, Denmark.

[2] Section for GeoGenetics, Globe Institute, University of Copenhagen, Øster Voldgade 5-7, DK-1350 Copenhagen K, Denmark (previously: Centre for GeoGenetics, Natural History Museum of Denmark).

[3] School of Ocean Sciences, Bangor University, Menai Bridge, Anglesey, LL59 5AB, U.K.

[4] Environmental Science Center, Qatar University, P.O. Box: 2713, Doha, Qatar.

[5] Department of Biological and Environmental Sciences, Qatar University, P.O. Box: 2713, Doha, Qatar.

[6] Maersk Oil Research and Technology Centre, Al Jazi Tower, Building 20, Zone 60, Street 850, West Bay, Doha, Qatar.

*email: eva.sigsgaard@bios.au.dk, pfthomsen@bios.au.dk

†Current address: Department of Bioscience, University of Aarhus, Ny Munkegade 116, Building 1540, 8000 Aarhus C Denmark

**Current address: Rambøll, Hannemanns Allé 53, DK-2300 Copenhagen S, Denmark

Table of contents

Supplementary Figures

Appendix 1……………………………………………………………………………3
Appendix 2……………………………………………………………………………4
Appendix 3……………………………………………………………………………4
Appendix 4……………………………………………………………………………5
Appendix 5…………………………………………………………………………...13
Appendix 6…………………………………………………………………………...14
Appendix 7…………………………………………………………………………...15
Appendix 8…………………………………………………………………………...16
Appendix 9…………………………………………………………………………...24

Supplementary Tables

Appendix 10…………………………………………………………………………….26
Appendix 11…………………………………………………………………………….27
Appendix 12…………………………………………………………………………….30
Appendix 13…………………………………………………………………………….43
Appendix 14…………………………………………………………………………….48
Appendix 15…………………………………………………………………………….49
Appendix 16…………………………………………………………………………….50

Supplementary Experimental Procedures

Appendix 17……………………….………………………………………………...54

Supplementary Figures

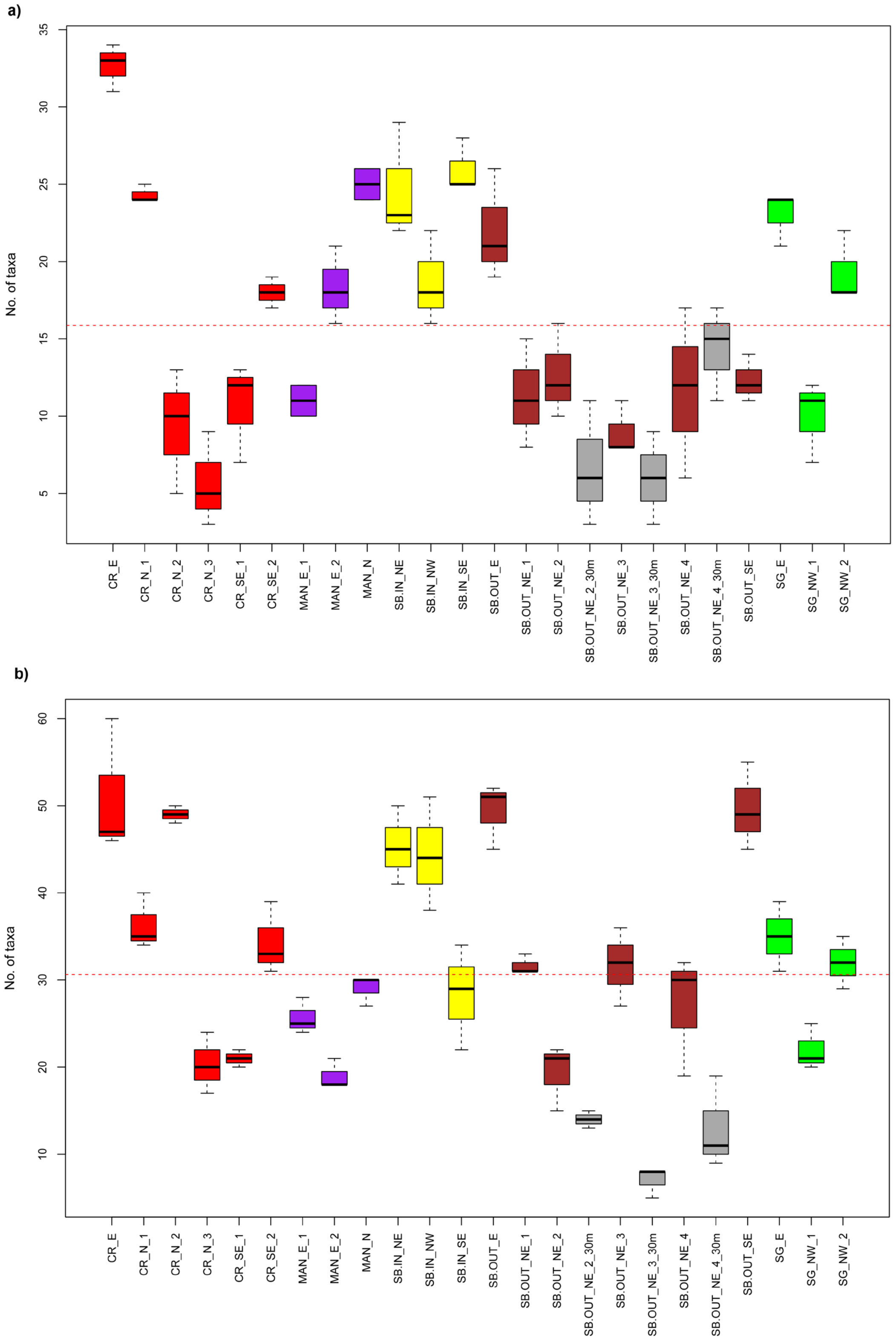

Appendix 1 Boxplot of eDNA-based species richness across the three filter replicates from each site. a) 2016; b) 2017. CR: Coral reef; MAN: Mangrove; SB_IN: Sand bottom inshore; SB_OUT: Sand bottom offshore; SG: Seagrass.

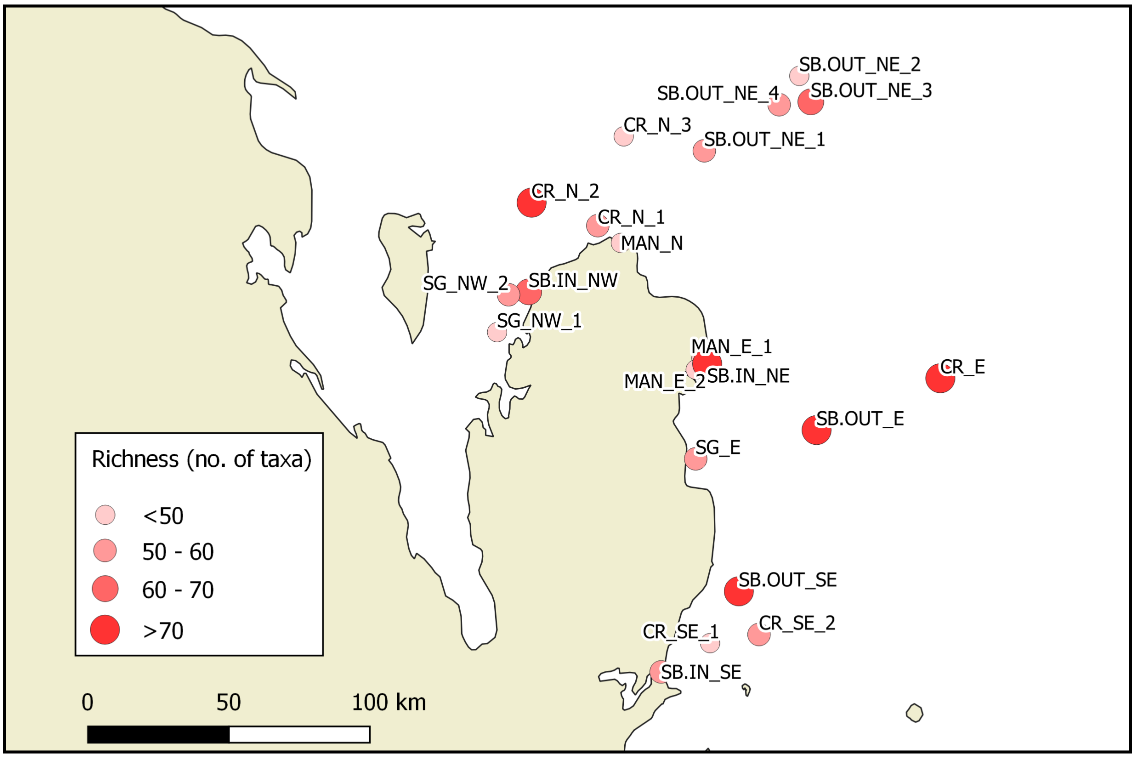

Appendix 2 Map of total eDNA-based taxon richness at the sampling sites across both sampling years. CR: Coral reef; MAN: Mangrove; SB_IN: Sand bottom inshore; SB_OUT: Sand bottom offshore; SG: Seagrass.

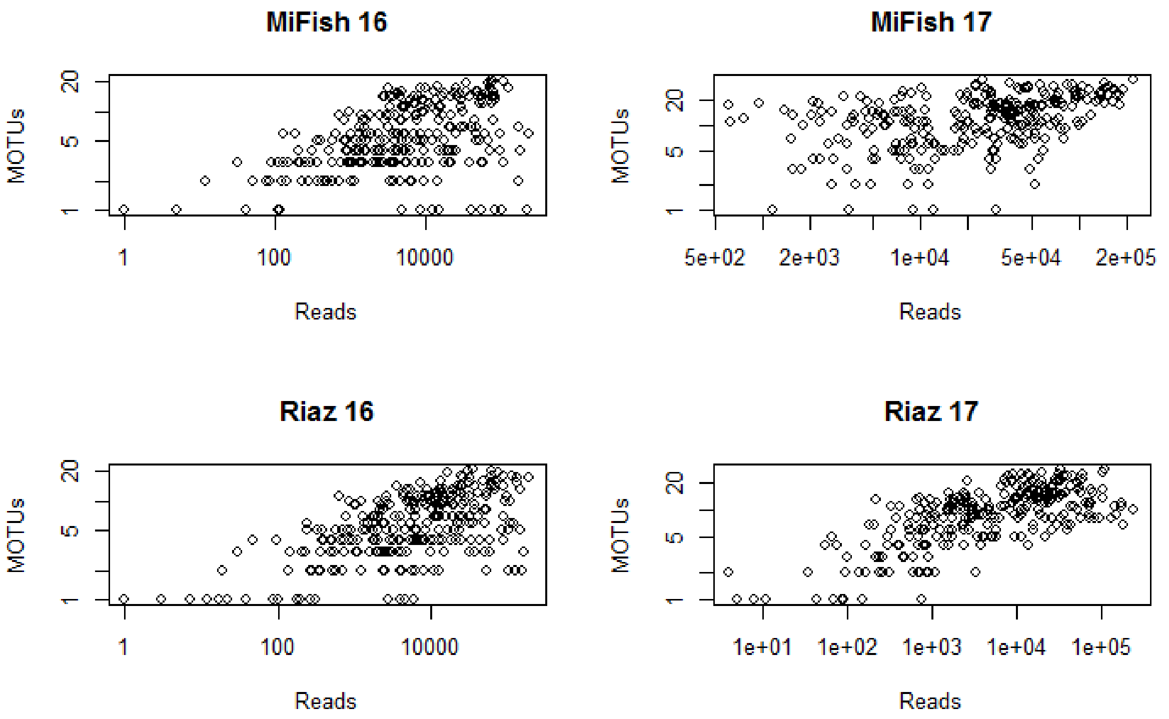

Appendix 3 Scatter plot of MOTUs as a function of read count for the PCR replicates of each dataset, before data filtering in R.

a)

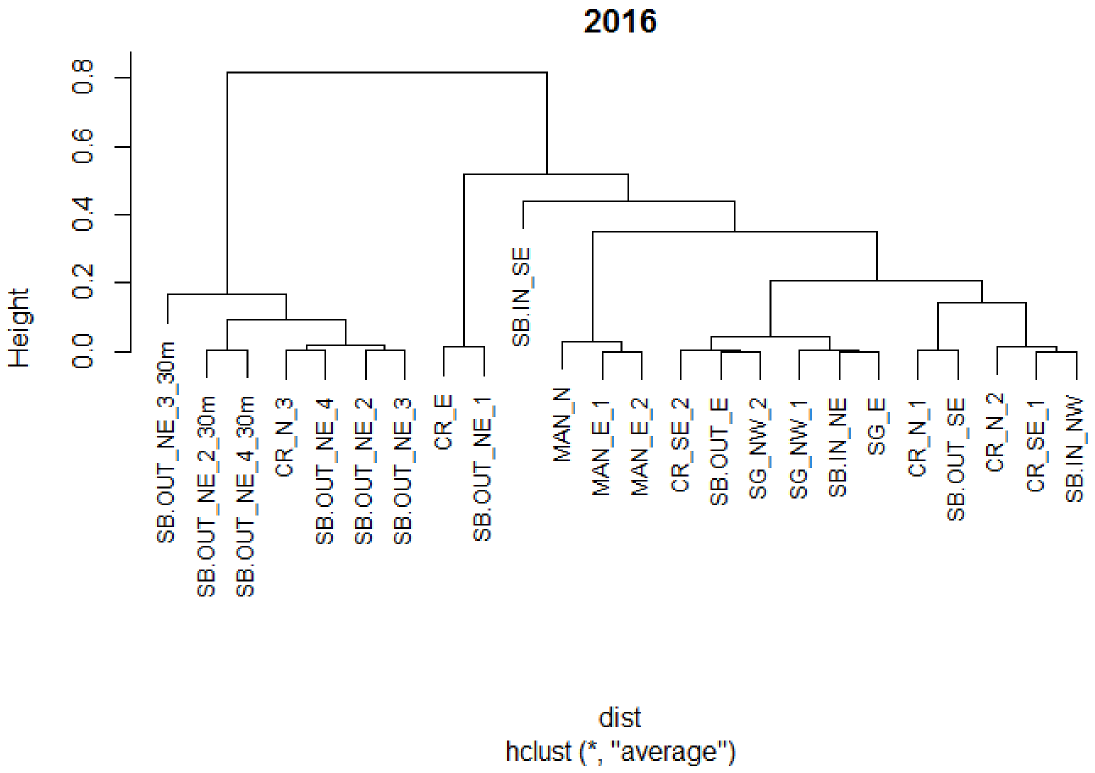
2016
Height
0.0
0.2
0.4
0.6
0.8
SB.OUT_NE_3_30m
SB.OUT_NE_2_30m
SB.OUT_NE_4_30m
CR_N_3
SB.OUT_NE_4
SB.OUT_NE_2
SB.OUT_NE_3
CR_E
SB.OUT_NE_1
SB.IN_SE
MAN_N
MAN_E_1
MAN_E_2
CR_SE_2
SB.OUT_E
SG_NW_2
SG_NW_1
SB.IN_NE
SG_E
CR_N_1
SB.OUT_SE
CR_N_2
CR_SE_1
SB.IN_NW
dist
hclust (*, "average")

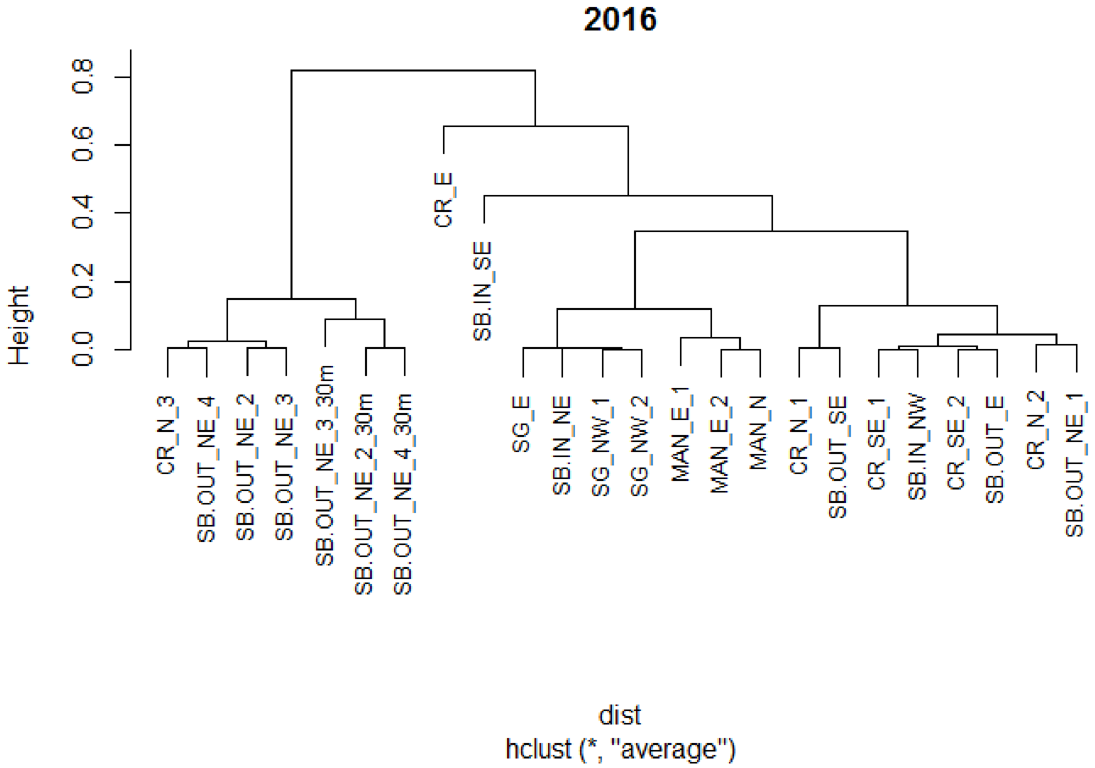
2016
Height
0.0
0.2
0.4
0.6
0.8
CR_N_3
SB.OUT_NE_4
SB.OUT_NE_2
SB.OUT_NE_3
SB.OUT_NE_3_30m
SB.OUT_NE_2_30m
SB.OUT_NE_4_30m
CR_E
SB.IN_SE
SG_E
SB.IN_NE
SG_NW_1
SG_NW_2
MAN_E_1
MAN_E_2
MAN_N
CR_N_1
SB.OUT_SE
CR_SE_1
SB.IN_NW
CR_SE_2
SB.OUT_E
CR_N_2
SB.OUT_NE_1
dist
hclust (*, "average")

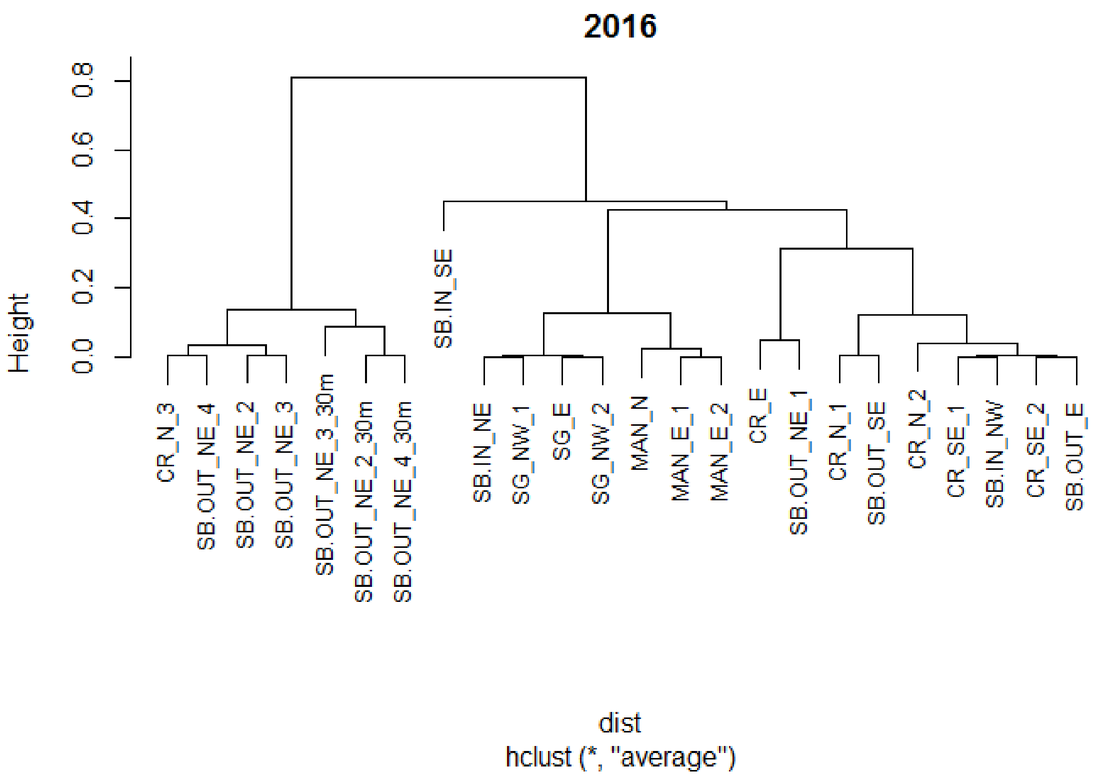
2016
Height
0.0
0.2
0.4
0.6
0.8
CR_N_3
SB.OUT_NE_4
SB.OUT_NE_2
SB.OUT_NE_3
SB.OUT_NE_3_30m
SB.OUT_NE_2_30m
SB.OUT_NE_4_30m
SB.IN_SE
SB.IN_NE
SG_NW_1
SG_E
SG_NW_2
MAN_N
MAN_E_1
MAN_E_2
CR_E
SB.OUT_NE_1
CR_N_1
SB.OUT_SE
CR_N_2
CR_SE_1
SB.IN_NW
CR_SE_2
SB.OUT_E
dist
hclust (*, "average")

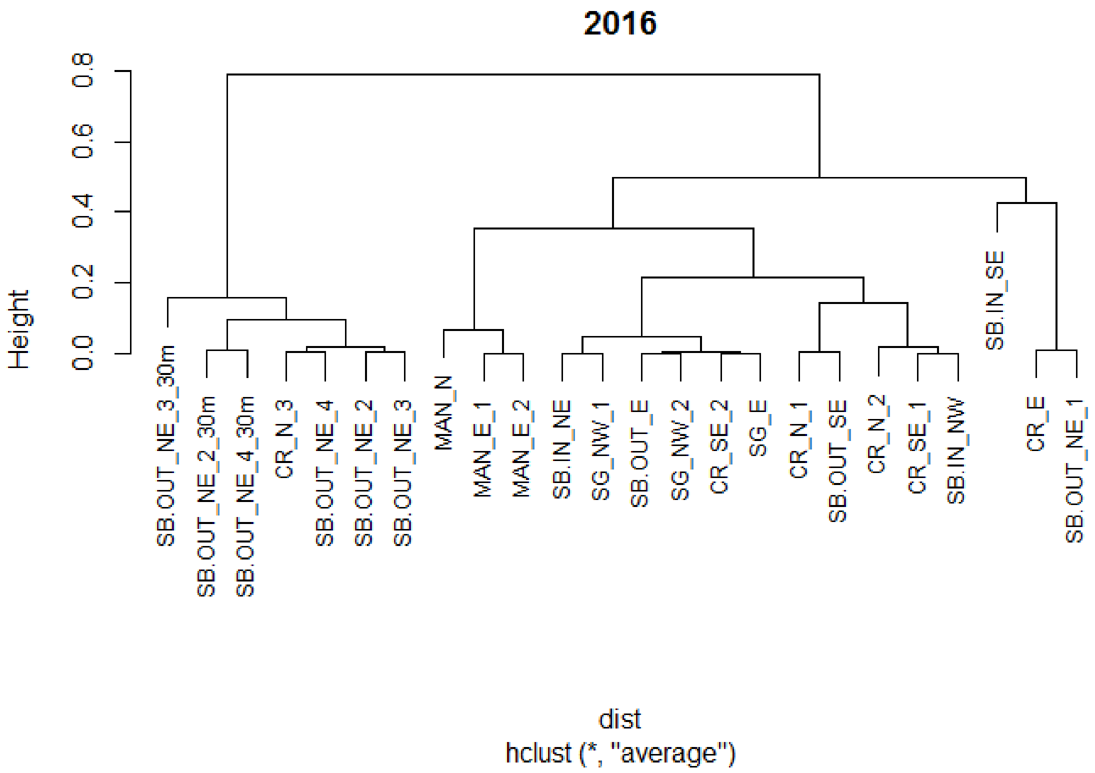
2016
Height
0.0
0.2
0.4
0.6
0.8
SB.OUT_NE_3_30m
SB.OUT_NE_2_30m
SB.OUT_NE_4_30m
CR_N_3
SB.OUT_NE_4
SB.OUT_NE_2
SB.OUT_NE_3
MAN_N
MAN_E_1
MAN_E_2
SB.IN_NE
SG_NW_1
SB.OUT_E
SG_NW_2
CR_SE_2
SG_E
CR_N_1
SB.OUT_SE
CR_N_2
CR_SE_1
SB.IN_NW
SB.IN_SE
CR_E
SB.OUT_NE_1
dist
hclust (*, "average")

b)

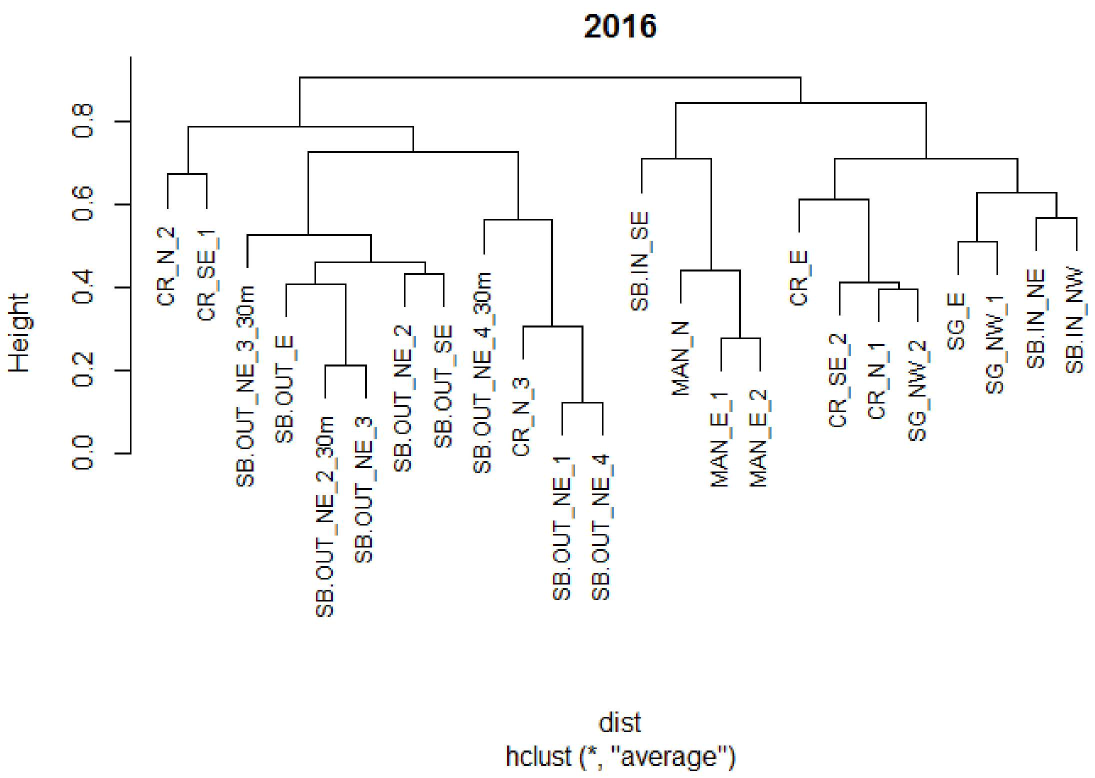
2016
Height
0.0
0.2
0.4
0.6
0.8
CR_N_2
CR_SE_1
SB.OUT_NE_3_30m
SB.OUT_E
SB.OUT_NE_2_30m
SB.OUT_NE_3
SB.OUT_NE_2
SB.OUT_SE
SB.OUT_NE_4_30m
CR_N_3
SB.OUT_NE_1
SB.OUT_NE_4
SB.IN_SE
MAN_N
MAN_E_1
MAN_E_2
CR_E
CR_SE_2
CR_N_1
SG_NW_2
SG_E
SG_NW_1
SB.IN_NE
SB.IN_NW
dist
hclust (*, "average")

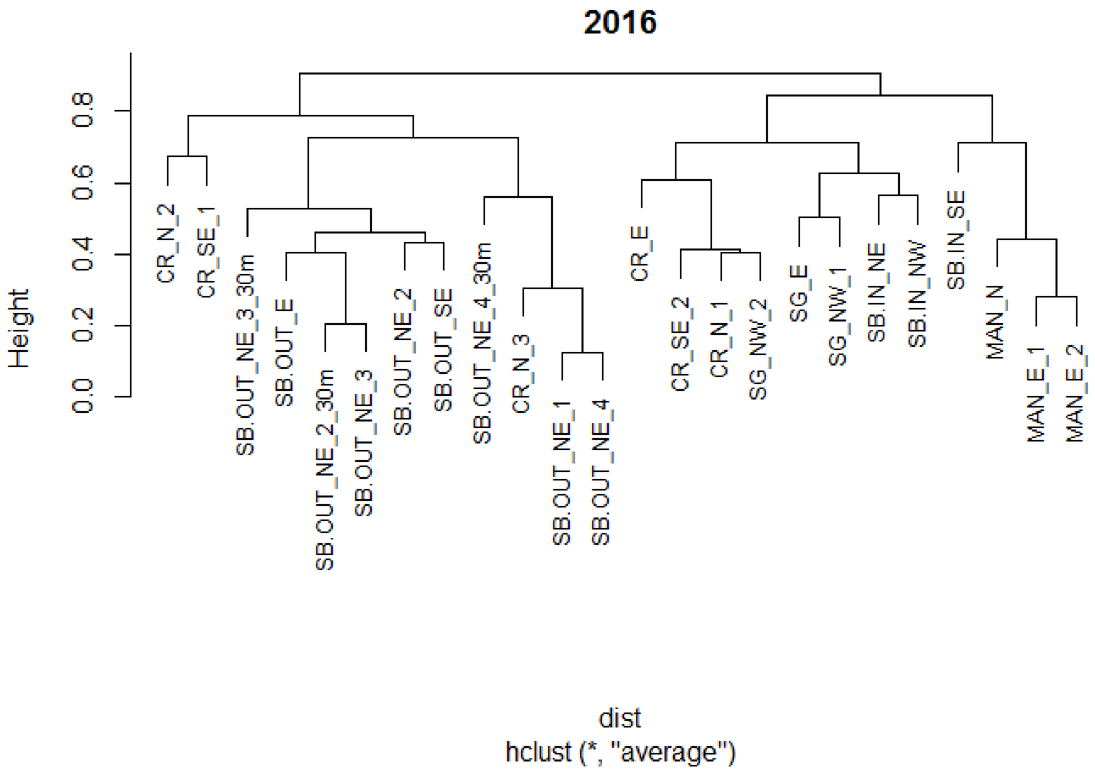
2016
Height
0.0
0.2
0.4
0.6
0.8
CR_N_2
CR_SE_1
SB.OUT_NE_3_30m
SB.OUT_E
SB.OUT_NE_2_30m
SB.OUT_NE_3
SB.OUT_NE_2
SB.OUT_SE
SB.OUT_NE_4_30m
CR_N_3
SB.OUT_NE_1
SB.OUT_NE_4
CR_E
CR_SE_2
CR_N_1
SG_NW_2
SG_E
SG_NW_1
SB.IN_NE
SB.IN_NW
SB.IN_SE
MAN_N
MAN_E_1
MAN_E_2
dist
hclust (*, "average")

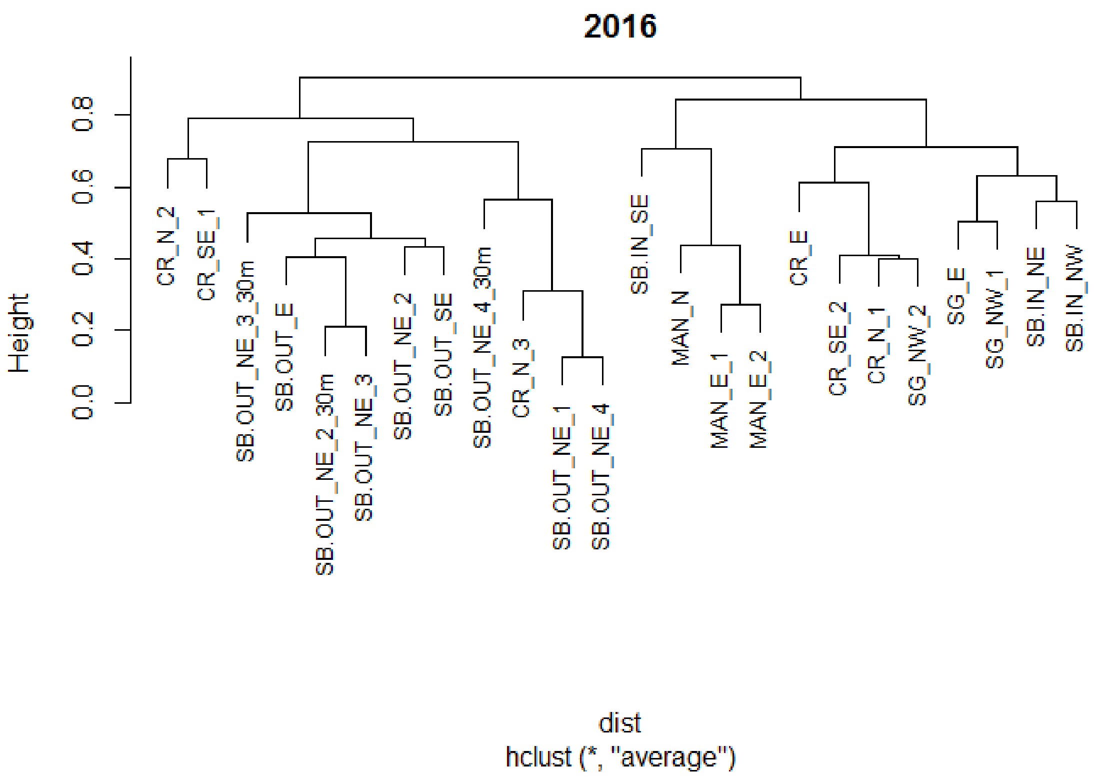
2016
Height
0.0
0.2
0.4
0.6
0.8
CR_N_2
CR_SE_1
SB.OUT_NE_3_30m
SB.OUT_E
SB.OUT_NE_2_30m
SB.OUT_NE_3
SB.OUT_NE_2
SB.OUT_SE
SB.OUT_NE_4_30m
CR_N_3
SB.OUT_NE_1
SB.OUT_NE_4
SB.IN_SE
MAN_N
MAN_E_1
MAN_E_2
CR_E
CR_SE_2
CR_N_1
SG_NW_2
SG_E
SG_NW_1
SB.IN_NE
SB.IN_NW
dist
hclust (*, "average")

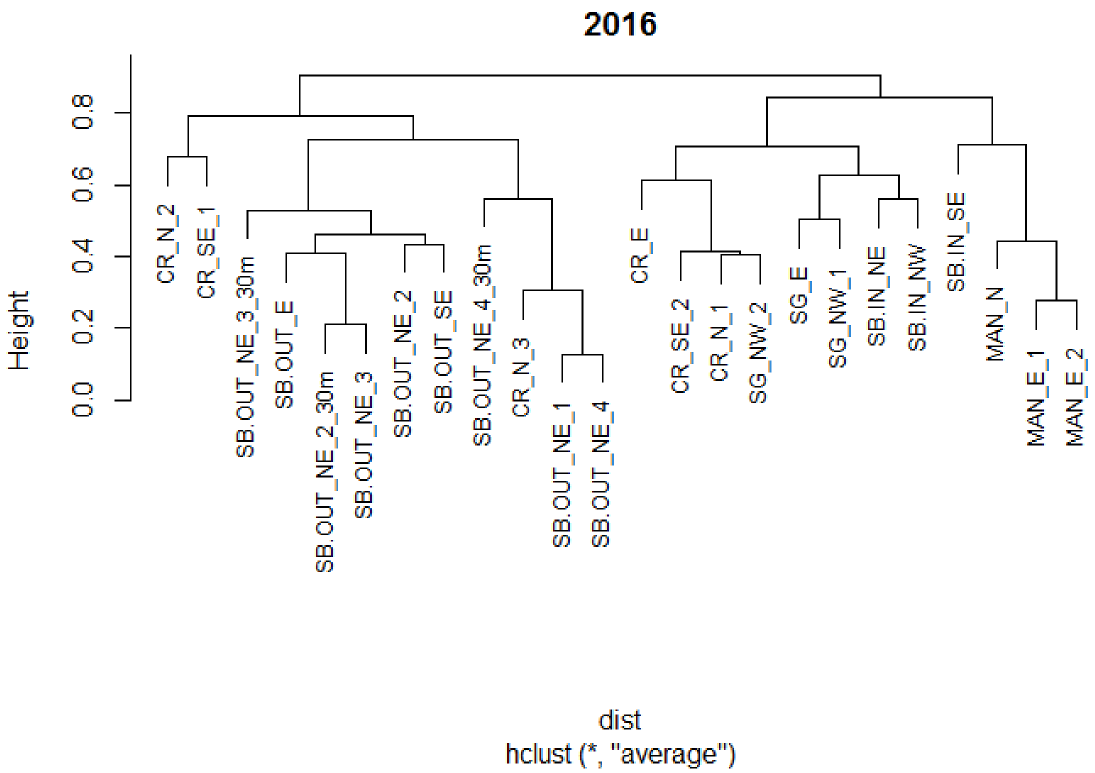
2016
Height
0.0
0.2
0.4
0.6
0.8
CR_N_2
CR_SE_1
SB.OUT_NE_3_30m
SB.OUT_E
SB.OUT_NE_2_30m
SB.OUT_NE_3
SB.OUT_NE_2
SB.OUT_SE
SB.OUT_NE_4_30m
CR_N_3
SB.OUT_NE_1
SB.OUT_NE_4
CR_E
CR_SE_2
CR_N_1
SG_NW_2
SG_E
SG_NW_1
SB.IN_NE
SB.IN_NW
SB.IN_SE
MAN_N
MAN_E_1
MAN_E_2
dist
hclust (*, "average")

c)

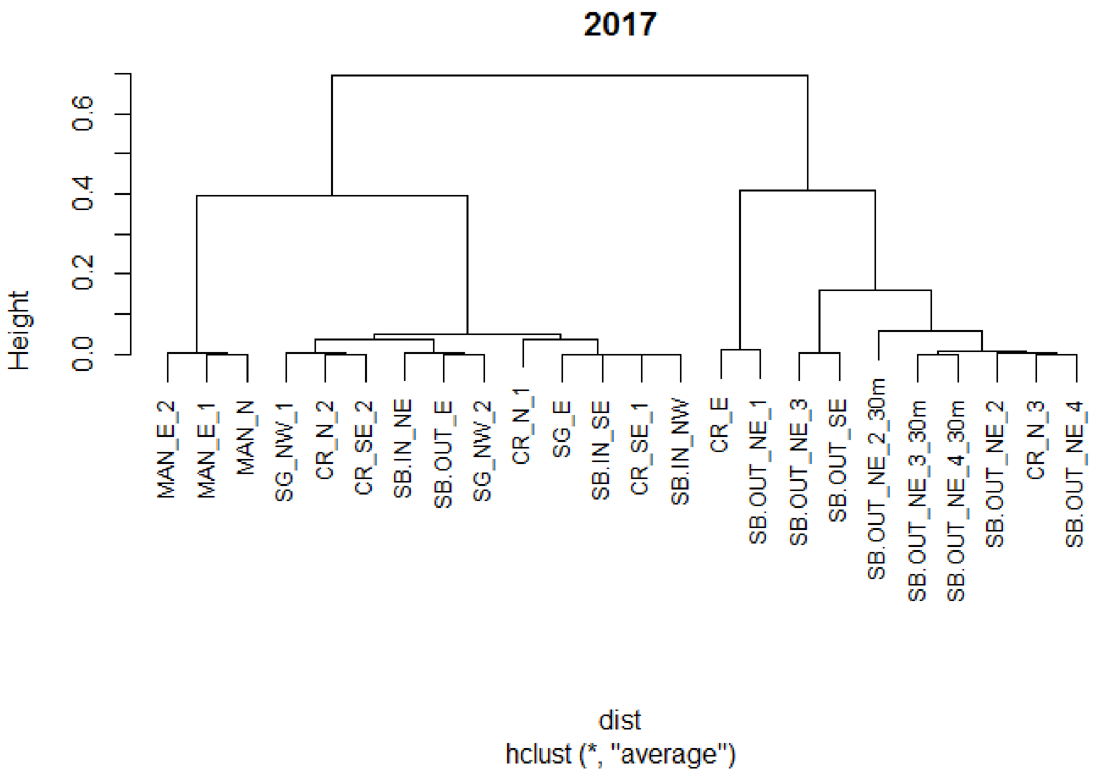
2017
Height
0.0
0.2
0.4
0.6
MAN_E_2
MAN_E_1
MAN_N
SG_NW_1
CR_N_2
CR_SE_2
SB.IN_NE
SB.OUT_E
SG_NW_2
CR_N_1
SG_E
SB.IN_SE
CR_SE_1
SB.IN_NW
CR_E
SB.OUT_NE_1
SB.OUT_NE_3
SB.OUT_SE
SB.OUT_NE_2_30m
SB.OUT_NE_3_30m
SB.OUT_NE_4_30m
SB.OUT_NE_2
CR_N_3
SB.OUT_NE_4
dist
hclust (*, "average")

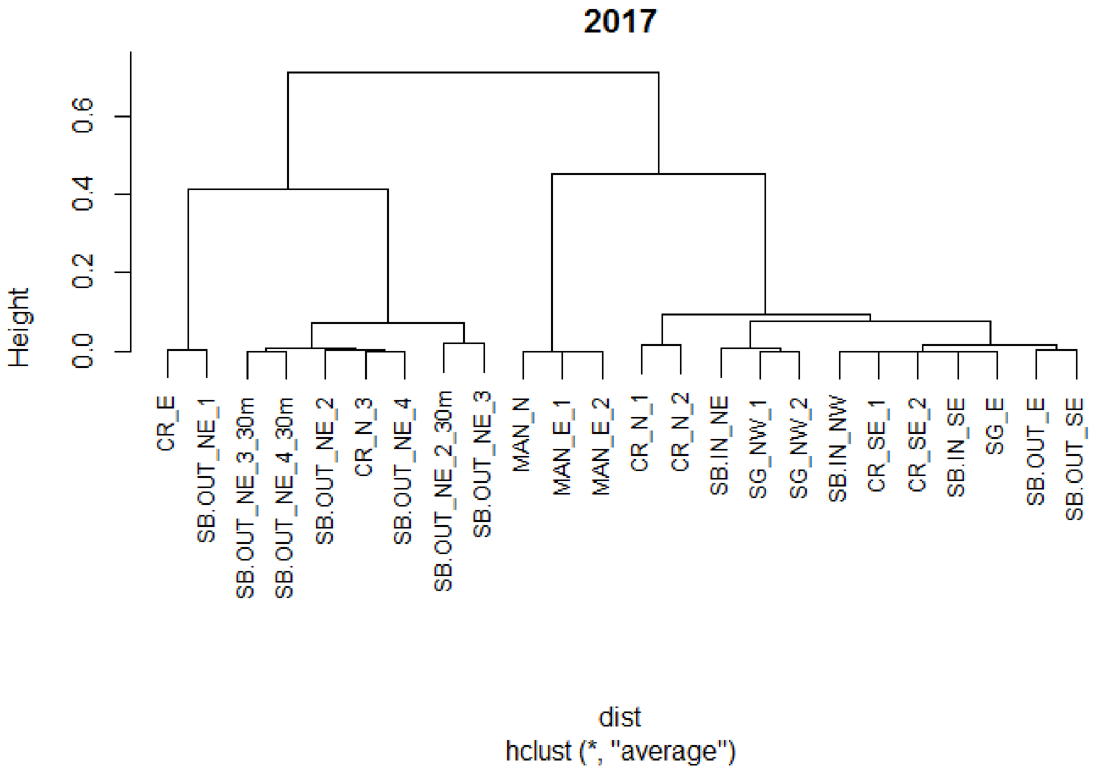
2017
Height
0.0
0.2
0.4
0.6
CR_E
SB.OUT_NE_1
SB.OUT_NE_3_30m
SB.OUT_NE_4_30m
SB.OUT_NE_2
CR_N_3
SB.OUT_NE_4
SB.OUT_NE_2_30m
SB.OUT_NE_3
MAN_N
MAN_E_1
MAN_E_2
CR_N_1
CR_N_2
SB.IN_NE
SG_NW_1
SG_NW_2
SB.IN_NW
CR_SE_1
CR_SE_2
SB.IN_SE
SG_E
SB.OUT_E
SB.OUT_SE
dist
hclust (*, "average")

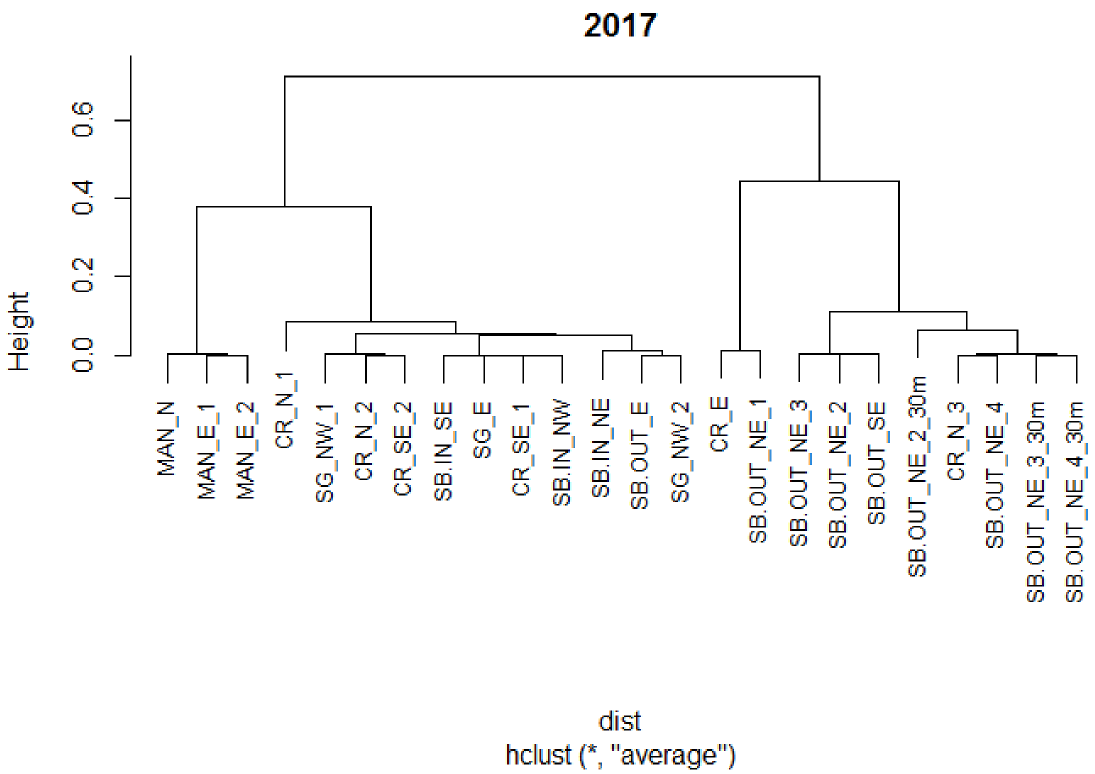
2017
Height
0.0
0.2
0.4
0.6
MAN_N
MAN_E_1
MAN_E_2
CR_N_1
SG_NW_1
CR_N_2
CR_SE_2
SB.IN_SE
SG_E
CR_SE_1
SB.IN_NW
SB.IN_NE
SB.OUT_E
SG_NW_2
CR_E
SB.OUT_NE_1
SB.OUT_NE_3
SB.OUT_NE_2
SB.OUT_SE
SB.OUT_NE_2_30m
CR_N_3
SB.OUT_NE_4
SB.OUT_NE_3_30m
SB.OUT_NE_4_30m
dist
hclust (*, "average")

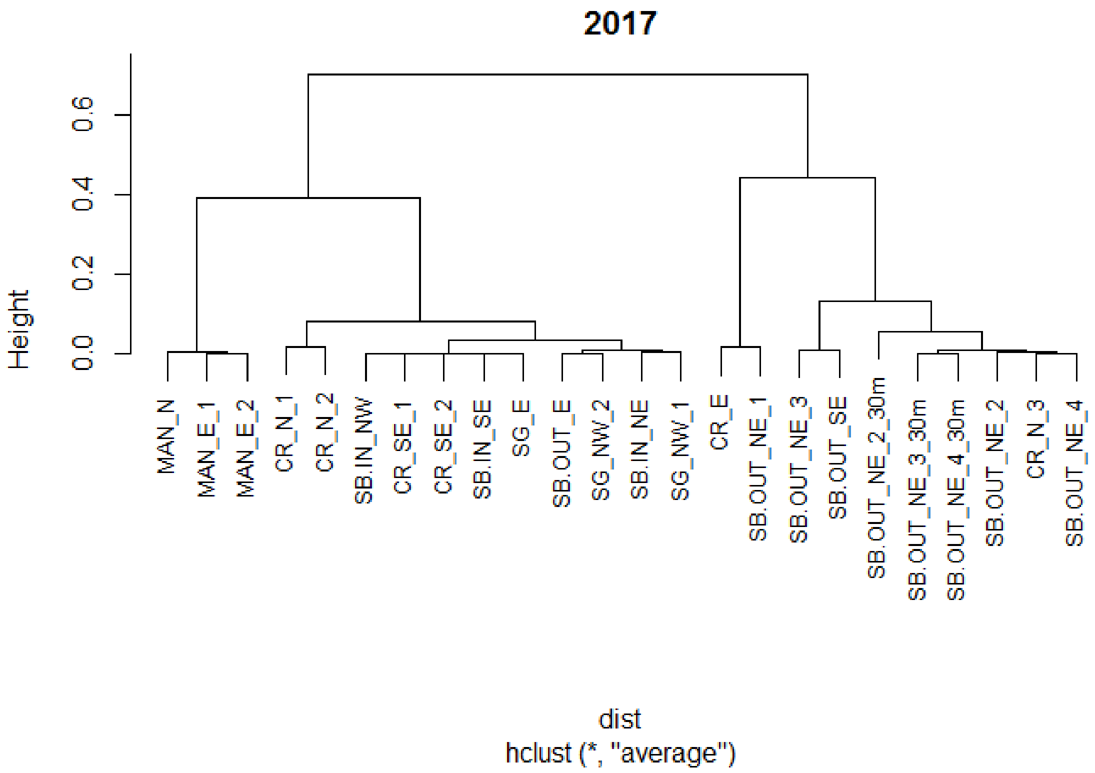
2017
Height
0.0
0.2
0.4
0.6
MAN_N
MAN_E_1
MAN_E_2
CR_N_1
CR_N_2
SB.IN_NW
CR_SE_1
CR_SE_2
SB.IN_SE
SG_E
SB.OUT_E
SG_NW_2
SB.IN_NE
SG_NW_1
CR_E
SB.OUT_NE_1
SB.OUT_NE_3
SB.OUT_SE
SB.OUT_NE_2_30m
SB.OUT_NE_3_30m
SB.OUT_NE_4_30m
SB.OUT_NE_2
CR_N_3
SB.OUT_NE_4
dist
hclust (*, "average")

d)

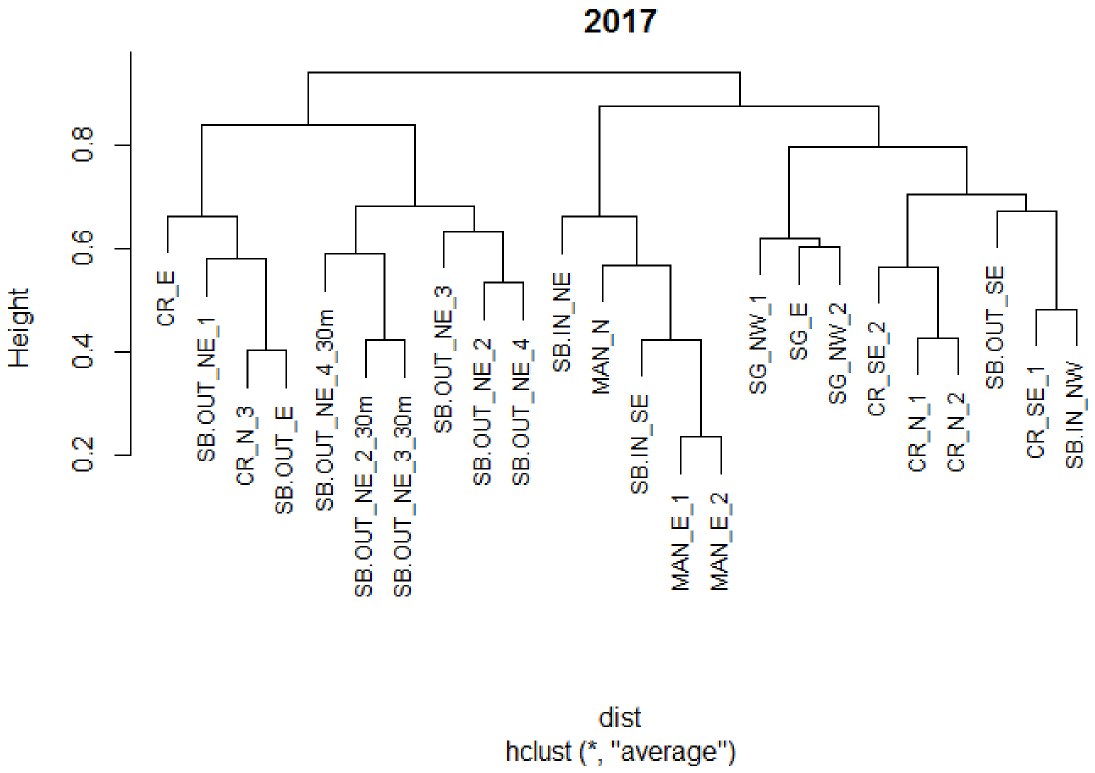
2017
Height
0.2
0.4
0.6
0.8
CR_E
SB.OUT_NE_1
CR_N_3
SB.OUT_E
SB.OUT_NE_4_30m
SB.OUT_NE_2_30m
SB.OUT_NE_3_30m
SB.OUT_NE_3
SB.OUT_NE_2
SB.OUT_NE_4
SB.IN_NE
MAN_N
SB.IN_SE
MAN_E_1
MAN_E_2
SG_NW_1
SG_E
SG_NW_2
CR_SE_2
CR_N_1
CR_N_2
SB.OUT_SE
CR_SE_1
SB.IN_NW
dist
hclust (*, "average")

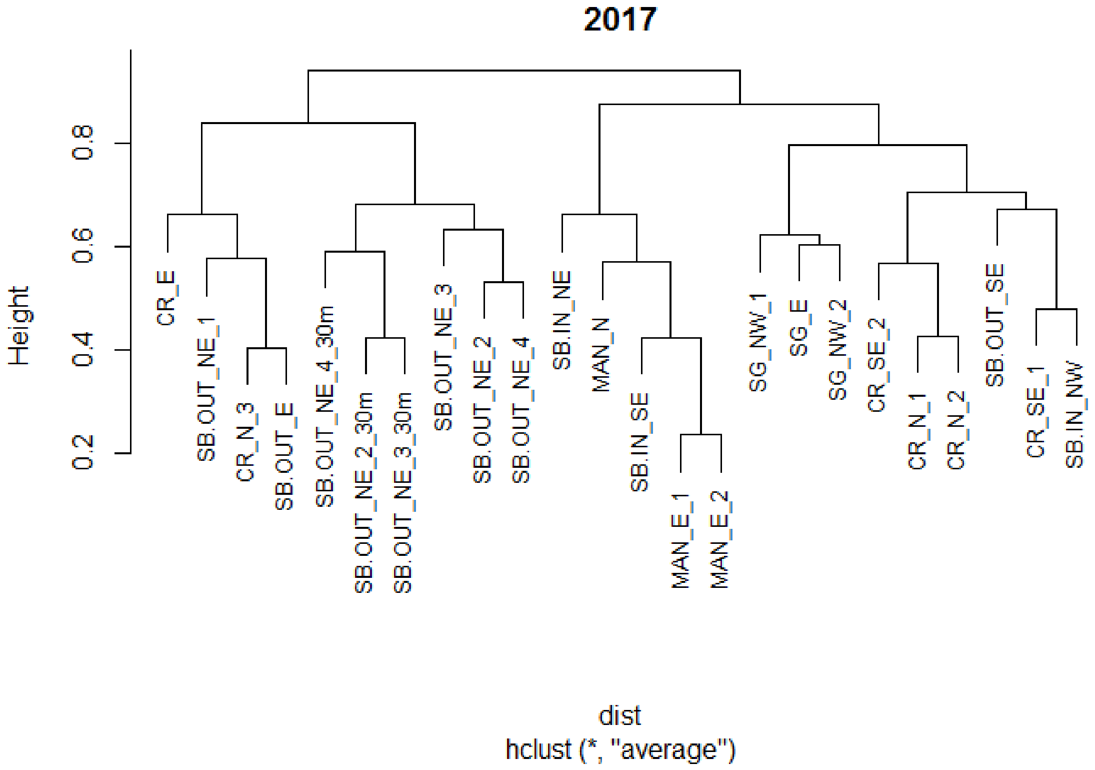
2017
Height
0.2
0.4
0.6
0.8
CR_E
SB.OUT_NE_1
CR_N_3
SB.OUT_E
SB.OUT_NE_4_30m
SB.OUT_NE_2_30m
SB.OUT_NE_3_30m
SB.OUT_NE_3
SB.OUT_NE_2
SB.OUT_NE_4
SB.IN_NE
MAN_N
SB.IN_SE
MAN_E_1
MAN_E_2
SG_NW_1
SG_E
SG_NW_2
CR_SE_2
CR_N_1
CR_N_2
SB.OUT_SE
CR_SE_1
SB.IN_NW
dist
hclust (*, "average")

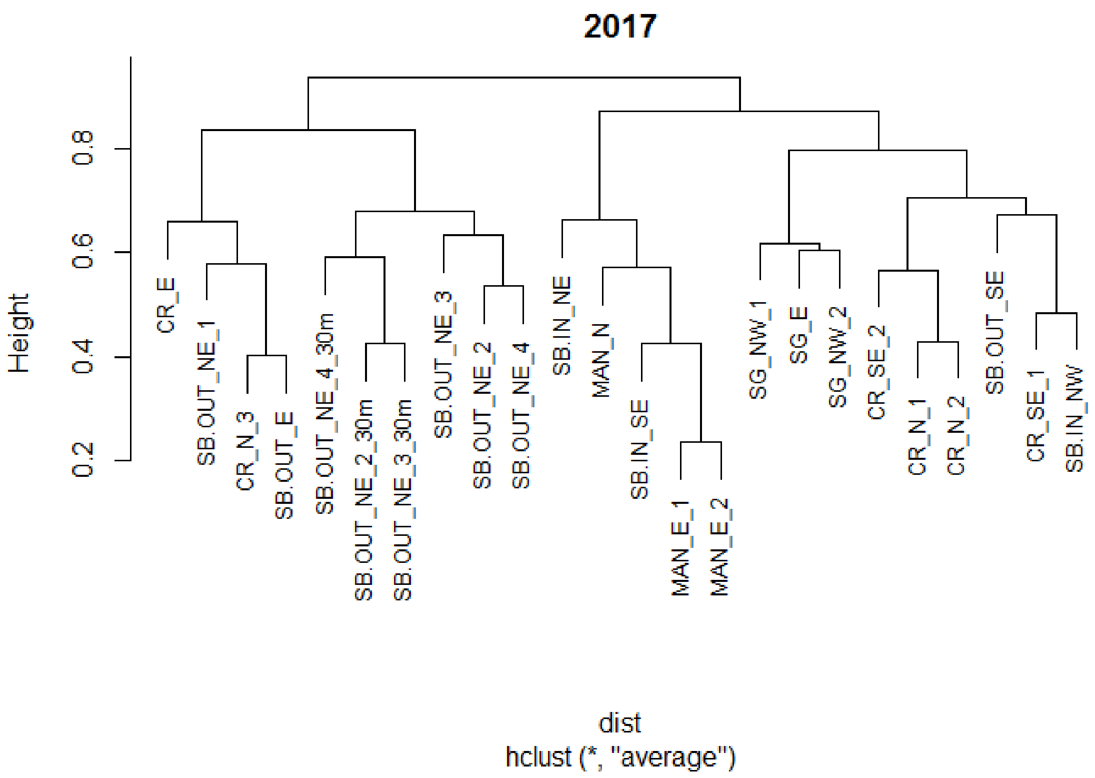

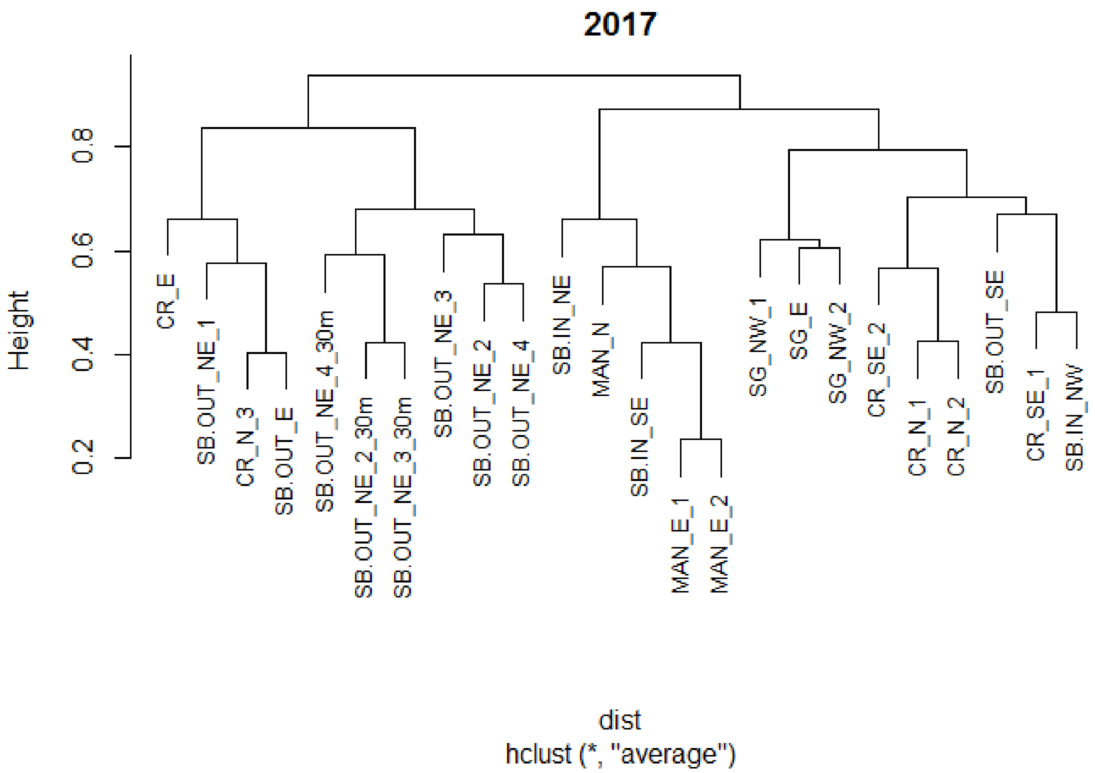

Appendix 4. Cluster plots from four different runs of rarefaction (re-sampling) of the eDNA data. Only unique configurations are shown for each of; a) 2016 data and Raup-Crick index using presence-absence; b) 2016 data and Bray-Curtis index using read abundance; c) 2017 data and Raup-Crick index using presence-absence; d) 2017 data and Bray-Curtis index using read abundance. CR: Coral reef; MAN: Mangrove; SB_IN: Sand bottom inshore; SB_OUT: Sand bottom offshore; SG: Seagrass.

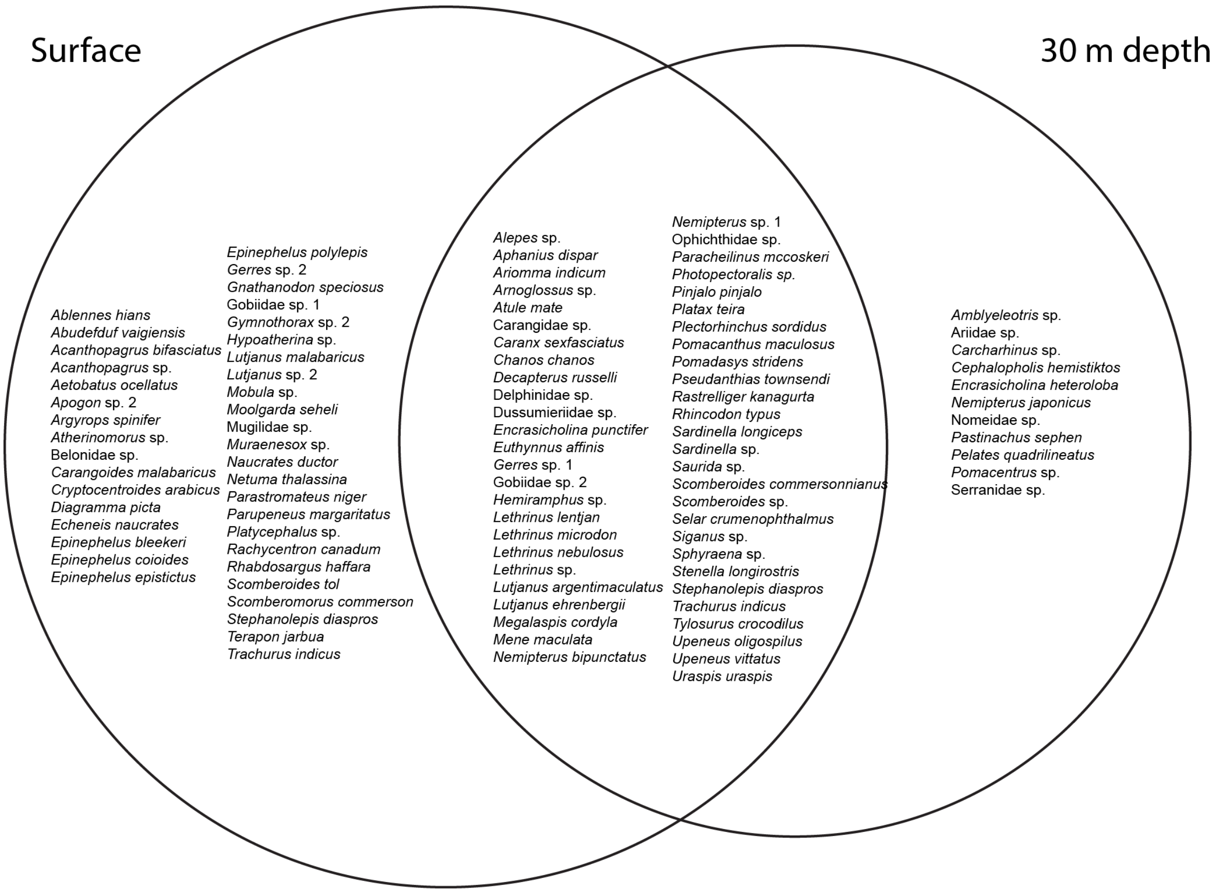

Appendix 5 Venn diagram showing the overlap in detected species composition between eDNA samples collected at the surface and samples collected at 30 m depth (all from sand bottom sites northeast of Qatar)

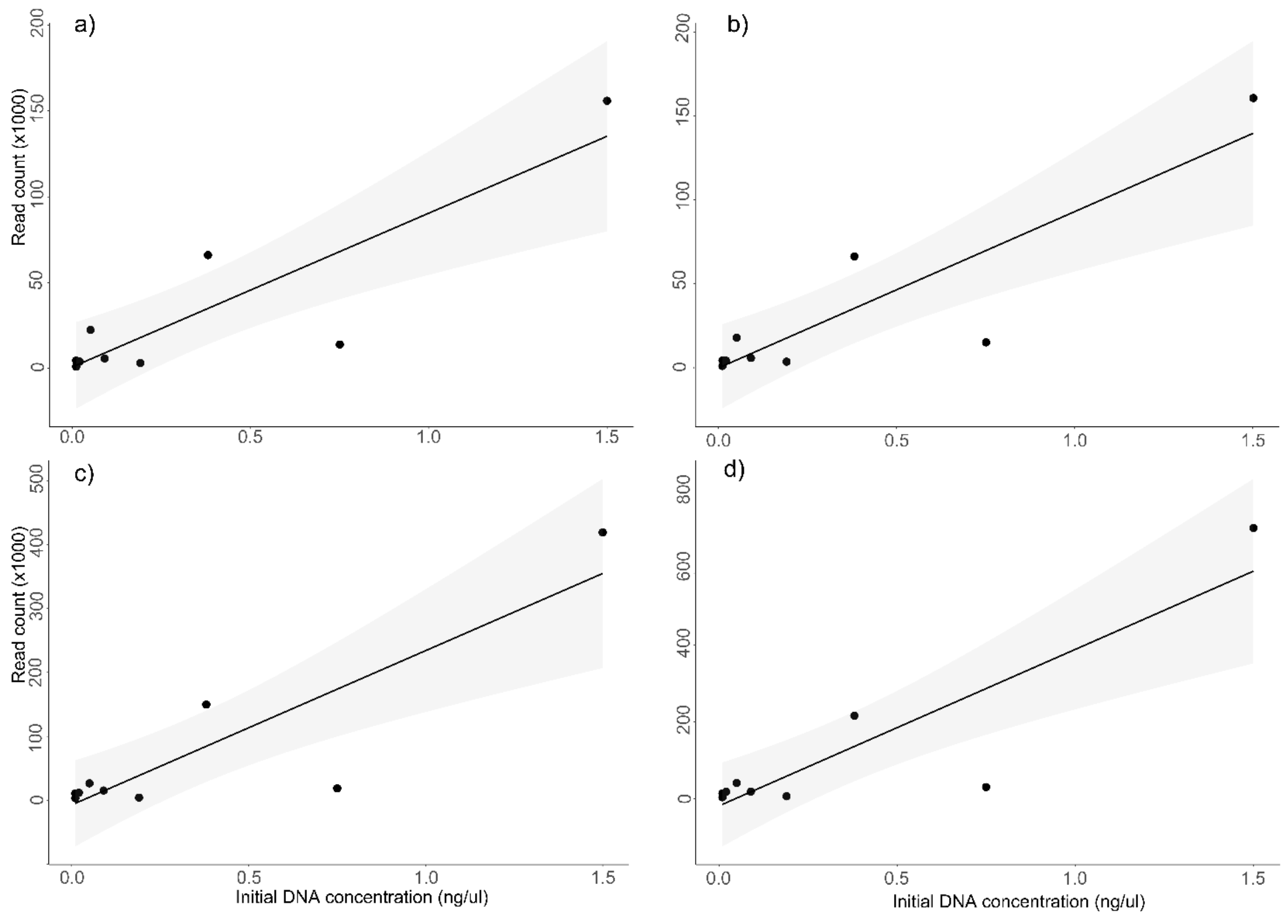

Appendix 6 Mock sample. Relationship between initial concentrations of added tissue extracts, and the read count obtained for each species after filtering with DADA2. a) Riaz barcode, 2016 ; b) Riaz barcode, 2017; c) MiFish barcode, 2016; d) MiFish barcode, 2017

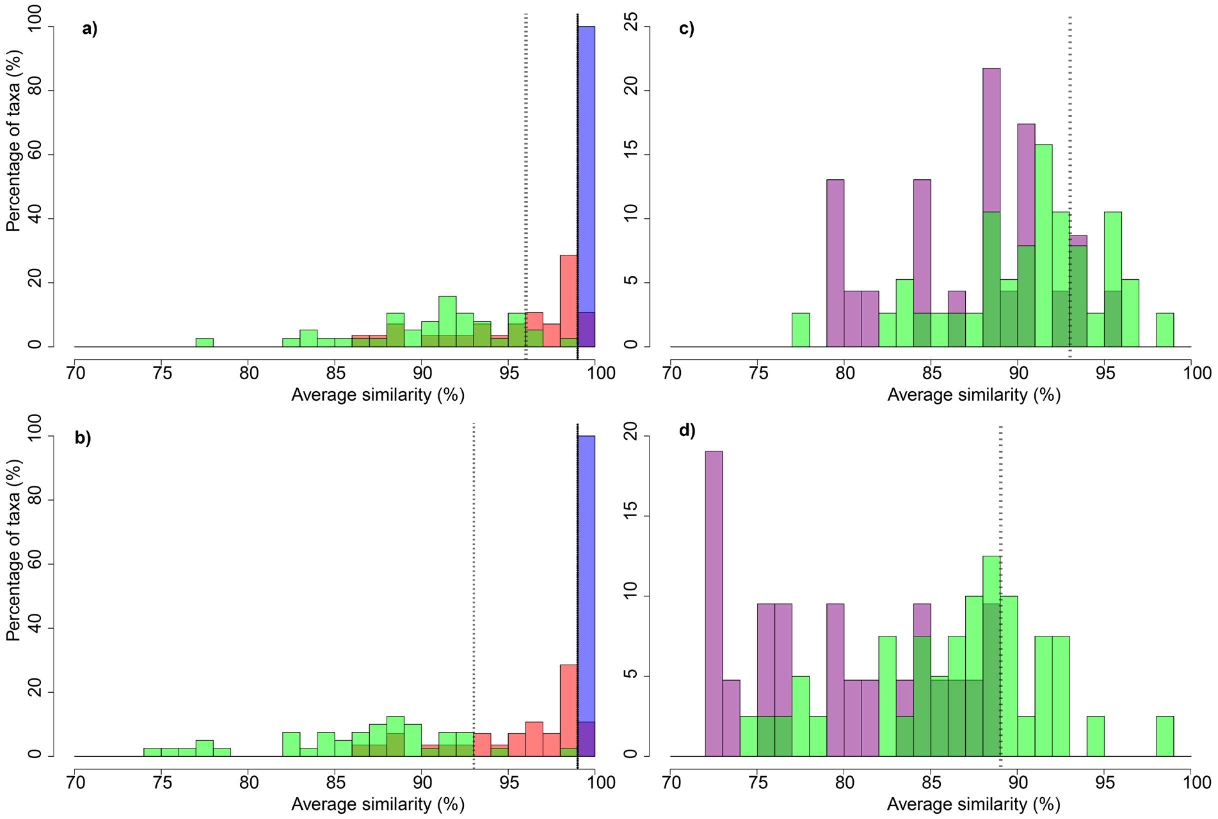

Appendix 7 Similarity thresholds for the Riaz and MiFish barcodes, respectively. **a) c)** Histograms of the minimum genetic similarity in the applied barcodes between individuals within species (blue) and the maximum similarity in the barcode between different species within genera (red) and between different genera within families (green). Dashed and solid lines show the similarity thresholds applied as a guideline for genus- and species-level assignment, respectively; **b) d)** Histograms of the maximum genetic similarity in the applied barcodes between genera within families (green) and between families within orders (purple). Dashed line shows the similarity threshold applied as a guideline for family-level assignment.

a)

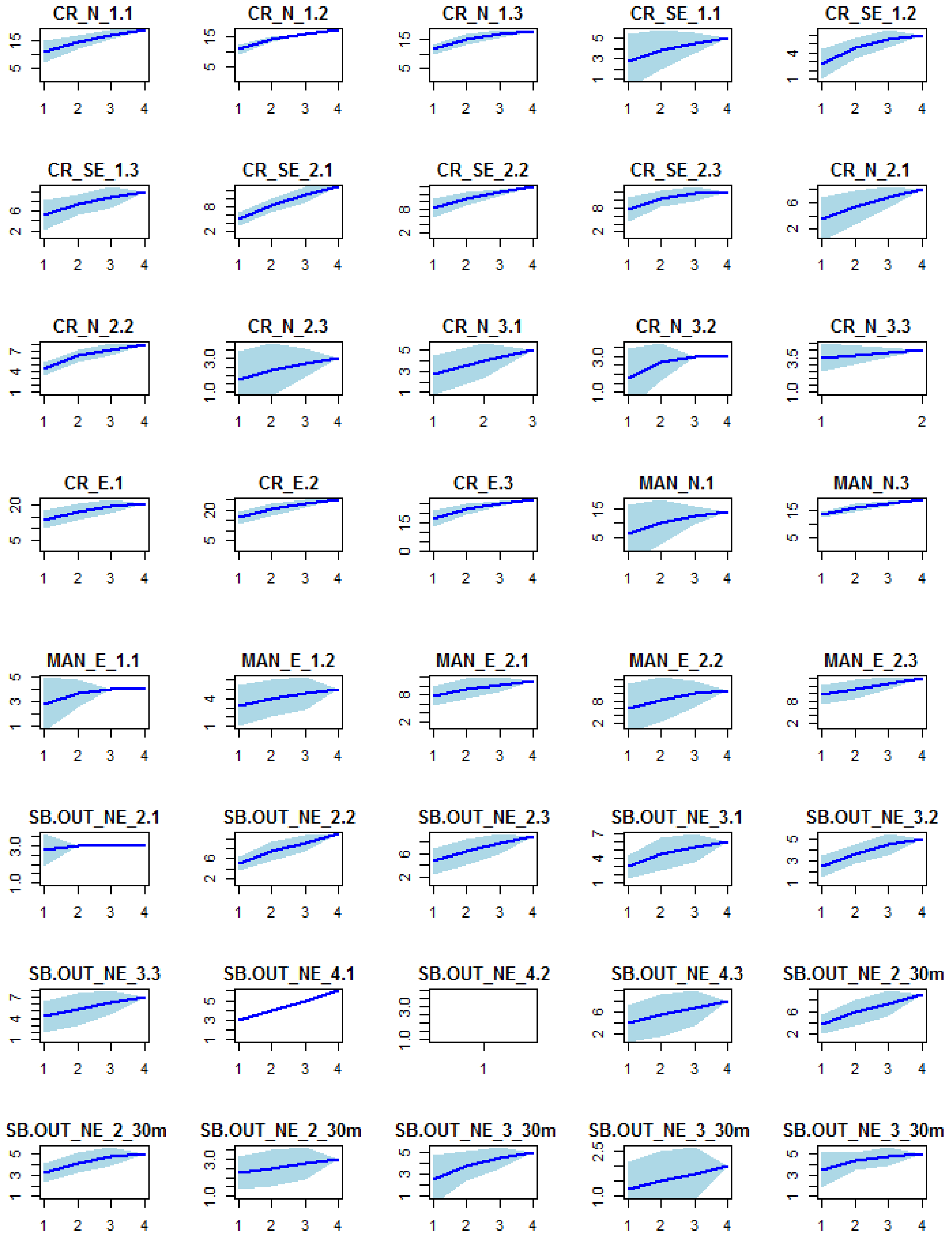

CR_N_1.1
CR_N_1.2
CR_N_1.3
CR_SE_1.1
CR_SE_1.2
CR_SE_1.3
CR_SE_2.1
CR_SE_2.2
CR_SE_2.3
CR_N_2.1
CR_N_2.2
CR_N_2.3
CR_N_3.1
CR_N_3.2
CR_N_3.3
CR_E.1
CR_E.2
CR_E.3
MAN_N.1
MAN_N.3
MAN_E_1.1
MAN_E_1.2
MAN_E_2.1
MAN_E_2.2
MAN_E_2.3
SB.OUT_NE_2.1
SB.OUT_NE_2.2
SB.OUT_NE_2.3
SB.OUT_NE_3.1
SB.OUT_NE_3.2
SB.OUT_NE_3.3
SB.OUT_NE_4.1
SB.OUT_NE_4.2
SB.OUT_NE_4.3
SB.OUT_NE_2_30m
SB.OUT_NE_2_30m
SB.OUT_NE_2_30m
SB.OUT_NE_3_30m
SB.OUT_NE_3_30m
SB.OUT_NE_3_30m

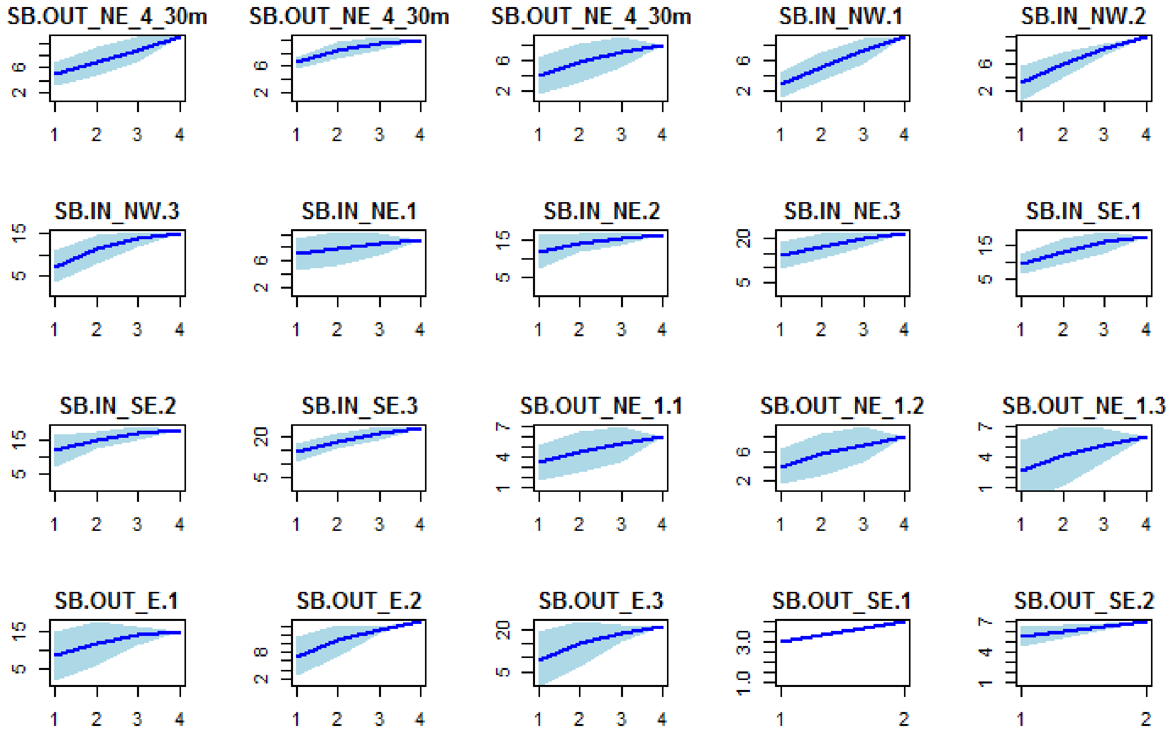
SB.OUT_NE_4_30m
SB.OUT_NE_4_30m
SB.OUT_NE_4_30m
SB.IN_NW.1
SB.IN_NW.2
SB.IN_NW.3
SB.IN_NE.1
SB.IN_NE.2
SB.IN_NE.3
SB.IN_SE.1
SB.IN_SE.2
SB.IN_SE.3
SB.OUT_NE_1.1
SB.OUT_NE_1.2
SB.OUT_NE_1.3
SB.OUT_E.1
SB.OUT_E.2
SB.OUT_E.3
SB.OUT_SE.1
SB.OUT_SE.2

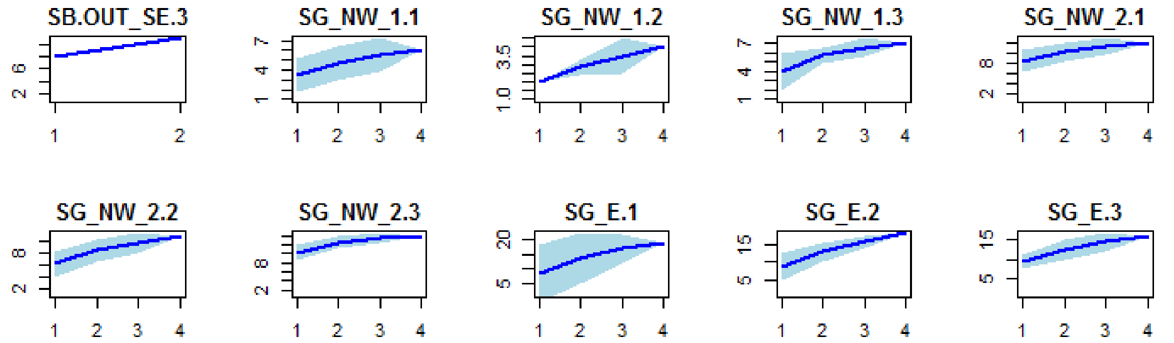
SB.OUT_SE.3
SG_NW_1.1
SG_NW_1.2
SG_NW_1.3
SG_NW_2.1
SG_NW_2.2
SG_NW_2.3
SG_E.1
SG_E.2
SG_E.3

b)

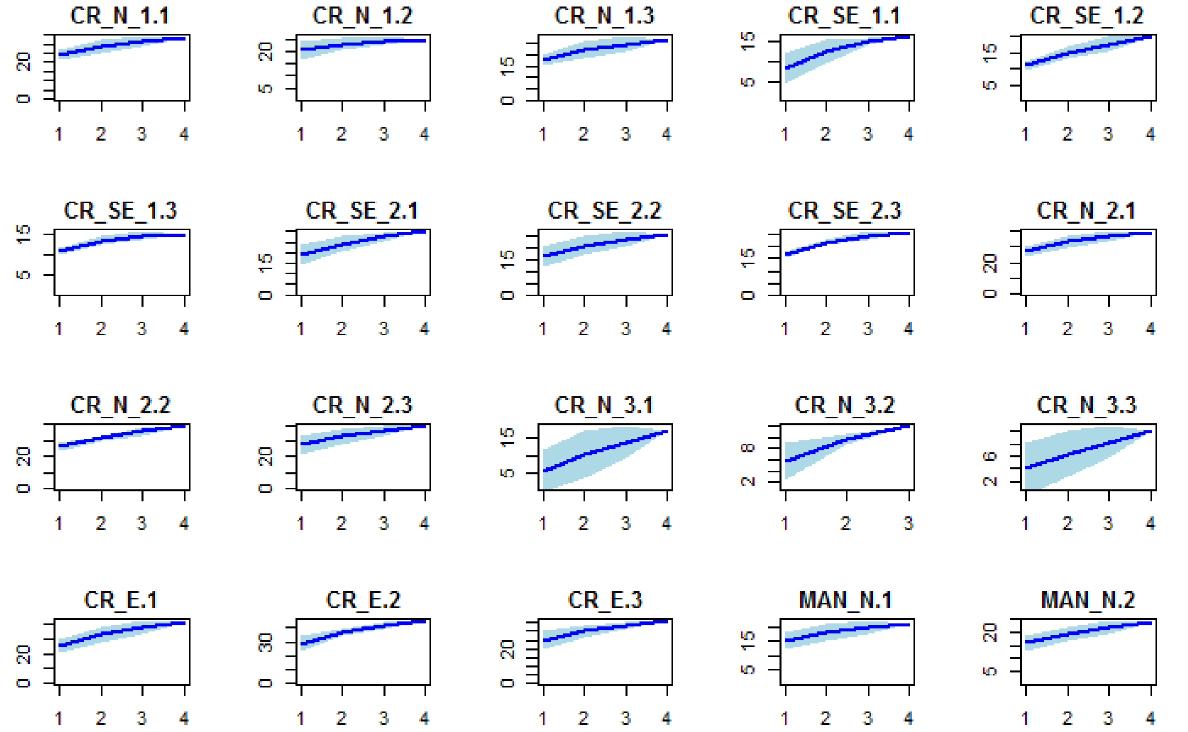
CR_N_1.1
CR_N_1.2
CR_N_1.3
CR_SE_1.1
CR_SE_1.2
CR_SE_1.3
CR_SE_2.1
CR_SE_2.2
CR_SE_2.3
CR_N_2.1
CR_N_2.2
CR_N_2.3
CR_N_3.1
CR_N_3.2
CR_N_3.3
CR_E.1
CR_E.2
CR_E.3
MAN_N.1
MAN_N.2

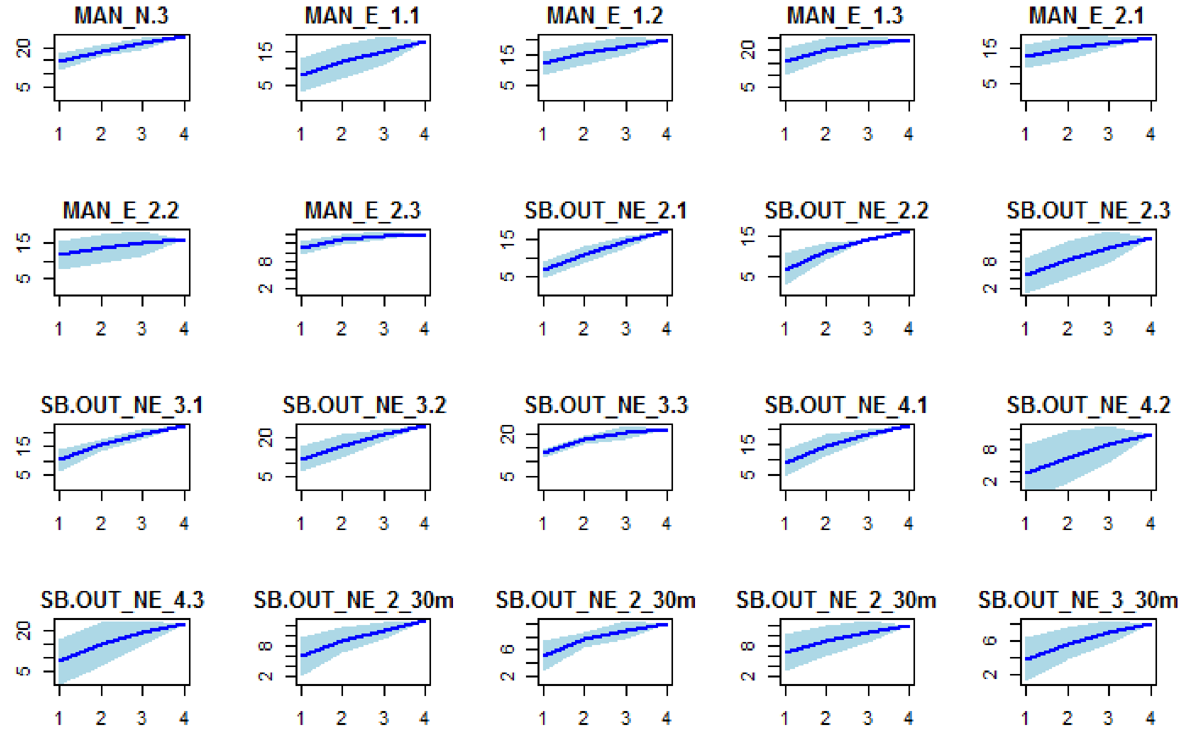
MAN_N.3
MAN_E_1.1
MAN_E_1.2
MAN_E_1.3
MAN_E_2.1
MAN_E_2.2
MAN_E_2.3
SB.OUT_NE_2.1
SB.OUT_NE_2.2
SB.OUT_NE_2.3
SB.OUT_NE_3.1
SB.OUT_NE_3.2
SB.OUT_NE_3.3
SB.OUT_NE_4.1
SB.OUT_NE_4.2
SB.OUT_NE_4.3
SB.OUT_NE_2_30m
SB.OUT_NE_2_30m
SB.OUT_NE_2_30m
SB.OUT_NE_3_30m

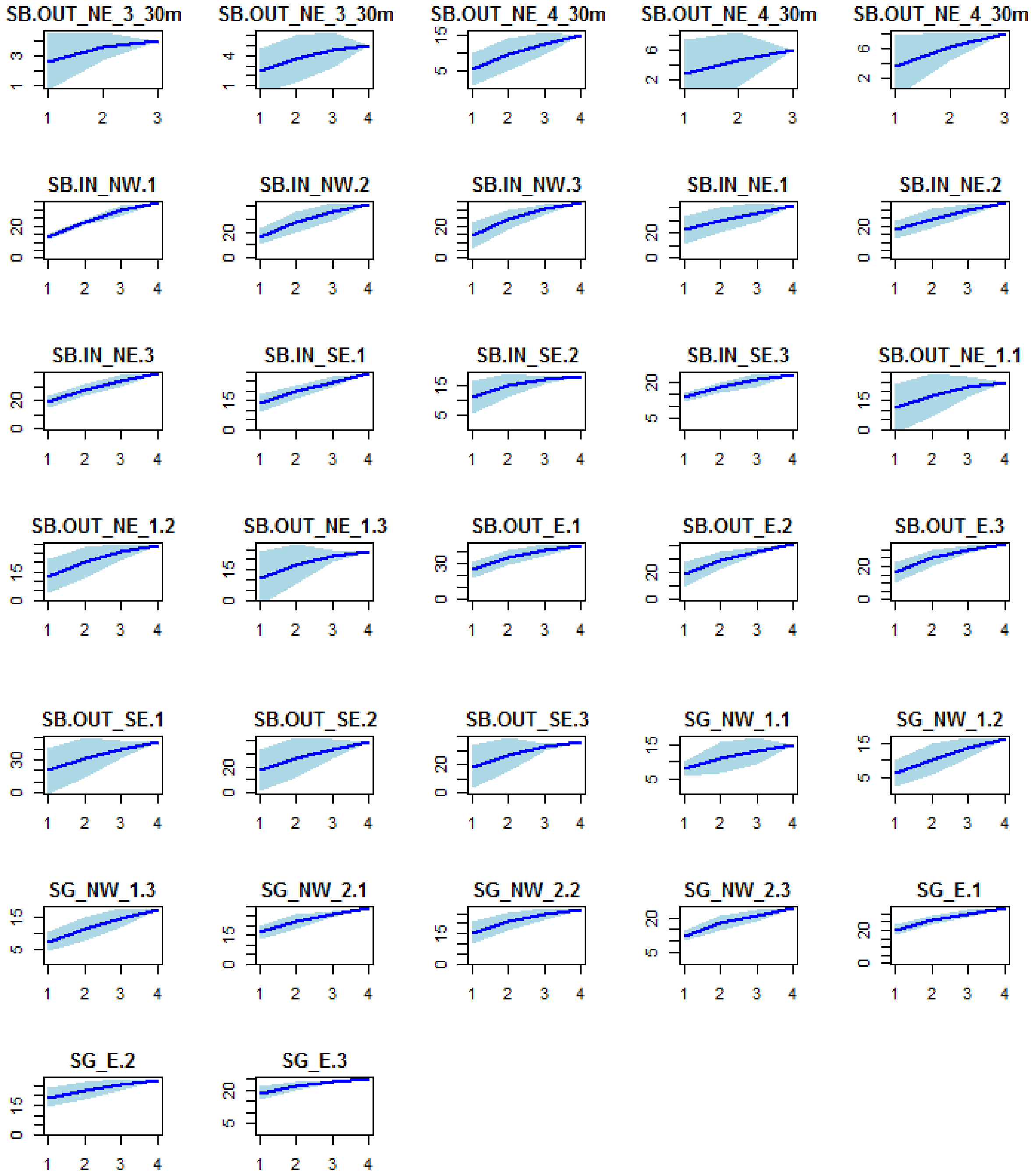
SB.OUT_NE_3_30m
SB.OUT_NE_3_30m
SB.OUT_NE_4_30m
SB.OUT_NE_4_30m
SB.OUT_NE_4_30m
SB.IN_NW.1
SB.IN_NW.2
SB.IN_NW.3
SB.IN_NE.1
SB.IN_NE.2
SB.IN_NE.3
SB.IN_SE.1
SB.IN_SE.2
SB.IN_SE.3
SB.OUT_NE_1.1
SB.OUT_NE_1.2
SB.OUT_NE_1.3
SB.OUT_E.1
SB.OUT_E.2
SB.OUT_E.3
SB.OUT_SE.1
SB.OUT_SE.2
SB.OUT_SE.3
SG_NW_1.1
SG_NW_1.2
SG_NW_1.3
SG_NW_2.1
SG_NW_2.2
SG_NW_2.3
SG_E.1
SG_E.2
SG_E.3

c)

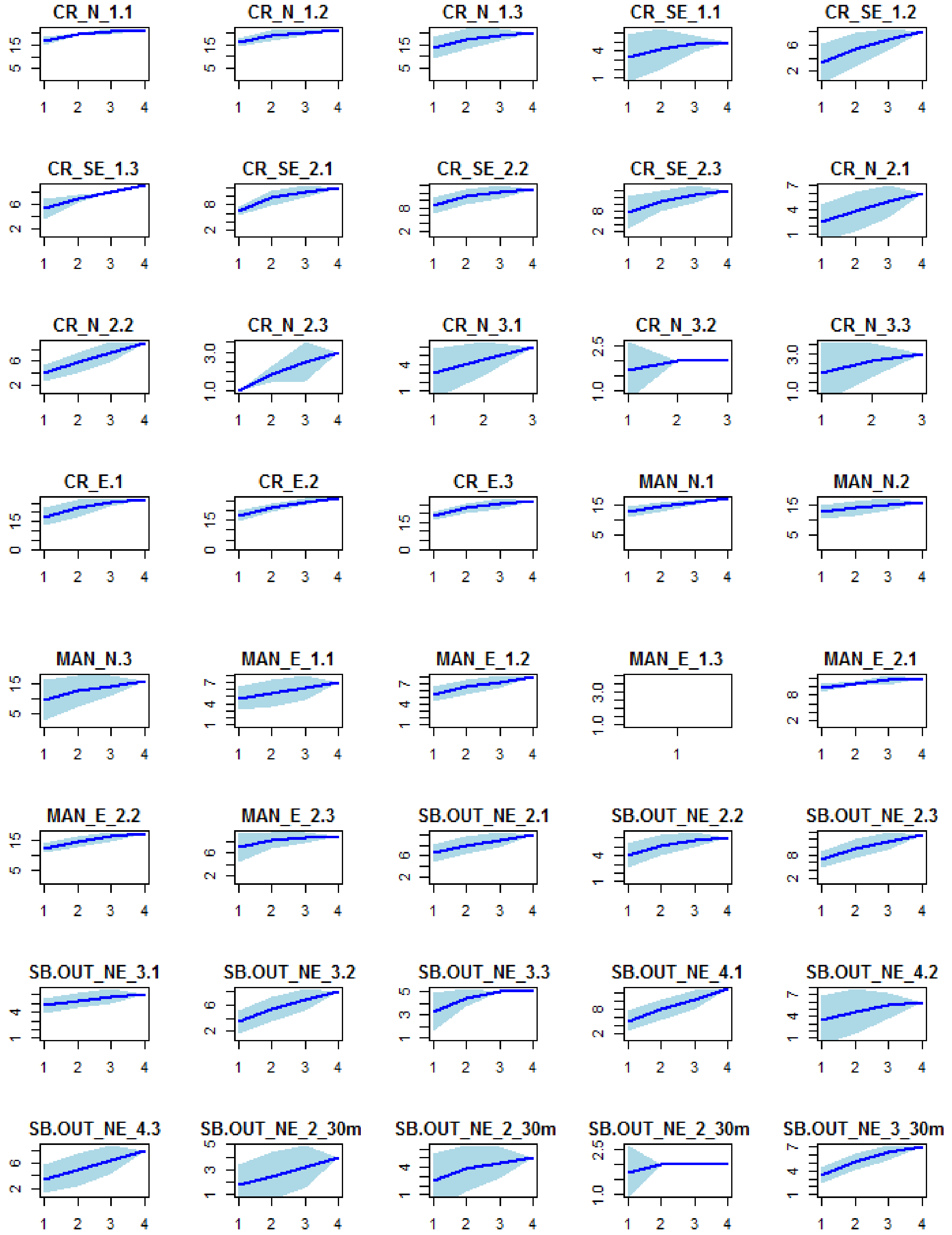
CR_N_1.1
CR_N_1.2
CR_N_1.3
CR_SE_1.1
CR_SE_1.2
CR_SE_1.3
CR_SE_2.1
CR_SE_2.2
CR_SE_2.3
CR_N_2.1
CR_N_2.2
CR_N_2.3
CR_N_3.1
CR_N_3.2
CR_N_3.3
CR_E.1
CR_E.2
CR_E.3
MAN_N.1
MAN_N.2
MAN_N.3
MAN_E_1.1
MAN_E_1.2
MAN_E_1.3
MAN_E_2.1
MAN_E_2.2
MAN_E_2.3
SB.OUT_NE_2.1
SB.OUT_NE_2.2
SB.OUT_NE_2.3
SB.OUT_NE_3.1
SB.OUT_NE_3.2
SB.OUT_NE_3.3
SB.OUT_NE_4.1
SB.OUT_NE_4.2
SB.OUT_NE_4.3
SB.OUT_NE_2_30m
SB.OUT_NE_2_30m
SB.OUT_NE_2_30m
SB.OUT_NE_3_30m

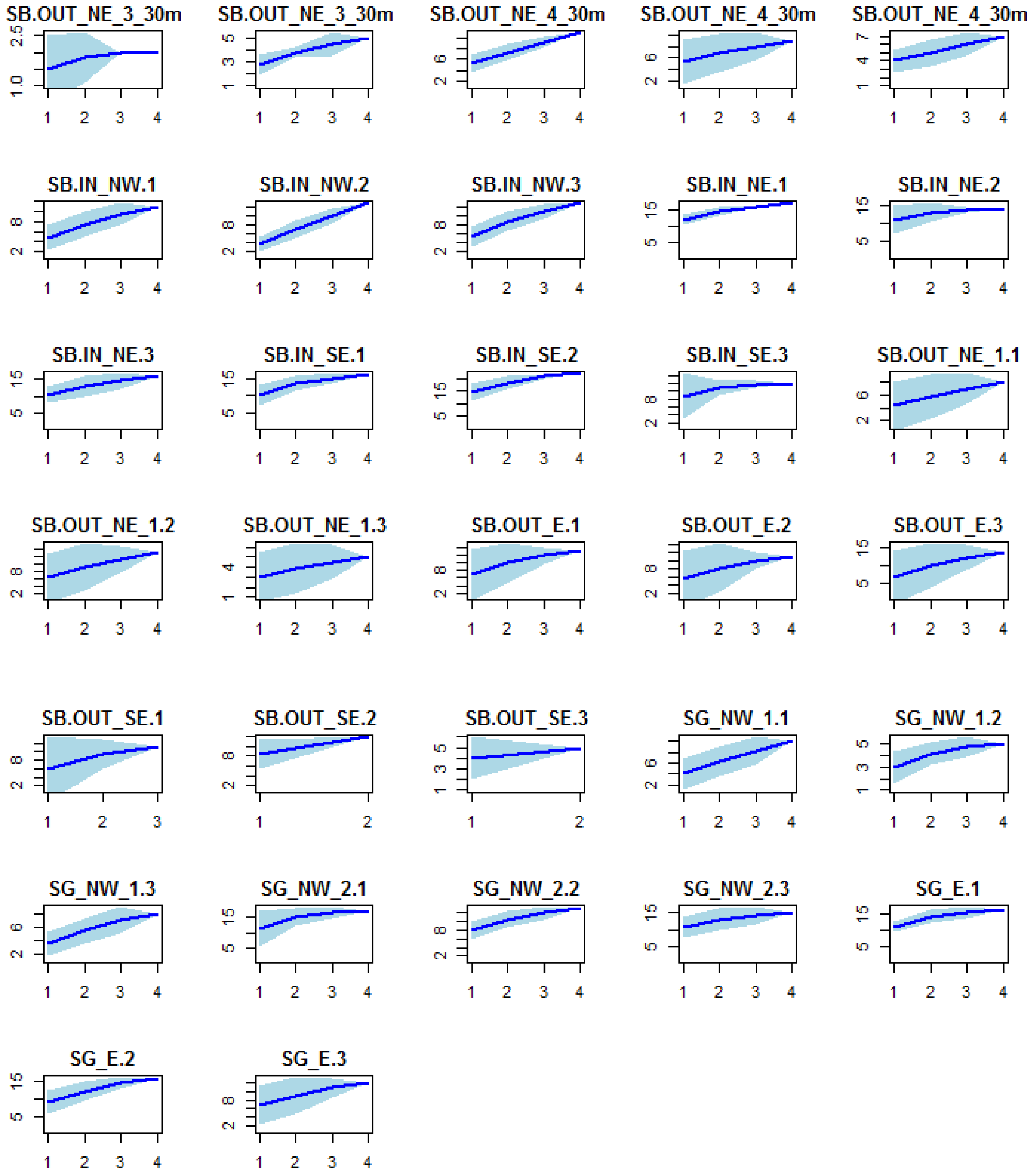
SB.OUT_NE_3_30m
SB.OUT_NE_3_30m
SB.OUT_NE_4_30m
SB.OUT_NE_4_30m
SB.OUT_NE_4_30m
SB.IN_NW.1
SB.IN_NW.2
SB.IN_NW.3
SB.IN_NE.1
SB.IN_NE.2
SB.IN_NE.3
SB.IN_SE.1
SB.IN_SE.2
SB.IN_SE.3
SB.OUT_NE_1.1
SB.OUT_NE_1.2
SB.OUT_NE_1.3
SB.OUT_E.1
SB.OUT_E.2
SB.OUT_E.3
SB.OUT_SE.1
SB.OUT_SE.2
SB.OUT_SE.3
SG_NW_1.1
SG_NW_1.2
SG_NW_1.3
SG_NW_2.1
SG_NW_2.2
SG_NW_2.3
SG_E.1
SG_E.2
SG_E.3

d)

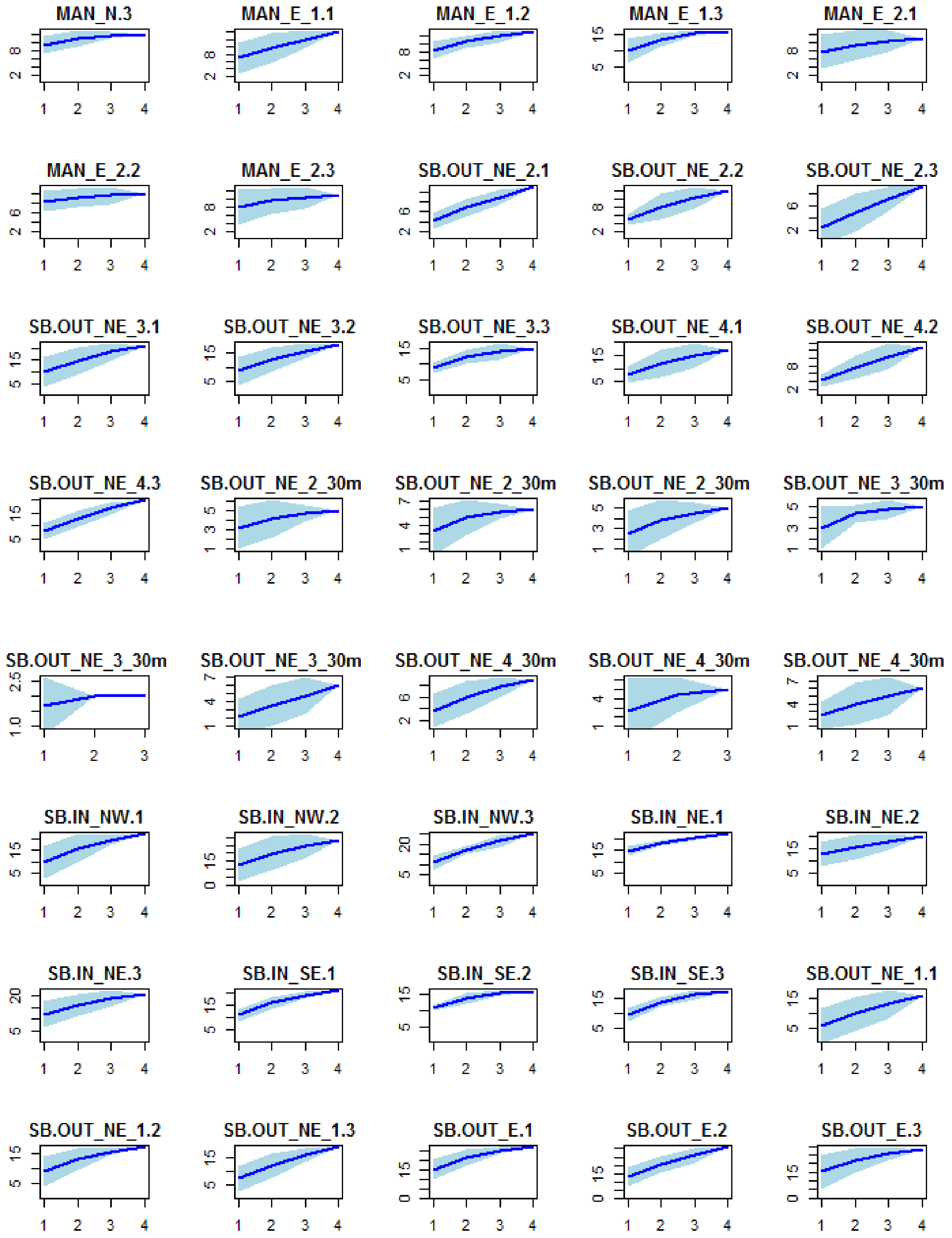
MAN_N.3
MAN_E_1.1
MAN_E_1.2
MAN_E_1.3
MAN_E_2.1
MAN_E_2.2
MAN_E_2.3
SB.OUT_NE_2.1
SB.OUT_NE_2.2
SB.OUT_NE_2.3
SB.OUT_NE_3.1
SB.OUT_NE_3.2
SB.OUT_NE_3.3
SB.OUT_NE_4.1
SB.OUT_NE_4.2
SB.OUT_NE_4.3
SB.OUT_NE_2_30m
SB.OUT_NE_2_30m
SB.OUT_NE_2_30m
SB.OUT_NE_3_30m
SB.OUT_NE_3_30m
SB.OUT_NE_3_30m
SB.OUT_NE_4_30m
SB.OUT_NE_4_30m
SB.OUT_NE_4_30m
SB.IN_NW.1
SB.IN_NW.2
SB.IN_NW.3
SB.IN_NE.1
SB.IN_NE.2
SB.IN_NE.3
SB.IN_SE.1
SB.IN_SE.2
SB.IN_SE.3
SB.OUT_NE_1.1
SB.OUT_NE_1.2
SB.OUT_NE_1.3
SB.OUT_E.1
SB.OUT_E.2
SB.OUT_E.3

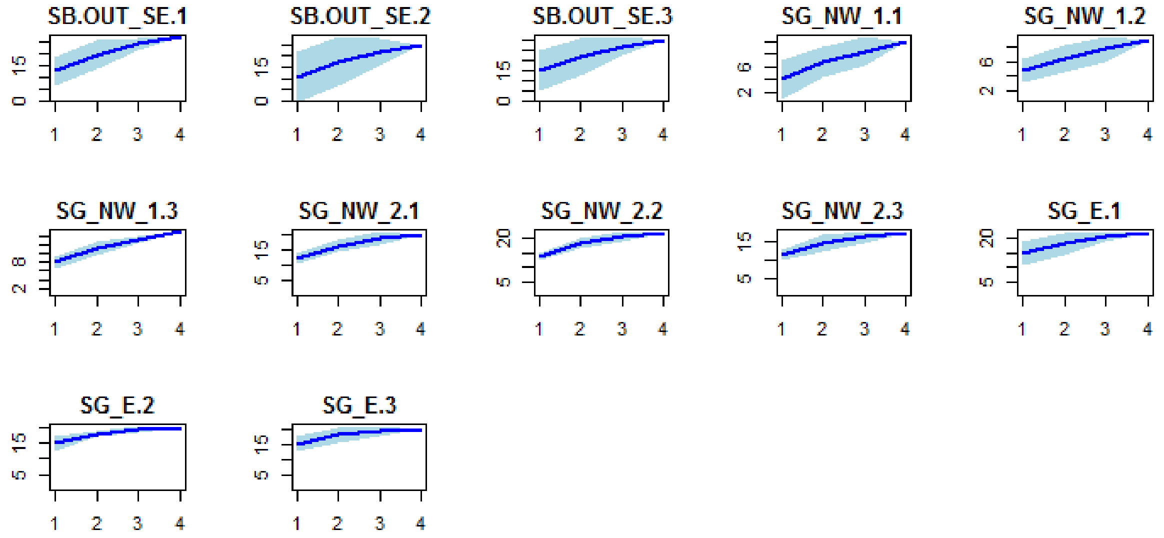

Appendix 8 Species accumulation curves for individual eDNA samples, with accumulation by PCR replicates. a) MiFish barcode, 2016; b) MiFish barcode, 2017; c) Riaz barcode, 2016; d) Riaz barcode, 2017. CR: Coral reef; MAN: Mangrove; SB_IN: Sand bottom inshore; SB_OUT: Sand bottom offshore; SG: Seagrass.

a)

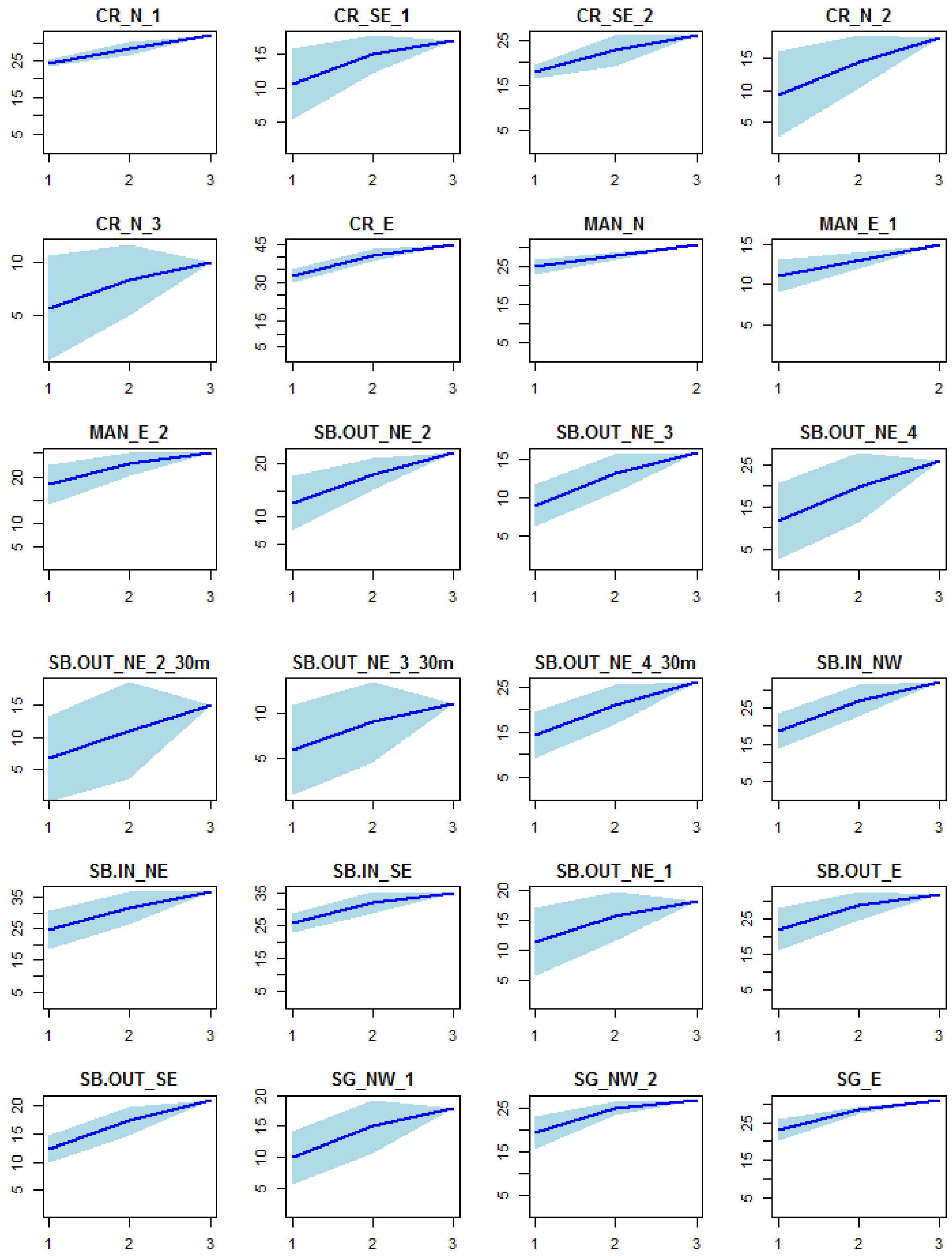
CR_N_1
CR_SE_1
CR_SE_2
CR_N_2
CR_N_3
CR_E
MAN_N
MAN_E_1
MAN_E_2
SB.OUT_NE_2
SB.OUT_NE_3
SB.OUT_NE_4
SB.OUT_NE_2_30m
SB.OUT_NE_3_30m
SB.OUT_NE_4_30m
SB.IN_NW
SB.IN_NE
SB.IN_SE
SB.OUT_NE_1
SB.OUT_E
SB.OUT_SE
SG_NW_1
SG_NW_2
SG_E

b)

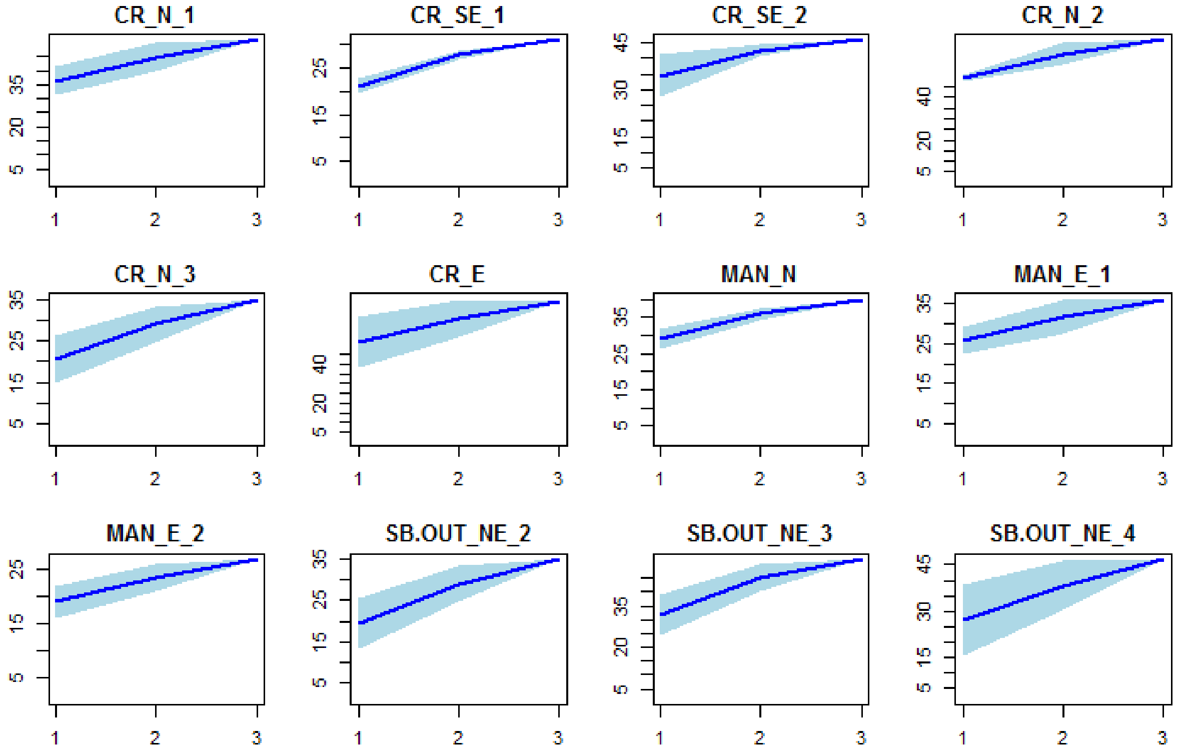

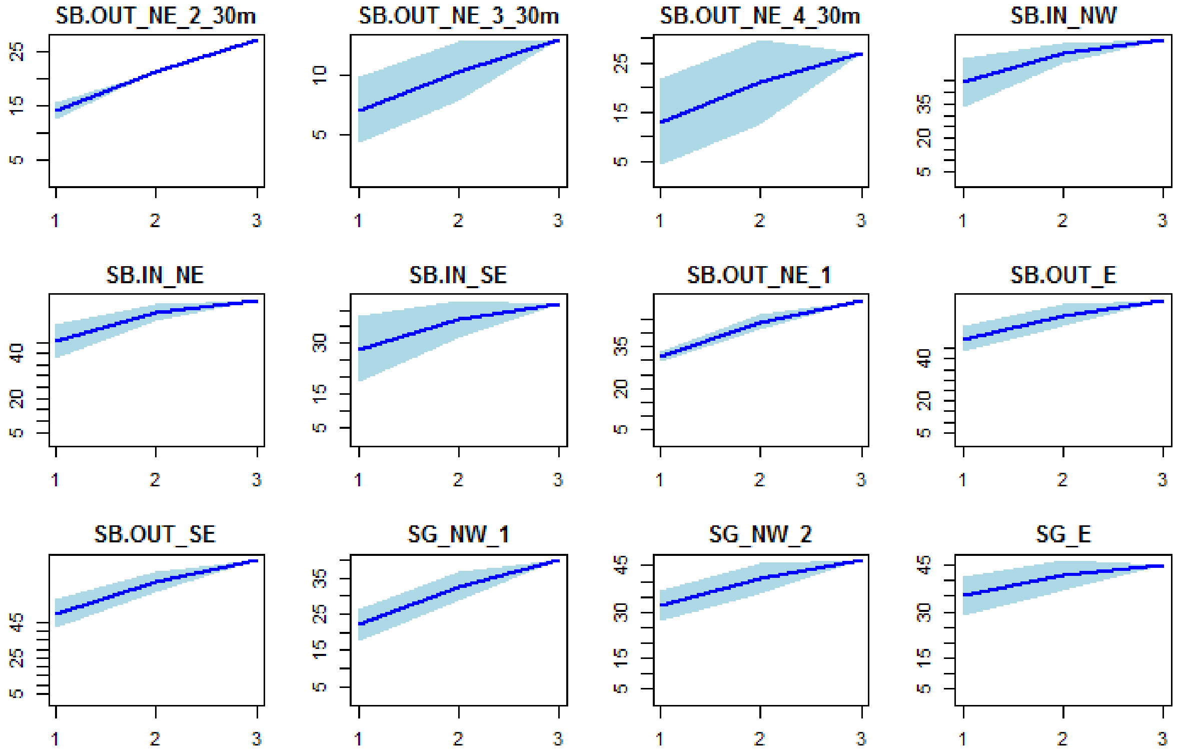

Appendix 9 Species accumulation curves for sampling sites, with accumulation by triplicate water samples. When no curve is plotted, only one sample remained in the data, or all samples contained the same taxa. a) 2016; b) 2017. CR: Coral reef; MAN: Mangrove; SB_IN: Sand bottom inshore; SB_OUT: Sand bottom offshore; SG: Seagrass.

Supplementary tables

Appendix 10. Sampling sites with dates and coordinates. Coordinates are in decimal degrees. GPS coordinates were not recorded for the field negative controls, but place names are given. CR: Coral reef; MAN: Mangrove; SB_IN: Sand bottom inshore; SB_OUT: Sand bottom offshore; SG: Seagrass.

Name	Sampling date, 2016	Sampling date, 2017	Latitude (DD)	Longitude (DD)	Habitat type	Place name
CR_E	05-09-2016	03-05-2017	25.67	52.40	Coral reef	Halul Island
CR_N_1	01-09-2016	02-05-2017	26.20	51.21	Coral reef	Al Ruwais
CR_N_2	30-08-2016	29-04-2017	26.28	50.98	Coral reef	Düvel rock/Fasht al Dibal
CR_N_3	30-08-2016	29-04-2017	26.51	51.3	Coral reef	Umm al Arshan
CR_SE_1	07-09-2016	04-05-2017	24.75	51.6	Coral reef	Al Ashat Island
CR_SE_2	07-09-2016	04-05-2017	24.78	51.77	Coral reef	Fasht al Udayd
MAN_E_1	31-08-2016	30-04-2017	25.74	51.57	Mangrove	Al Dhakhira
MAN_E_2	31-08-2016	30-04-2017	25.70	51.55	Mangrove	Al Khor
MAN_N	30-08-2016	30-04-2017	26.14	51.29	Mangrove	Al Mafjar
SB.OUT_NE_2	01-09-2016	02-05-2017	26.72	51.91	Sand bottom, offshore	Al Shaheen
SB.OUT_NE_3	01-09-2016	02-05-2017	26.63	51.95	Sand bottom, offshore	Al Shaheen
SB.OUT_NE_4	01-09-2016	02-05-2017	26.62	51.84	Sand bottom, offshore	Al Shaheen
SB.OUT_NE_2_30m	01-09-2016	02-05-2017	26.72	51.91	Sand bottom, offshore	Al Shaheen
SB.OUT_NE_3_30m	01-09-2016	02-05-2017	26.63	51.95	Sand bottom, offshore	Al Shaheen
SB.OUT_NE_4_30m	01-09-2016	02-05-2017	26.62	51.84	Sand bottom, offshore	Al Shaheen
SB.IN_NE	31-08-2016	30-04-2017	25.72	51.59	Sand bottom, inshore	NA
SB.IN_NW	30-08-2016	29-04-2017	25.97	50.97	Sand bottom, inshore	NA
SB.IN_SE	04-09-2016	06-05-2017	24.65	51.43	Sand bottom, inshore	Khor al Adaid
SB.OUT_E	05-09-2016	03-05-2017	25.49	51.97	Sand bottom, offshore	NA
SB.OUT_NE_1	01-09-2016	02-05-2017	26.46	51.58	Sand bottom, offshore	NA
SB.OUT_SE	07-09-2016	04-05-2017	24.93	51.70	Sand bottom, offshore	Close to Fasht al Ārif
SG_E	05-09-2016	03-05-2017	25.39	51.55	Seagrass	NA
SG_NW_1	30-08-2016	29-04-2017	25.83	50.86	Seagrass	NA
SG_NW_2	30-08-2016	29-04-2017	25.96	50.90	Seagrass	NA
CON_1	30-08-2016	29-04-2017	-	-	-	Ruwais Port
CON_2	01-09-2016	30-04-2017	-	-	-	Ruwais Port; Al Khor
CON_3	05-09-2016	02-05-2017	-	-	-	Doha Port; Ruwais Port
CON_4	07-09-2016	03-05-2017	-	-	-	Al Wakrah Port; Doha Port
CON_5	-	04-05-2017	-	-	-	Al Wakrah Port
CON_6	-	06-05-2017	-	-	-	Khor al Adaid

Appendix 11. Results from visual census by snorkeling or diving. The table is divided vertically in three parts for readability. CR: Coral reef; MAN: Mangrove; SB_IN: Sand bottom inshore; SB_OUT: Sand bottom offshore; SG: Seagrass.

Appendix 11, part 1)

**CR_E**<br>05-09-2016	**CR_N_1**<br>01-09-2016	**CR_N_1**<br>May-2017	**CR_SE_1**<br>07-09-2016	**CR_SE_1**<br>04-05-2017	**CR_SE_2**<br>07-09-2016
Abudefduf vaigiensis	*Abudefduf vaigiensis*	*Abudefduf vaigiensis*	*Acanthopagrus bifasciatus*	*Acanthopagrus bifasciatus*	*Acanthopagrus bifasciatus*
Acanthopagrus bifasciatus	*Acanthopagrus bifasciatus*	*Acanthopagrus bifasciatus*	*Cephalopholis hemistiktos*	*Callogobius* sp.	*Carangoides bajad*
Caragoides bajad	*Acanthurus sohal*	*Diplodus sargus*	*Chaetodon nigropunctatus*	*Carangoides bajad*	*Cheilodipterus novemstriatus*
Cephalopholis hemistiktos	*Cryptocentrus lutheri*	*Epinephelus coioides*	*Cheilodipterus novemstriatus*	*Cephalopholis hemistiktos*	*Cryptocentrus lutheri*
Chaetodon nigropunctatus	*Lutjanus fulviflamma*	*Lutjanus fulviflamma*	*Diagramma pictum*	*Chaetodon nigropunctatus*	*Epinephelus coioides*
Chaetodon melapterus	*Plectorhinchus sordidus*	*Plectorhinchus gaterinus*	*Diplodus sargus*	*Cheilodipterus novemstriatus*	*Lethrinus lentjan*
Lutjanus fulviflamma	*Pomacanthus maculosus*	*Plectorhinchus sordidus*	*Gerres oyena*	*Cryptocentrus lutheri*	*Lutjanus fulviflamma*
Siganus canaliculatus	*Scarus persicus*	*Scolopsis ghanam*	*Lutjanus fulviflamma*	*Diplodus sargus*	*Plectorhinchus sordidus*
Zebrasoma xanthurum	*Scolopsis ghanam*		*Platax teira*	*Echeneis naucrates*	*Pomacanthus maculosus*
			Plectorhichus sordidus	*Epinephelus coioides*	*Rhabdosargus sarba*
			Pomacanthus maculosus	*Gerres oyena*	*Scolopsis ghanam*
			Pomacentrus trichrourus	*Lutjanus ehrenbergii*	*Scolopsis taeniata*
			Scolopsis ghanam	*Lutjanus fulviflamma*	*Siganus canaliculatus*
				Plectorhinchus sordidus	
				Pomacanthus maculosus	
				Pomacentrus trichrourus	
				Scolopsis ghanam	
9	9	8	13	17	13

Appendix 11, part 2)

**CR_SE_2** 04-05-2017	**MAN_E_1** 31-08-2016	**MAN_E_1** 30-04-2017	**MAN_E_2** 31-08-2016	**MAN_E_2** 30-04-2017	**SB.OUT_NE_4** 01-09-2016
Acanthopagrus bifasciatus	*Aphanius dispar*	*Gerres oyena*	*Aphanius dispar*		*Echeneis naucrates*
Callogobius sp.		*Omobranchus punctatus*	*Gerres oyena*		*Remora remora*
Cephalopholis hemistiktos					*Rachycentron canadum*
Chaetodon nigropunctatus					*Rhincodon typus*
Cheilodipterus novemstriatus					
Cryptocentrus lutheri					
Epinephelus coioides					
Lutjanus fulviflamma					
Parupeneus margaritatus					
Plectorhinchus sordidus					
Pomacanthus maculosus					
Pomacentrus trichrourus					
Scolopsis ghanam					
Scolopsis taeniata					
14	1	2	2	0	4

Appendix 11, part 3)

**SB.IN_NE** 31-08-2016	**SB.IN_NE** 30-04-2017	**SB.IN_SE** 04-09-2016	**SB.IN_SE** 06-05-2017
Gerres oyena	*Cryptocentrus lutheri*	Carangidae sp.	*Acanthopagrus bifasciatus*
Lutjanus fulviflamma	*Lutjanus fulviflamma*	Clupeoid?	*Carangoides bajad*
Terapon puta	*Omobranchus punctatus*	*Gerres oyena*	*Echeneis naucrates*
	Terapon puta		*Gerres oyena*
			Upeneus oligospilus
3	4	3	5

Appendix 12. Overview of the marine vertebrate taxa detected with eDNA in the study, across habitat types and sampling years (2016 and 2017).

					% Sequence identity				Newest published records		
Class	Order	Family	Species	Common name	MiFish barcode	Riaz barcode	Photo/video documentation	Other (unpublished) observations	Qatar	Arabian/Persian Gulf	Red List status
Actinopterygii	Acanthuriformes	Acanthuridae	*Acanthurus sohal*[ab]	Sohal surgeonfish	99	100	Yes			Buchanan et al. (2016)	VU
			Zebrasoma xanthurum	Yellowtail tang	99	100	Yes			Buchanan et al. (2016)	VU
Actinopterygii	Anguilliformes	Muraenesocidae	*Muraenesox* sp.	-	99	98	No		Sivasubramanian and Ibrahim (1982) (*M. cinereus*)	Carpenter et al. (2015)	-
Actinopterygii	Anguilliformes	Muraenidae	*Gymnothorax* sp. 1	-	95	-	No		Torquato et al. (2017) (*G. undulatus*)	Carpenter et al. (2015)	-
			Gymnothorax sp. 2	-	100	100	No		Torquato et al. (2017) (*G. undulatus*)	Carpenter et al. (2015)	-
Actinopterygii	Anguilliformes	Ophichthidae	Ophichthidae sp.	-	95	100	No			Carpenter et al. (2015)	-
Actinopterygii	Atheriniformes	Atherinidae	*Atherinomorus* sp.	-	100	98	Family			Carpenter et al. (2015)	-
			Hypoatherina sp.	-	95	-	Family				-
Actinopterygii	Aulopiformes	Synodontidae	*Saurida* sp.	-	98	100	No	Trawling, 1990 (*S. undosquamis*)	Sivasubramanian and Ibrahim (1982)	Carpenter et al. (2015)	-
Actinopterygii	Batrachoidiformes	Batrachoididae	*Allenbatrachus grunniens*	Grunting toadfish	100	100	No		Bangsgaard et al. (2012)		NA
Actinopterygii	Beloniformes	Belonidae	Belonidae sp.	-	92	97	Family		Sivasubramanian and Ibrahim (1983)	Carpenter et al. (2015)	-
			Ablennes hians	Flat needlefish	97	100	Yes		Sivasubramanian and Ibrahim (1983)	Carpenter et al. (2015)	LC

			Tylosurus crocodilus	Hound needlefish	100	100	Yes	Trawling, 1990		Carpenter et al. (2015)	LC
Actinopterygii	Beloniformes	Exocoetidae	Exocoetidae sp.	-	100	100	No	Flyingfishes seen during field work	Sivasubramanian and Ibrahim (1983)	Carpenter et al. (2015)	-
			Parexocoetus mento	African sailfin flyingfish	100	100	No	Flyingfishes seen during field work		Carpenter et al. (2015)	LC
Actinopterygii	Beloniformes	Hemiramphidae	*Hemiramphus* sp.	-	100	100	Yes		Bangsgaard et al. (2012)	Carpenter et al. (2015)	NA
Actinopterygii	Blenniiformes	Blenniidae	Blenniidae sp.	-	92	-	Yes			Carpenter et al. (2015)	-
			Omobranchus punctatus	Muzzled blenny	99	100	Yes			Carpenter et al. (2015)	LC
			Petroscirtes ancylodon	Arabian fangblenny	100	100	No			Carpenter et al. (2015)	LC
Actinopterygii	Carangiformes	Carangidae	Carangidae sp.	-	-	100	Yes		Sivasubramanian and Ibrahim (1982)	Carpenter et al. (2015)	-
			Alepes sp.	-	100	100	No	Collected from fish market in Doha	El Sayed (1992)		-
			Atule mate	Yellowtail scad	100	100	No		El Sayed (1992). As *Alepes mate*	Carpenter et al. (2015)	LC
			Uraspis uraspis	Whitemouth jack	100	100	No				LC
			Carangoides malabaricus	Malabar trevally	97	100	No	Trawling, 1990	El Sayed (1992)	Carpenter et al. (2015)	LC
			Carangoides fulvoguttatus	Yellowspotted trevally	99	100	No	Collected from fish market in Doha	Sivasubramanian and Ibrahim (1982)	Carpenter et al. (2015)	LC
			Caranx sexfasciatus	Bigeye trevally	100	-	No		Torquato et al. (2017)	Carpenter et al. (2015)	LC
			Gnathanodon speciosus	Golden trevally	100	100	No	Trawling, 1990	Torquato et al. (2017)	Carpenter et al. (2015)	LC
			Decapterus russelli	Indian scad	99	(100)	No		Sivasubramanian and Ibrahim (1983) as *D. kiliche*	Carpenter et al. (2015)	LC

			Megalaspis cordyla	Torpedo scad	99	100	No			Carpenter et al. (2015)	LC
			Parastromateus niger	Black pomfret	100	100	No	Al Shaheen, 2013		Carpenter et al. (2015)	LC
			Scomberoides sp.	-	-	98	Yes		Sivasubramanian and Ibrahim (1982)	Carpenter et al. (2015)	-
			Scomberoides commersonnianus	Talang queenfish	100	-	Yes		Sivasubramanian and Ibrahim (1982)	Carpenter et al. (2015)	LC
			Scomberoides tol[a]	Needlescaled queenfish	100	-	Yes			Carpenter et al. (2015)	DD
			Selar crumenophthalmus	Bigeye scad	100	100	No		El Sayed (1992)	Carpenter et al. (2015)	LC
			Selaroides leptolepis	Yellowstripe scad	100	100	No		El Sayed (1992)	Carpenter et al. (2015)	LC
			Naucrates ductor	Pilotfish	100	-	No			Carpenter et al. (2015)	LC
			Trachurus sp.	-	(99)	(99)	No		El Sayed (1992)	Carpenter et al. (2015)	LC
Actinopterygii	Carangiformes	Echeneidae	*Echeneis naucrates*	Live sharksucker	100	100	Yes			Carpenter et al. (2015)	LC
Actinopterygii	Carangiformes	Rachycentridae	*Rachycentron canadum*	Cobia	100	100	Yes			Carpenter et al. (2015)	LC
Actinopterygii	Centrarchiformes	Terapontidae	*Pelates quadrilineatus*	Fourlined terapon	99	100	Yes	Trawling, 1990	Sivasubramanian and Ibrahim (1982)	Carpenter et al. (2015)	LC
			Terapon jarbua	Jarbua terapon	100	100	Yes		Sivasubramanian and Ibrahim (1982)	Carpenter et al. (2015)	LC
			Terapon puta	Small-scaled terapon	99	99	Yes	Trawling, 1990	Sivasubramanian and Ibrahim (1982)	Carpenter et al. (2015)	LC
Actinopterygii	Chaetodontiformes	Chaetodontidae	*Chaetodon nigropunctatus*	Black-spotted butterflyfish	100	100	Yes	Trawling, 1990	Sivasubramanian and Ibrahim (1982) as *C. obscurus*	Buchanan et al. (2016)	VU
Actinopterygii	Chaetodontiformes	Leiognathidae	*Leiognathus* sp.	-	99	-	Yes		Sivasubramanian and Ibrahim (1982)		-
			Photopectoralis	-	99	-	No				-

			sp.								
Actinopterygii	Clupeiformes	Clupeidae	Sardinella longiceps	-	99	100	No			DiBattista et al. (2016)	LC
Actinopterygii	Clupeiformes	Clupeidae	Sardinella sp.	-	100	100	No			DiBattista et al. (2016)	-
Actinopterygii	Clupeiformes	Dussumieriidae	Dussumieriidae sp.	-	99	100	No	Trawling, 1990 (*Etrumeus teres*)			-
Actinopterygii	Clupeiformes	Engraulidae	*Encrasicholina heteroloba*[a]	Shorthead anchovy	100	(100)	No			DiBattista et al. (2016)	DD
			Encrasicholina punctifer	Buccaneer anchovy	100	100	No				LC
Actinopterygii	Cyprinodontiformes	Cyprinodontidae	*Aphanius dispar*	Arabian pupfish	-	99	Yes				LC
Actinopterygii	Ephippiformes	Ephippidae	*Platax teira*	Longfin batfish	100	100	Yes				LC
Actinopterygii	Gerreiformes	Gerreidae	*Gerres* sp. 1	-	98	99	Yes	Trawling, 1990 (*G. oyena*)	Sivasubramanian and Ibrahim (1982)	Carpenter et al. (2015)	-
			Gerres sp. 2	-	96	-	Yes	Trawling, 1990 (*G. oyena*)	Sivasubramanian and Ibrahim (1982)	Carpenter et al. (2015)	-
Actinopterygii	Gobiiformes	Gobiidae	Gobiidae sp. 1	-	93	96	No			Carpenter et al. (2015)	-
			Gobiidae sp. 2	-	99	-	No			Carpenter et al. (2015)	-
			Gobiidae sp. 3	-	91	-	No			Carpenter et al. (2015)	-
			Amblyeleotris sp.	-	99	-	Yes	*A. periophthalma* at Al Shaheen, Oct-2015 (natural reef)			-
			Cryptocentroides sp.	-	(97)	-	Yes	*C. arabicus* at Al Khor, 20-May-2016		Carpenter et al. (2015)	LC
			Gnatholepis sp.	-	96	98	Yes	*G. anjerensis* at Al Shaheen, Oct-2015 (natural reef)			-

			Parachaeturichthys sp.	-	93	-	No		LSUMZ (LSU MNS) Fishes Collection no. 18094		-
			Valenciennea sp.	-	99	96	Yes	*V. persica* and *V. sexguttata* at Halul Island			-
Actinopterygii	Gonorynchiformes	Chanidae	*Chanos chanos*	Milkfish	100	100	No		Bangsgaard et al. (2012)	Carpenter et al. (2015)	LC
Actinopterygii	Kurtiformes	Apogonidae	*Fowleria* sp.	-	98	-	No				-
			Jaydia sp.	-	99	-	No				-
			Apogon sp. 1	-	98	-	Yes	*A. cyanosoma* at Al Shaheen, Oct-2015 (natural reef)			-
			Apogon sp. 2	-	96	99	Yes	*A. cyanosoma* at Al Shaheen, Oct-2015 (natural reef)			-
			Apogonichthyoides nigripinnis	Bullseye	100	100	No		Bangsgaard et al. (2012)		NA
Actinopterygii	Labriformes	Labridae	*Cheilinus lunulatus*[a]	Broomtail wrasse	-	100	No	Halul Island, 03-May-2017		Carpenter et al. (2015)	LC
			Chlorurus sordidus	Daisy parrotfish	99	100	Yes	Halul Island, 05-Sep-2016		Buchanan et al. (2016)	VU
			Paracheilinus sp.	-	(97)	(96)	No	*P. mccoskeri* at Al Shaheen, Oct-2015 (natural reef)		Carpenter et al. (2015)	LC
			Scarus ghobban	Blue-barred parrotfish	-	99	No	Trawling, 1990	Torquato et al. (2017)	Buchanan et al. (2016)	EN
			Scarus sp.	-	100	100	Yes	*S. persicus* and *S. ferrugineus* at Halul Island		Buchanan et al. (2016)	-
			Thalassoma lunare	Moon wrasse	99	100	Yes	Halul Island, 05-Sep-2016. Trawling, 1990		Carpenter et al. (2015)	LC
Actinopterygii	Lutjaniformes	Haemulidae	*Diagramma*	Painted	100	100	Yes	Trawling, 1990		Carpenter et al. (2015)	NT

			pictum	sweetlips							
			Plectorhinchus sordidus	Sordid rubberlip	99	100	Yes	Trawling, 1990		Carpenter et al. (2015)	LC
			Plectorhinchus gaterinus	Blackspotted rubberlip	100	100	Yes			Carpenter et al. (2015)	LC
			Pomadasys stridens	Striped piggy	100	100	No	Trawling, 1990		Carpenter et al. (2015)	LC
Actinopterygii	Lutjaniformes	Lutjanidae	Lutjanidae sp.	-	100	-	No			Carpenter et al. (2015)	-
			Lutjanus argentimaculatus	Mangrove red snapper	100	100	No	Photos from Al Shaheen, Oct-2015 (natural reef)		Carpenter et al. (2015)	LC
			Lutjanus fulviflamma	Dory snapper	100	100	Yes		El Sayed (1992)	Carpenter et al. (2015)	LC
			Lutjanus sp. 1	-	98	97	No	Trawling, 1990 (*L. lutjanus*)	El Sayed (1992)	Carpenter et al. (2015)	-
			Lutjanus sp. 2	-	97	-	No	Trawling, 1990 (*L. lutjanus*)	El Sayed (1992)	Carpenter et al. (2015)	-
			Lutjanus ehrenbergii	Blackspot snapper	99	100	Yes	Trawling, 1990		Carpenter et al. (2015)	LC
			Lutjanus malabaricus[a]	Malabar blood snapper	-	100	No		El Sayed (1992)	Carpenter et al. (2015)	NT
			Pinjalo pinjalo	Pinjalo	100	100	No		Torquato et al. (2017)	Carpenter et al. (2015)	LC
Actinopterygii	Mugiliformes	Mugilidae	Mugilidae sp.	-	100	100	Yes			Carpenter et al. (2015)	-
			Planiliza sp.[c]	-	98	-	Family		Sivasubramanian and Ibrahim (1983) as *Liza* spp.		-
			Planiliza macrolepis[c]	Largescale mullet	99	100	Family		Sivasubramanian and Ibrahim (1983) as *Liza macrolepis*		NA
			Chelon sp.[c]	-	99	100	Family				-
			Planiliza subviridis[c]	Greenback mullet	97	100	Family				NA

			Moolgarda seheli	Bluespot mullet	100	100	Family		Bangsgaard et al. (2012)	Carpenter et al. (2015)	LC
Actinopterygii	Perciformes	Menidae	*Mene maculata*	Moonfish	100	100	No				NA
Actinopterygii	Perciformes	Pomacanthidae	*Pomacanthus maculosus*	Yellowbar angelfish	100	100	Yes	Trawling, 1990		Carpenter et al. (2015)	LC
Actinopterygii	Perciformes	Pomacentridae	*Abudefduf vaigiensis*	Indo-Pacific sergeant	100	100	Yes			Buchanan et al. (2016)	VU
			Chromis sp. 2	-	100	100	Yes	*C. xanthopterygia* at Halul Island		Buchanan et al. (2016)	-
			Pomacentrus sp.	-	98	100	No	Halul Island, Al Ashat Island and Fasht-al-Udayd (*P. trichrourus*)		Buchanan et al. (2016)	-
Actinopterygii	Perciformes	Siganidae	*Siganus canaliculatus*	White-spotted spinefoot	100	100	Yes	Trawling, 1990	El Sayed (1992)	Carpenter et al. (2015)	LC
			Siganus sp.	-	99	100	No		El Sayed (1992)		-
Actinopterygii	Perciformes	Sillaginidae	*Sillago* sp.	-	93	97	No			Carpenter et al. (2015)	-
Actinopterygii	Perciformes	Sphyraenidae	*Sphyraena* sp.	-	98	-	Yes	*S. flavicauda* at Halul Island. Trawling, 1990	El Sayed (1992) (*S. jello* and *S. obtusata*)	Carpenter et al. (2015)	-
Actinopterygii	Perciformes	Platycephalidae	*Platycephalus* sp.	-	98	-	Yes	Fuwairit, 2015	Bangsgaard et al. (2012) (*P. indicus*)		-
Actinopterygii	Perciformes	Serranidae	Serranidae sp.	-	99	-	Yes		Krupp et al. (2000)	Carpenter et al. (2015)	-
			Aethaloperca rogaa	Redmouth grouper	100	100	Yes			Carpenter et al. (2015)	LC
			Cephalopholis hemistiktos	Yellowfin hind	100	100	Yes	Trawling, 1990		Carpenter et al. (2015)	NT
			Epinephelus bleekeri	Duskytail grouper	-	100	No			Carpenter et al. (2015)	NT
			Epinephelus polylepis	Smallscaled grouper	100	100	No			Carpenter et al. (2015)	NT

			Epinephelus coeruleopunctatus	Whitespotted grouper	-	100	Yes			Carpenter et al. (2015)	LC
			Epinephelus coioides[a]	Orange-spotted grouper	100	100	Yes			Carpenter et al. (2015)	VU
			Epinephelus sp.	-	98	-	No		Krupp et al. (2000)		-
			Epinephelus epistictus	Dotted grouper	99	98	No		Krupp et al. (2000)	Carpenter et al. (2015)	DD
			Pseudanthias sp.	-	(96)	-	No		Torquato et al. (2017)	Carpenter et al. (2015)	LC
Actinopterygii	Pleuronectiformes	Bothidae	*Bothus* sp.	-	92	98	No		Sivasubramanian and Ibrahim (1982)		-
			Arnoglossus sp.	-	93	-	No				-
Actinopterygii	Pleuronectiformes	Paralichthyidae	*Pseudorhombus* sp.	-	94	-	No			Carpenter et al. (2015)	-
Actinopterygii	Pleuronectiformes	Soleidae	Soleidae sp.	-	92	-	No			Carpenter et al. (2015)	-
			Solea sp.	-	97	-	No		Bangsgaard et al. (2012) (*S. stanalandi*)		-
Actinopterygii	Scombriformes	Ariommatidae	*Ariomma indicum*	Indian driftfish	100	100	No		Sivasubramanian and Ibrahim (1982)	Carpenter et al. (2015)	LC
Actinopterygii	Scombriformes	Nomeidae	Nomeidae sp.	-	90	-	No				-
Actinopterygii	Scombriformes	Scombridae	*Euthynnus affinis*	Kawakawa	100	100	No	Fish markets	Sivasubramanian and Ibrahim (1983)	Carpenter et al. (2015)	LC
			Rastrelliger kanagurta	Indian mackerel	100	100	No	Fish markets. Trawling, 1990	Sivasubramanian and Ibrahim (1983)	Carpenter et al. (2015)	LC
			Scomberomorus commerson[a]	Narrow-barred Spanish mackerel	100	100	No	Fish markets	El Sayed (1992)	Carpenter et al. (2015)	VU
Actinopterygii	Scombriformes	Trichiuridae	*Trichiurus lepturus*	Largehead hairtail	98	99	No	Fish markets		Carpenter et al. (2015)	LC
Actinopterygii	Siluriformes	Ariidae	Ariidae sp.	-	-	97	No	Fish markets		Carpenter et al. (2015)	-
			Netuma thalassina	Giant catfish	100	97	No	Fish markets		Carpenter et al. (2015)	LC

Actinopterygii	Siluriformes	Plotosidae	*Plotosus lineatus*	Striped eel catfish	99	96	No	Trawling, 1990	Bangsgaard et al. (2012)	Carpenter et al. (2015)	LC
Actinopterygii	Spariformes	Lethrinidae	*Lethrinus microdon*	Smalltooth emperor	99	100	No	Trawling, 1990		Carpenter et al. (2015)	LC
			Lethrinus lentjan	Pink ear emperor	99	100	Yes	Halul Island	El Sayed (1992)	Carpenter et al. (2015)	LC
			Lethrinus sp.	-	-	100	No	Trawling, 1990 (*L. borbonicus*)	El Sayed (1992)		-
			Lethrinus nebulosus	Spangled emperor	100	-	No		El Sayed (1992)	Carpenter et al. (2015)	LC
Actinopterygii	Spariformes	Nemipteridae	*Nemipterus* sp. 1	-	98	97	No	Trawling, 1990 (*N. peronii*)		Carpenter et al. (2015)	-
			Nemipterus sp. 2	-	93	-	No	Trawling, 1990 (*N. peronii*)		Carpenter et al. (2015)	-
			Nemipterus japonicus	Japanese threadfin bream	99	-	No			Carpenter et al. (2015)	LC
			Nemipterus bipunctatus	Delagoa threadfin bream	100	97	No			Carpenter et al. (2015)	LC
Actinopterygii	Spariformes	Sparidae	*Acanthopagrus* sp.	-	96	-	Yes	Trawling, 1990 (*A. berda*)		Carpenter et al. (2015)	-
			Acanthopagrus bifasciatus	Twobar seabream	(96)	100	Yes			Carpenter et al. (2015)	LC
			Argyrops spinifer	King soldier bream	100	100	No	Photo from fish market	El Sayed (1992)	Carpenter et al. (2015)	LC
			Diplodus sargus	White seabream	100	100	Yes	Trawling, 1990		Carpenter et al. (2015)	LC
			Rhabdosargus haffara	Haffara seabream	99	100	No	Trawling, 1990	Bangsgaard et al. (2012)	Carpenter et al. (2015)	NT
			Pagellus sp.	-	-	98	No		Sivasubramanian and Ibrahim (1982)		-
Actinopterygii	Syngnathiformes	Mullidae	*Parupeneus margaritatus*	Pearly goatfish	-	100	Yes	Trawling, 1990		Carpenter et al. (2015)	LC
			Upeneus sp.	-	100	-	No	Trawling, 1990	Sivasubramanian and Ibrahim (1982)		-

								(*U. sundaicus*)			
			Upeneus oligospilus[c]	Short-fin goatfish	99	100	Yes		Sivasubramanian and Ibrahim (1982) as *U. tragula*		LC
			Upeneus vittatus	Yellowstriped goatfish	99	-	No			Carpenter et al. (2015)	DD
Actinopterygii	Tetraodontiformes	Monacanthidae	*Paramonacanthus* sp.	-	99	100	No		Bangsgaard et al. (2012) (*P. oblongus*)		-
			Stephanolepis sp.	-	(100)	(100)	No	Trawling, 1990 (*S. diaspros*)		Carpenter et al. (2015)	LC
Actinopterygii	Tetraodontiformes	Tetraodontidae	*Arothron stellatus*	Stellate puffer	100	100	No		Torquato et al. (2017)	Carpenter et al. (2015)	LC
			Triacanthus biaculeatus	Short-nosed tripodfish	98	100	No			Carpenter et al. (2015)	LC
Chondrichthyes	Carcharhiniformes	Carcharhinidae	*Carcharhinus amboinensis*	Pigeye shark	99	-	No		Moore et al. (2012)	Jabado et al. (2017)	VU
			Carcharhinus sp.	-	100	-	Yes		Torquato et al. (2017) (*C. amblyrhynchoides*)	Moore et al. (2012)	-
			Carcharhinus melanopterus	Blacktip reef shark	99	-	No		Moore et al. (2012)	Jabado et al. (2017)	VU
			Negaprion acutidens	Sharptooth lemon shark	100	-	No			Moore et al. (2010)	EN
Chondrichthyes	Myliobatiformes	Dasyatidae	*Himantura uarnak*	Honeycomb stingray	99	-	No		Bangsgaard et al. (2012), Moore et al. (2012)	Jabado et al. (2017)	VU
			Himantura sp.	-	97	-	No	Trawling, 1990 (*H. gerrardi*)	Torquato et al. (2017) (*H. imbricata* and *H. bleekeri*)		-
			Pastinachus sephen	Cowtail stingray	100	-	No		Bangsgaard et al. (2012), Moore et al. (2012)	Jabado et al. (2017)	NT
			Urogymnus sp.	-	98	-	No			Jabado et al. (2017)	-
Chondrichthyes	Myliobatiformes	Myliobatidae	*Aetobatus ocellatus*[a]	Spotted eagle ray	100	-	No		Torquato et al. (2017) (*A. narinari*)	Jabado et al. (2017)	VU
			Aetomylaeus milvus	Ocellate eagle ray	100	-	No		Moore et al. (2012)		

			Mobula sp.	-	100	-	No		Moore et al. (2012)		-
			Rhinoptera javanica	Flapnose ray	100	-	No		Moore et al. (2012)		VU
Chondrichthyes	Orectolobiformes	Hemiscylliidae	Hemiscyllidae sp.	-	95	-	Yes	Trawling, 1990 (*Chiloscyllium arabicum*)	Bangsgaard et al. (2012) (*C. arabicum*), Moore et al. (2012)		-
Chondrichthyes	Orectolobiformes	Rhincodontidae	*Rhincodon typus*	Whale shark	100	-	Yes		Robinson et al. (2013)	Jabado et al. (2017)	EN
Chondrichthyes	Squaliformes	Centrophoridae	Deania sp.[d]	-	100	-	No				-
Aves	Anseriformes	Anatidae	*Anas* sp.	-	99	100			BirdLife International (2018)		-
Aves	Charadriiformes	Charadriidae	*Charadrius* sp.	-	96	99			BirdLife International (2018)		-
			Pluvialis squatarola[a]	Grey Plover	(93)	100			BirdLife International (2018)		LC
			Vanellus sp.	-	-	99			BirdLife International (2018)		-
Aves	Charadriiformes	Haematopodidae	*Haematopus ostralegus*[a]	Eurasian Oystercatcher	-	100			BirdLife International (2018)		NT
Aves	Charadriiformes	Laridae	Laridae sp.	-	-	100			BirdLife International (2018)		-
			Onychoprion anaethetus[a]	Bridled Tern	99	99	Yes		BirdLife International (2018)		LC
			Thalasseus bengalensis[c]	Lesser Crested Tern	100	100			BirdLife International (2018)		LC
			Sterna hirundo	Common Tern	-	100			BirdLife International (2018)		LC
			Sternula albifrons[a]	Little Tern	99	99			BirdLife International (2018)		LC
Aves	Charadriiformes	Scolopacidae	Scolopacidae sp.	-	-	97			BirdLife International (2018)		-
			Tringa glareola	Wood Sandpiper	(95)	100			BirdLife International (2018)		LC
			Xenus cinereus	Terek Sandpiper	100	100			BirdLife International (2018)		LC
Aves	Columbiformes	Columbidae	*Columba livia*	Rock Dove	100	100			BirdLife International (2018)		LC

			Streptopelia decaocto	-	100	-		BirdLife International (2018)	LC
Aves	Passeriformes	Passeridae	*Passer domesticus*	House Sparrow	100	100		BirdLife International (2018)	LC
Aves	Passeriformes	Pycnonotidae	*Pycnonotus* sp.	Bulbul	99	100	Yes	Qatar Bird Records Committee (2016)	-
Aves	Pelecaniformes	Ardeidae	*Ardea* sp.	-	98	99		BirdLife International (2018)	-
			Ardea cinerea	Grey Heron	100	100		BirdLife International (2018)	LC
			Butorides striata	Green-backed Heron	99	100		BirdLife International (2018)	LC
			Egretta garzetta	Little Egret	100	-		BirdLife International (2018)	LC
Aves	Pelecaniformes	Phalacrocoracidae	*Phalacrocorax* sp.	-	96	100		BirdLife International (2018)	-
Mammalia	Cetacea	Delphinidae	Delphinidae sp.	-	99	100	Yes	Bearzi et al. (2012)	-
			Sousa chinensis	Indo-Pacific humpback dolphin	100	-		Baldwin et al. (2004)	VU
			Stenella longirostris	Spinner dolphin	100	100		Bearzi et al. (2012)	DD
			Tursiops aduncus	Indo-Pacific bottlenose dolphin	100	100	Yes	Hammond et al. (2012)	DD
Mammalia	Sirenia	Dugongidae	*Dugong dugon*	Dugong	100	100		Marsh and Sobtzick (2015)	VU
Reptilia	Testudines	Cheloniidae	*Chelonia mydas*	Green turtle	100	-	Yes	Seminoff (2004)	EN

a) Taxonomic identification changed, as only one of the best matching BLAST hits corresponded to a species present in the Gulf

b) Identified as Sohal surgeonfish *Acanthurus sohal* only after manual alignment to a mitogenome on NCBI (Accession no.: KU244259)

c) Scientific name obtained from GenBank changed manually due to changes in taxonomy.

d) This detection should be regarded as highly putative, as Deania is a genus of deep-sea sharks, and neither the genus nor family has to our knowledge been recorded in the Gulf

252 **Appendix 13.** Taxon list for each habitat type. Taxa that are unique for the habitat type are highlighted in grey.

Coral reef	Mangrove	Sand offshore	Sand offshore, 30m	Sand inshore	Seagrass
Ablennes hians	*Ablennes hians*	*Ablennes hians*	*Alepes* sp.	*Ablennes hians*	*Ablennes hians*
Abudefduf vaigiensis	*Acanthopagrus bifasciatus*	*Abudefduf vaigiensis*	*Amblyeleotris* sp.	*Acanthopagrus bifasciatus*	*Abudefduf vaigiensis*
Acanthopagrus bifasciatus	Acanthopagrus sp.	*Acanthopagrus bifasciatus*	*Aphanius dispar*	*Acanthopagrus* sp.	*Acanthopagrus bifasciatus*
Acanthopagrus sp.	*Allenbatrachus grunniens*	Acanthopagrus sp.	Ariidae sp.	*Aetobatus ocellatus*	*Acanthopagrus* sp.
Acanthurus sohal	*Anas* sp.	*Acanthurus sohal*	*Ariomma indicum*	*Alepes* sp.	*Aetobatus ocellatus*
Aethaloperca rogaa	*Aphanius dispar*	*Aetobatus ocellatus*	*Arnoglossus* sp.	*Allenbatrachus grunniens*	*Alepes* sp.
Aetobatus ocellatus	*Apogonichthyoides nigripinnis*	*Aetomylaeus milvus*	*Atule mate*	*Aphanius dispar*	*Allenbatrachus grunniens*
Alepes sp.	*Ardea cinerea*	*Alepes* sp.	Carangidae sp.	*Apogon* sp. 1	*Aphanius dispar*
Allenbatrachus grunniens	*Ardea* sp.	*Allenbatrachus grunniens*	*Caranx sexfasciatus*	*Arnoglossus* sp.	*Atherinomorus* sp.
Apogon sp. 1	*Atherinomorus* sp.	*Aphanius dispar*	*Carcharhinus* sp.	*Arothron stellatus*	Belonidae sp.
Apogon sp. 2	Belonidae sp.	*Apogon* sp. 2	*Cephalopholis hemistiktos*	*Atherinomorus* sp.	*Carangoides malabaricus*
Apogonichthyoides nigripinnis	*Butorides striata*	*Apogonichthyoides nigripinnis*	*Chanos chanos*	Belonidae sp.	*Carcharhinus amboinensis*
Argyrops spinifer	*Cephalopholis hemistiktos*	*Argyrops spinifer*	*Decapterus russelli*	*Carangoides malabaricus*	*Chanos chanos*
Ariidae sp.	*Chanos chanos*	Ariidae sp.	Delphinidae sp.	*Caranx sexfasciatus*	*Chelon* sp.
Arothron stellatus	*Charadrius* sp.	*Ariomma indicum*	Dussumieriidae sp.	*Carcharhinus* sp.	*Chelonia mydas*
Atherinomorus sp.	*Chelon* sp.	*Arnoglossus* sp.	*Encrasicholina heteroloba*	*Cephalopholis hemistiktos*	*Columba livia*
Atule mate	*Columba livia*	*Arothron stellatus*	*Encrasicholina punctifer*	*Chanos chanos*	*Cryptocentroides* sp.
Belonidae sp.	*Cryptocentroides* sp.	*Atherinomorus* sp.	*Euthynnus affinis*	*Chelon* sp.	*Diagramma picta*
Blenniidae sp.	*Decapterus russelli*	*Atule mate*	*Gerres* sp. 1	*Chelonia mydas*	*Diplodus sargus*
Bothus sp.	*Diplodus sargus*	Belonidae sp.	Gobiidae sp. 2	*Cryptocentroides* sp.	*Dugong dugon*
Carangoides fulvoguttatus	*Egretta garzetta*	Blenniidae sp.	Hemiramphus sp.	*Decapterus russelli*	Dussumieriidae sp.
Carangoides malabaricus	*Epinephelus coioides*	*Bothus* sp.	*Lethrinus lentjan*	Delphinidae sp.	*Echeneis naucrates*
Carcharhinus melanopterus	*Euthynnus affinis*	Carangidae sp.	*Lethrinus microdon*	*Diagramma picta*	*Epinephelus coioides*
Carcharhinus sp.	Exocoetidae sp.	*Carangoides fulvoguttatus*	*Lethrinus nebulosus*	*Diplodus sargus*	*Epinephelus polylepis*
Cephalopholis hemistiktos	*Gerres* sp. 1	*Carangoides malabaricus*	*Lethrinus* sp.	*Dugong dugon*	*Euthynnus affinis*
Chaetodon nigropunctatus	*Gerres* sp. 2	*Caranx sexfasciatus*	*Lutjanus argentimaculatus*	Dussumieriidae sp.	Exocoetidae sp.
Chanos chanos	Gobiidae sp. 1	*Carcharhinus amboinensis*	*Lutjanus ehrenbergii*	*Echeneis naucrates*	*Gerres* sp. 1

Cheilinus lunulatus	*Haematopus ostralegus*	*Carcharhinus melanopterus*	*Megalaspis cordyla*	*Encrasicholina heteroloba*	*Gerres* sp. 2
Chelonia mydas	*Hemiramphus* sp.	*Carcharhinus* sp.	*Mene maculata*	*Encrasicholina punctifer*	*Gnathanodon speciosus*
Chlorurus sordidus	*Lethrinus lentjan*	*Cephalopholis hemistiktos*	*Nemipterus bipunctatus*	*Epinephelus coioides*	Gobiidae sp. 1
Chromis sp. 2	*Lutjanus argentimaculatus*	*Chaetodon nigropunctatus*	*Nemipterus japonicus*	*Euthynnus affinis*	Gobiidae sp. 3
Columba livia	*Lutjanus ehrenbergii*	*Chanos chanos*	*Nemipterus* sp. 1	Exocoetidae sp.	*Hemiramphus* sp.
Cryptocentroides sp.	*Lutjanus fulviflamma*	*Columba livia*	Nomeidae sp.	*Gerres* sp. 1	Hemiscyllidae sp.
Deania sp.*	*Moolgarda seheli*	*Cryptocentroides* sp.	Ophichthidae sp.	*Gerres* sp. 2	*Himantura* sp.
Decapterus russelli	Mugilidae sp.	*Decapterus russelli*	*Paracheilinus* sp.	*Gnathanodon speciosus*	*Himantura uarnak*
Delphinidae sp.	*Omobranchus punctatus*	Delphinidae sp.	*Pastinachus sephen*	Gobiidae sp. 1	*Hypoatherina* sp.
Diagramma picta	*Pagellus* sp.	*Diagramma picta*	*Pelates quadrilineatus*	*Hemiramphus* sp.	Laridae sp.
Diplodus sargus	*Parachaeturichthys* sp.	*Diplodus sargus*	*Photopectoralis* sp.	Hemiscyllidae sp.	*Lethrinus lentjan*
Dussumieriidae sp.	*Paramonacanthus* sp.	*Dugong dugon*	*Pinjalo pinjalo*	*Himantura* sp.	*Lethrinus microdon*
Echeneis naucrates	*Parastromateus niger*	Dussumieriidae sp.	*Platax teira*	*Himantura uarnak*	*Lethrinus nebulosus*
Encrasicholina heteroloba	*Parupeneus margaritatus*	*Echeneis naucrates*	*Plectorhinchus sordidus*	*Hypoatherina* sp.	*Lethrinus* sp.
Encrasicholina punctifer	*Pastinachus sephen*	*Encrasicholina heteroloba*	*Pomacanthus maculosus*	Laridae sp.	*Lutjanus ehrenbergii*
Epinephelus coeruleopunctatus	*Pelates quadrilineatus*	*Encrasicholina punctifer*	*Pomacentrus* sp.	*Lethrinus lentjan*	*Lutjanus fulviflamma*
Epinephelus coioides	*Petroscirtes ancylodon*	*Epinephelus bleekeri*	*Pomadasys stridens*	*Lethrinus microdon*	*Lutjanus* sp. 2
Epinephelus polylepis	*Phalacrocorax* sp.	*Epinephelus coioides*	*Pseudanthias* sp.	*Lethrinus nebulosus*	*Omobranchus punctatus*
Epinephelus sp.	*Pinjalo pinjalo*	*Epinephelus epistictus*	*Rastrelliger kanagurta*	*Lethrinus* sp.	*Pagellus* sp.
Euthynnus affinis	*Planiliza macrolepis*	*Epinephelus polylepis*	*Rhincodon typus*	*Lutjanus argentimaculatus*	*Parachaeturichthys* sp.
Exocoetidae sp.	*Planiliza* sp.	*Euthynnus affinis*	*Sardinella longiceps*	*Lutjanus ehrenbergii*	*Paramonacanthus* sp.
Fowleria sp.	*Planiliza subviridis*	Exocoetidae sp.	*Sardinella* sp.	*Lutjanus fulviflamma*	*Parexocoetus mento*
Gerres sp. 1	*Platycephalus* sp.	*Fowleria* sp.	*Saurida* sp.	*Lutjanus* sp. 2	*Parupeneus margaritatus*
Gerres sp. 2	*Pluvialis squatarola*	*Gerres* sp. 1	*Scomberoides commersonnianus*	*Moolgarda seheli*	*Passer domesticus*
Gnathanodon speciosus	*Pomacentrus* sp.	*Gerres* sp. 2	Scomberoides sp.	Mugilidae sp.	*Pastinachus sephen*
Gnatholepis sp.	*Pseudorhombus* sp.	*Gnathanodon speciosus*	*Selar crumenophthalmus*	*Nemipterus bipunctatus*	*Pelates quadrilineatus*
Gobiidae sp. 1	*Pycnonotus* sp.	Gobiidae sp. 1	Serranidae sp.	*Nemipterus japonicus*	*Petroscirtes ancylodon*
Gymnothorax sp. 1	*Rastrelliger kanagurta*	Gobiidae sp. 2	*Siganus* sp.	*Nemipterus* sp. 1	*Phalacrocorax* sp.
Gymnothorax sp. 2	*Rhabdosargus haffara*	*Gymnothorax* sp. 1	*Sphyraena* sp.	*Nemipterus* sp. 2	*Pinjalo pinjalo*
Hemiramphus sp.	*Sardinella* sp.	*Gymnothorax* sp. 2	*Stenella longirostris*	*Netuma thalassina*	*Platax teira*

Himantura uarnak	*Scomberoides commersonnianus*	*Hemiramphus* sp.	*Stephanolepis* sp.	*Omobranchus punctatus*	*Platycephalus* sp.
Hypoatherina sp.	*Scomberoides* sp.	*Himantura* sp.	*Trachurus* sp.	Ophichthidae sp.	*Plectorhinchus sordidus*
Jaydia sp.	*Siganus* sp.	*Himantura uarnak*	*Tylosurus crocodilus*	*Pagellus* sp.	*Plotosus lineatus*
Laridae sp.	*Solea* sp.	*Hypoatherina* sp.	*Upeneus oligospilus*	*Parachaeturichthys* sp.	*Pomacanthus maculosus*
Lethrinus lentjan	Soleidae sp.	*Jaydia* sp.	*Upeneus vittatus*	*Paramonacanthus* sp.	*Pomadasys stridens*
Lethrinus microdon	*Streptopelia decaocto*	Laridae sp.	*Uraspis uraspis*	*Parupeneus margaritatus*	*Rhabdosargus haffara*
Lethrinus nebulosus	*Terapon jarbua*	*Leiognathus* sp.	*Urogymnus* sp.	*Passer domesticus*	*Scomberoides commersonnianus*
Lethrinus sp.	*Terapon puta*	*Lethrinus lentjan*		*Pastinachus sephen*	*Scomberomorus commerson*
Lutjanidae sp.	*Triacanthus biaculeatus*	*Lethrinus microdon*		*Pelates quadrilineatus*	*Selaroides leptolepis*
Lutjanus argentimaculatus	*Tringa glareola*	*Lethrinus nebulosus*		*Petroscirtes ancylodon*	*Siganus canaliculatus*
Lutjanus ehrenbergii	*Tylosurus crocodilus*	*Lethrinus* sp.		*Phalacrocorax* sp.	*Siganus* sp.
Lutjanus fulviflamma	*Vanellus* sp.	*Lutjanus argentimaculatus*		*Photopectoralis* sp.	*Sillago* sp.
Lutjanus sp. 1		*Lutjanus ehrenbergii*		*Pinjalo pinjalo*	Soleidae sp.
Lutjanus sp. 2		*Lutjanus fulviflamma*		*Platax teira*	*Sphyraena* sp.
Mobula sp.		*Lutjanus malabaricus*		*Platycephalus* sp.	*Stenella longirostris*
Moolgarda seheli		*Lutjanus* sp. 1		*Plectorhinchus sordidus*	*Terapon jarbua*
Mugilidae sp.		*Lutjanus* sp. 2		*Plotosus lineatus*	*Terapon puta*
Negaprion acutidens		*Megalaspis cordyla*		*Pomacanthus maculosus*	*Thalasseus bengalensis*
Netuma thalassina		*Mene maculata*		*Pomacentrus* sp.	*Triacanthus biaculeatus*
Nomeidae sp.		*Mobula* sp.		*Pomadasys stridens*	*Tursiops aduncus*
Omobranchus punctatus		*Moolgarda seheli*		*Pseudorhombus* sp.	*Tylosurus crocodilus*
Onychoprion anaethetus		Mugilidae sp.		*Rachycentron canadum*	*Upeneus oligospilus*
Parachaeturichthys sp.		*Muraenesox* sp.		*Rastrelliger kanagurta*	*Upeneus vittatus*
Paramonacanthus sp.		*Naucrates ductor*		*Rhabdosargus haffara*	*Valenciennea* sp.
Parastromateus niger		*Nemipterus bipunctatus*		*Rhincodon typus*	
Parexocoetus mento		*Nemipterus japonicus*		*Sardinella longiceps*	
Parupeneus margaritatus		*Nemipterus* sp. 1		*Sardinella* sp.	
Passer domesticus		*Netuma thalassina*		*Saurida* sp.	
Pastinachus sephen		Nomeidae sp.		Scolopacidae sp.	
Pelates quadrilineatus		*Omobranchus punctatus*		*Scomberoides commersonnianus*	

Phalacrocorax sp.
Photopectoralis sp.
Pinjalo pinjalo
Platax teira
Plectorhinchus gaterinus
Plectorhinchus sordidus
Plotosus lineatus
Pomacanthus maculosus
Pomacentrus sp.
Rachycentron canadum
Rastrelliger kanagurta
Rhabdosargus haffara
Rhincodon typus
Rhinoptera javanica
Sardinella sp.
Saurida sp.
Scarus ghobban
Scarus sp.
Scomberoides commersonnianus
Scomberoides sp.
Scomberoides tol
Scomberomorus commerson
Selar crumenophthalmus
Selaroides leptolepis
Siganus canaliculatus
Siganus sp.
Sillago sp.
Soleidae sp.
Sousa chinensis
Sphyraena sp.

Onychoprion anaethetus
Ophichthidae sp.
Parachaeturichthys sp.
Paracheilinus sp.
Paramonacanthus sp.
Parastromateus niger
Parexocoetus mento
Parupeneus margaritatus
Pastinachus sephen
Pelates quadrilineatus
Petroscirtes ancylodon
Photopectoralis sp.
Pinjalo pinjalo
Platax teira
Platycephalus sp.
Plectorhinchus sordidus
Pomacanthus maculosus
Pomacentrus sp.
Pomadasys stridens
Pseudanthias sp.
Rachycentron canadum
Rastrelliger kanagurta
Rhabdosargus haffara
Rhincodon typus
Rhinoptera javanica
Sardinella longiceps
Sardinella sp.
Saurida sp.
Scarus sp.
Scomberoides commersonnianus

Scomberoides sp.
Selar crumenophthalmus
Selaroides leptolepis
Serranidae sp.
Siganus canaliculatus
Siganus sp.
Sillago sp.
Soleidae sp.
Sphyraena sp.
Sterna hirundo
Sternula albifrons
Terapon jarbua
Terapon puta
Thalasseus bengalensis
Triacanthus biaculeatus
Tursiops aduncus
Tylosurus crocodilus
Upeneus oligospilus
Upeneus vittatus
Urogymnus sp.
Xenus cinereus

Stenella longirostris		*Scomberoides* sp.			
Stephanolepis sp.		*Scomberoides tol*			
Sterna hirundo		*Scomberomorus commerson*			
Streptopelia decaocto		*Selar crumenophthalmus*			
Terapon puta		*Selaroides leptolepis*			
Thalasseus bengalensis		*Siganus* sp.			
Thalassoma lunare		*Sphyraena* sp.			
Trachurus sp.		*Stenella longirostris*			
Tursiops aduncus		*Stephanolepis* sp.			
Tylosurus crocodilus		*Terapon jarbua*			
Upeneus oligospilus		*Terapon puta*			
Upeneus vittatus		*Trachurus* sp.			
Valenciennea sp.		*Triacanthus biaculeatus*			
Zebrasoma xanthurum		*Trichiurus lepturus*			
		Tursiops aduncus			
		Tylosurus crocodilus			
		Upeneus oligospilus			
		Upeneus sp.			
		Upeneus vittatus			
		Uraspis uraspis			
		Valenciennea sp.			
		Zebrasoma xanthurum			
131	69	139	64	108	81

* This detection should be regarded as highly putative, as Deania is a genus of deep-sea sharks, and neither the genus nor family has to our knowledge been recorded in the Gulf

Appendix 14. Average Raup-Crick dissimilarities between sites of the same or different habitat type (means across four rarefaction runs). a) 2016; b) 2017

a)

	Coral reef	Mangrove	Sand inshore	Sand offshore	Sand offshore, 30 m	Seagrass
**Coral reef**	0.3839					
**Mangrove**	0.6248	0.0254				
**Sand/mud in**	0.4210	0.3543	0.4096			
**Sand/mud out**	0.4444	0.7018	0.6317	0.3207		
**Sand/mud 30 m**	0.6634	0.9539	0.8629	0.3306	0.0641	
**Seagrass**	0.4339	0.1733	0.1115	0.6445	0.9799	0.0013

b)

	Coral reef	Mangrove	Sand inshore	Sand offshore	Sand offshore, 30 m	Seagrass
**Coral reef**	0.3363					
**Mangrove**	0.6338	0.0005				
**Sand/mud in**	0.2531	0.3527	0.0410			
**Sand/mud out**	0.4416	0.8770	0.4215	0.2253		
**Sand/mud 30 m**	0.6980	0.9588	0.4530	0.1694	0.0084	
**Seagrass**	0.2998	0.2678	0.0094	0.5794	0.9721	0.0006

Appendix 15. Composition of the mock sample. Species names and DNA concentrations in nanograms per microliter.

**Species**	**Stock conc. (ng/µL)**	**Diluted to (ng/µL)**	**Final conc. in mock sample (ng/µL)**
Sander lucioperca	36.400	30.000	3
Barbatula barbatula	25.150	15.000	1.5
Abramis brama	12.650	7.500	0.75
Gobio gobio	11.700	3.750	0.375
Blicca bjoerkna	9.240	1.875	0.188
Gymnocephalus cernua	8.420	0.938	0.094
Thymallus thymallus	7.010	0.469	0.047
Ctenopharyngodon idella	5.545	0.234	0.023
Lota lota	5.450	0.117	0.012
Cottus poecilopus	1.535	0.059	0.006

Appendix 16. Overview of taxa that were filtered from the eDNA data, with an explanation for their removal. "Standard filter" refers to the removal of sequences found in higher abundance in negative controls than in eDNA samples, or sequences found in a single PCR replicate. Taxa which are found in the Gulf and could be true detections are highlighted in grey.

					% Sequence identity		
**Class**	**Order**	**Family**	**Species**	**Common name**	**MiFish barcode**	**Riaz barcode**	**Explanation for removal**
Actinopterygii	Carangiformes	Carangidae	*Caranx heberi*	Blacktip trevally	100	-	Standard filter
			Seriola dumerili	Greater amberjack	100	100	Standard filter
			Trachinotus blochii	Snubnose pompano	100	100	Standard filter
			Caranx sp.	-	-	100	Standard filter
Actinopterygii	Centrarchiformes	Terapontidae	Terapontidae sp.	-	94	-	Standard filter
		Kyphosidae	*Kyphosus vaigiensis*[a]	Brassy chub	100	-	Standard filter
Actinopterygii	Chaetodontiformes	Chaetodontidae	*Chaetodon* sp.	-	-	100	Standard filter
Actinopterygii	Clupeiformes	Clupeidae	*Clupea harengus*	Atlantic herring	100	-	Standard filter
Actinopterygii	Cypriniformes	Cyprinidae	Cyprinidae sp.	-	-	100	Likely lab contaminant
			Leuciscus leuciscus	Common dace	100	-	Standard filter
			Leucaspius delineatus	Belica	-	100	Likely lab contaminant
Actinopterygii	Gobiiformes	Gobiidae	*Callogobius* sp.	-	95	-	Standard filter
Actinopterygii	Kurtiformes	Apogonidae	*Cheilodipterus novemstriatus*	Indian Ocean twospot cardinalfish	-	100	Standard filter
			Ostorhinchus sp.	-	96	-	Standard filter
Actinopterygii	Labriformes	Labridae	*Leptojulis cyanopleura*	Shoulder-spot wrasse	99	-	Standard filter
			Stethojulis interrupta	Cutribbon wrasse	100	-	Standard filter
Actinopterygi	Lutjaniformes	Lutjanidae	*Caesio* sp.	-	99	-	Standard filter

i							
			Lutjanus johnii	John's snapper	100	-	Standard filter
Actinopterygii	Perciformes	Monodactylidae	*Monodactylus* sp.	-	97	-	Standard filter
		Pomacentridae	*Chromis* sp. 1	-	100		Standard filter
		Tetrarogidae	Tetrarogidae sp.	-	90	-	Standard filter
		Cyclopteridae	*Cyclopterus lumpus*	Lumpfish	-	100	Standard filter
Actinopterygii	Pleuronectiformes	Cynoglossidae	Cynoglossidae sp.	-	90	-	Standard filter
		Pleuronectidae	Pleuronectidae sp.	-	90	100	Likely lab contaminant
		Scophthalmidae	*Scophthalmus maximus*	Turbot	100	-	Standard filter
Actinopterygii	Salmoniformes	Salmonidae	*Salmo salar*	Atlantic salmon	100	100	Domesticated, likely contaminant
Actinopterygii	Siluriformes	Clariidae	*Clarias batrachus*	Philippine catfish	-	100	Standard filter
Actinopterygii	Syngnathiformes	Fistulariidae	*Fistularia petimba*	Red cornetfish	-	99	Standard filter
		Pegasidae	*Pegasus* sp.	-	-	97	Standard filter
		Syngnathidae	*Hippocampus kuda*[a]	Spotted seahorse	98	100	Standard filter
Actinopterygii	Tetraodontiformes	Balistidae	Balistidae sp.	-	-	97	Standard filter
			Abalistes stellaris	Starry triggerfish	-	100	Standard filter
		Ostraciidae	*Ostracion* sp.	-	-	99	Standard filter
		Scombridae	Thunnus sp.	-	-	100	Standard filter
		Triacanthidae	*Pseudotriacanthus strigilifer*	Long-spined tripodfish	-	100	Standard filter
Chondrichthyes	Carcharhiniformes	Carcharhinidae	*Rhizoprionodon acutus*	Milk shark	100	-	Standard filter
			Carcharhinus macloti	Hardnose shark	99	-	Standard filter
			Carcharhinus sorrah	Spot-tail shark	100	-	Standard filter

Chondrichthyes	Myliobatiformes	Dasyatidae	*Dasyatidae sp.*				Standard filter
			Brevitrygon imbricata[c]	Bengal whipray	100	-	Standard filter
Chondrichthyes	Rajiformes	Rajidae	*Raja clavata*	Thornback ray	100	-	Standard filter
Aves	Charadriiformes	Scolopacidae	*Limosa lapponica*	Bar-tailed Godwit	-	100	Standard filter
			Numenius phaeopus	Whimbrel	100	99	Standard filter
Aves	Galliformes	Phasianidae	*Gallus gallus*	Red junglefowl (chicken)	100	100	Domesticated, likely contaminant
			Meleagris gallopavo	Turkey	100	100	Domesticated, likely contaminant
Aves	Passeriformes	Sylviidae	*Acrocephalus scirpaceus*	Common Reed-warbler	-	100	Standard filter
		Alaudidae	*Alauda arvensis*	Eurasian Skylark	-	100	Standard filter
Aves	Psittaciformes	Cacatuidae	*Nymphicus hollandicus*	Cockatiel	-	100	Standard filter
Mammalia	Carnivora	Canidae	*Canis lupus*	Gray wolf (dog)	100	100	Domesticated, likely contaminant
			Vulpes vulpes	Red fox	-	100	Standard filter
Mammalia	NA	Bovidae	*Bos taurus*	Cow	100	100	Domesticated, likely contaminant
			Bos sp.	-	100	100	Domesticated, likely contaminant
			Bos mutus	Wild yak	-	100	Standard filter
			Bison sp.	-	-	98	Standard filter
			Bovidae sp.	-	-	97	Domesticated, likely contaminant
			Capra sp.	-	100	100	Domesticated, likely contaminant
			Ovis sp.	-	100	100	Domesticated, likely contaminant
Mammalia	Perissodactyla	Equidae	Equus sp.	-	100	-	Standard filter
Mammalia	NA	Camelidae	*Camelus dromedarius*		-	100	Standard filter
Mammalia	Primates	Hominidae	*Homo sapiens*	Human	100	100	Likely contaminant
Mammalia	Rodentia	Cricetidae	Microtus sp.	-	96	-	Standard filter

Mammalia	Rodentia	Muridae	*Mus musculus*	House mouse	100	100	Domesticated, likely contaminant
			Rattus rattus	Black rat	100	100	Likely contaminant
			Rattus norvegicus	Brown rat	-	100	Standard filter
Mammalia	NA	Suidae	*Sus scrofa*	Wild boar (pig)	100	100	Domesticated, likely contaminant
Demospongiae	Haplosclerida	Callyspongiidae	*Callyspongia* sp.	-	97	-	Standard filter

a) Taxonomic identification changed during manual curation

Supplementary experimental procedures

Appendix 17

Additions to the reference database

To improve the reference database of DNA sequences, tissue samples from more than 80 fish species were obtained from fish markets in Qatar, from the National Aquarium Denmark (Den Blå Planet) and the fish collection at the Natural History Museum of Denmark, and from loans/donations granted by other universities (metadata is available on NCBI GenBank, Accession no. MH248164-MH248256). DNA was extracted from tissue samples using the Qiagen DNeasy® Blood and Tissue kit following the manufacturer's spin-column protocol. PCR amplification of the barcode region was performed using the MiFish-U-F or MiFish-E-F primer (for bony fishes and elasmobranchs, respectively) (Miya et al., 2015) as the forward primer and the teleo_R primer (Valentini et al., 2016) as reverse primer thus spanning both the barcodes used in this study, as well as the teleo barcode (Valentini et al., 2016). Reactions were set up in volumes of 25 µL using 18.4 µL ddH_2O, 2.5 µL GeneAmp® 10X PCR Buffer I, 1 µL of each primer (10 µM), 1 µL dNTPs (2.5 mM), 1 µL purified DNA and 0.1 µL AmpliTaq Gold® polymerase. Cycling parameters were 95 °C for 5 minutes, followed by 30 cycles of 94 °C for 30 seconds, 50 °C for 30 seconds, and 72 °C for 1 minute, and a final extension at 72 °C for 5 minutes. Two PCR reactions were run per tissue sample, and the presence and length of PCR products was checked on a 2% agarose gel stained with GelRed™ (Biotium Inc.). PCR amplicons were Sanger sequenced at Macrogen Europe using the same primers as used for PCR. When the resulting sequences were not of sufficient quality to obtain the full MiFish barcode, an extra sequencing was done using the MiFish-U-R or MiFish-E-R primer as appropriate. The sequences obtained from the two PCR replicates of each sample were assembled and inspected manually for quality in Geneious v. 10.2.3 (Biomatters Ltd.). Barcode sequences from tissue-derived DNA extractions were obtained for 85 species in 40 families and 11 orders. Sequences covering both 12S barcodes were obtained for 79 species, while for four additional species (obtuse barracuda (*Sphyraena obtusata*), dory snapper (*Lutjanus fulviflamma*), herring scad (*Alepes vari*), and doublespotted queenfish (*Scomberoides lysan*)), a sequence was obtained for one of the two barcodes.

Amplification of eDNA samples

PCR amplification was performed using two different primer sets; the "MiFish" primers developed by (Miya et al., 2015) (MiFish-U-F: GTCGGTAAAACTCGTGCCAGC and MiFish-U-R: CATAGTGGGGTATCTAATCCCAGTTTG for bony fishes. MiFish-E-F: GTTGGTAAATCTCGTGCCAGC and MiFish-E-R: CATAGTGGGGTATCTAATCCTAGTTTG for elasmobranchs) and a primer set designed by (Riaz et al., 2011) (Riaz-D: ACTGGGATTAGATACCCC and Riaz-R: TAGAACAGGCTCCTCTAG). Both primer sets target the mitochondrial 12S gene (mtDNA-12S), amplifying a region of ~170 bp (bony fishes) or ~180 bp (elasmobranchs) for the MiFish primers and ~105 bp for the Riaz primers. We added both versions of the MiFish primer set (MiFish-U and MiFish-E) to each MiFish PCR reaction. All primers were tagged with a unique sequence of six nucleotides (designed with the software OligoTag (Coissac, 2012), preceded by two or three random nucleotides at the 5'-end to increase sequence complexity during sequencing (De Barba et al., 2014). Each water sample was assigned two tags, which were used

exclusively for this sample within a sequencing run. For future studies, it should be noted that the MiFish primers can be further optimized *in silico* (Taberlet et al. 2018).

PCR reactions were performed in volumes of 25 µL containing 15.55 µL lab grade ddH_2O, 2.5 µL GeneAmp® 10X PCR Buffer I, 2 µL dNTPs (2.5 mM), 0.5 µL of each primer (10 µM), 0.5 µL HL-dsDNase 5 U μL^{-1} (ArcticZymes, Tromsø, Norway), 0.25 µL BSA (20 mg/mL), 0.2 µL AmpliTaq Gold® polymerase and 3 µL extracted DNA. Before addition of the template DNA, the master mix was incubated for 15 minutes at 37 ºC followed by 15 minutes at 60 ºC in order for the DNase to work, and then be deactivated. Thermocycling parameters were 10 minutes at 95 ºC followed by 45 cycles of 94 ºC for 30 s, 50 ºC for 30 s, and 72 ºC for 1 minute, and a final extension for 5 minutes at 72 ºC. Four PCR replicates were run for each sample and extraction control. One PCR blank was included for every eight sample PCRs. A mock sample was made from tissue extracts of 10 Danish freshwater fishes (Appendix S2) and included in each PCR setup (six PCR setups of 56 reactions for each of the two primer sets). The DNA concentration of each tissue extract was measured on a Qubit fluorometer (Invitrogen), and the extracts were mixed so their final concentrations spanned four orders of magnitude (Appendix S2). Fragment sizes were checked on 2% agarose gel stained with GelRed™.

Library building and next-generation sequencing

PCR products were pooled for sequencing using 4 µl of PCR product per PCR replicate for eDNA samples and blanks, and 2 µL of PCR product per replicate for the mock sample. Pools were purified with the MinElute PCR Purification Kit, using the manufacturer's protocol, but eluting in 2*20 µL EB buffer, with incubation at 37 ºC for 10 minutes before each of the final centrifugations. At least 250 ng of PCR product was used to build each library, and library preparation was done using the Illumina TruSeq DNA PCR-free LT Sample Prep kit. A library blank was included throughout library building, and the concentration and fragment size distribution of the libraries was checked on an Agilent 2100 Bioanalyzer before pooling the libraries. The samples were then sequenced on the Illumina NextSeq 500 platform, using 150 bp Paired-End sequencing. The samples from 2016 were sequenced at the National High-throughput DNA Sequencing Centre in Copenhagen, while the samples collected in 2017 were sequenced at the Biotech Research and Innovation Centre at the University of Copenhagen. Sequencing was moved to a different facility, as the Illumina NextSeq 500 platform was no longer available at the National High-throughput DNA Sequencing Centre. A spike-in of PhiX was added for all sequencing runs to increase sequence diversity. The two sequencing runs (samples from 2016 and 2017, respectively), yielded 175 mio. and 138 mio. reads passing the chastity filter (ratio of highest base intensity to the sum of the highest and 2nd highest > 0.6), respectively. The percentage of bases with a quality score of ≥30 were 72% and 87%. Based on aligned PhiX sequences (1.2% and 6.2% of total reads), sequencing error rates were determined to be 2.8% for the first run and 0.6% for the second run. The amount of reads per library varied between 4% and 13% (median 7%) of the total read count (12 libraries per run).

Trawling

Trawling in 1990 was done over 4-5 days at a depth of 27-30 m, approx. 100 km northeast of Bahrain. While the precise coordinates are unknown, this should be within 100 km of the northeastern sand bottom sites sampled in the current study. The main objective of the expedition was collection of sea snakes, but effort was made to collect as many fish species as possible, though excluding the largest specimens for logistical reasons. The fish collected on the trawling expedition are preserved in ethanol at the Natural History Museum of Denmark, and were taxonomically identified by JMS based on Carpenter et al. (1997) and Smith et al. (1986).

Supplementary references

Baldwin, R., Collins, M., Van Waerebeek, K., & Minton, G. 2004. The Indo-Pacific Humpback Dolphin of the Arabian Region: A Status Review. Aquatic Mammals, 30(1), 111-124.

Bearzi, G., Bjørge, A., Forney, K., Hammond, P., Karkzmarski, L., Perrin, W., Scott, M., Wang, J., Wells, R., and Wilson, B. 2012. Stenella longirostris. The IUCN Red List of Threatened Species. http://www.iucnredlist.org/

Boyer, F., Mercier, C., Bonin, A., Le Bras, Y., Taberlet, P., and Coissac, E. (2016). obitools: a unix-inspired software package for DNA metabarcoding. *Mol. Ecol. Resour. 16*, 176–182.

Carpenter, K.E. (1997). Living marine resources of Kuwait, Eastern Saudi Arabia, Bahrain, Qatar, and the United Arab Emirates (Food & Agriculture Org.).

Carpenter, K.E., Al Muftah, A., Krupp, F., Ralph, G., Buchanan, J.R., Al-Khayat, J., and Vié, J.-C. (2015). Marine Biodiversity and Ecological Threat Patterns in the Gulf: Applied Research for Marine Conservation (IUCN).

Coissac, E. (2012). OligoTag: A Program for Designing Sets of Tags for Next-Generation Sequencing of Multiplexed Samples. In Data Production and Analysis in Population Genomics, (Humana Press, Totowa, NJ), pp. 13–31.

De Barba, M., Miquel, C., Boyer, F., Mercier, C., Rioux, D., Coissac, E., and Taberlet, P. (2014). DNA metabarcoding multiplexing and validation of data accuracy for diet assessment: application to omnivorous diet. *Mol. Ecol. Resour. 14*, 306–323.

El Sayed, A.F.M. 1992. The status of Qatar's Fisheries during 1980-1990. Qatar University Science Journal, **12**, 233-238

Hammond, P., et al. 2012. Tursiops aduncus. The IUCN Red List of Threatened Species. http://www.iucnredlist.org/

Jabado, R.W., Ghais, S.M.A., Hamza, W., Shivji, M.S., and Henderson, A.C. (2015). Shark diversity in the Arabian/Persian Gulf higher than previously thought: insights based on species composition of shark landings in the United Arab Emirates. *Mar. Biodivers. 45*, 719–731.

Jabado, R.W., Kyne, P.M., Pollom, R.A., Ebert, D.A., Simpendorfer, C.A., Ralph, G.M. and Dulvy, N.K. 2017. The conservation status of sharks, rays, and chimaeras in the Arabian Sea and adjacent waters. Abu Dhabi, UAE, and Vancouver, Canada: Environment Agency of United Arab Emirates, and IUCN Species Survival Commission Shark Specialist Group

Jawad, L.A., Al-Rassady, I. and Al-Mamry, J.M., 2013. Five new records of fishes from the Arabian Sea coasts of Oman. *Marine Biodiversity Records*, *6*.

Krupp, F., Almarri, M., Zajonz, U., Carpenter, K., Almatar, S., and Zetzsche, H. 2000. Twelve new records of fishes from the Gulf. Fauna of Arabia, **18**, 323–336.

Marsh, H., and Sobtzick, S. 2015. Dugong dugon. The IUCN Red List of Threatened Species. http://www.iucnredlist.org/

Miya, M., Sato, Y., Fukunaga, T., Sado, T., Poulsen, J.Y., Sato, K., Minamoto, T., Yamamoto, S., Yamanaka, H., Araki, H., et al. (2015). MiFish, a set of universal PCR primers for metabarcoding environmental DNA from fishes: detection of more than 230 subtropical marine species. *Open Sci. 2*, 150088.

Moore, A.B.M., McCarthy, I.D., Carvalho, G.R., and Peirce, R. (2012). Species, sex, size and male maturity composition of previously unreported elasmobranch landings in Kuwait, Qatar and Abu Dhabi Emirate. *J. Fish Biol. 80*, 1619–1642.

Qatar Bird Records Committee (QBRC) 2016. The Qatar List. http://qatarbirds.org/

Riaz, T., Shehzad, W., Viari, A., Pompanon, F., Taberlet, P., and Coissac, E. (2011). ecoPrimers: inference of new DNA barcode markers from whole genome sequence analysis. *Nucleic Acids Res.* gkr732.

RStudio Team (2016). RStudio: Integrated Development for R. RStudio, Inc., Boston, MA. URL http://www.rstudio.com/.

Seminoff, J. 2004. *Chelonia mydas*. The IUCN Red List of Threatened Species. http://www.iucnredlist.org/

Sivasubramaniam, K., and Ibrahim, M.A. 1982. Demersal fish resources around Qatar. Qatar University Science Bulletin, **2**, 1

Sivasubramaniam, K., and Ibrahim, M.A. 1983. Pelagic fish resources and their fishery around Qatar. Qatar University Science Bulletin, **3**, 297-327.

Smith, J.L.B. (1986) Smiths' sea fishes. Smith Institute of Ichthyology. First edition, first impression.

Valentini, A., Taberlet, P., Miaud, C., Civade, R., Herder, J., Thomsen, P.F., Bellemain, E., Besnard, A., Coissac, E., Boyer, F., et al. (2016). Next-generation monitoring of aquatic biodiversity using environmental DNA metabarcoding. *Mol. Ecol. 25*, 929–942.
